# Assessment of the genetic and clinical determinants of fracture risk: genome wide association and mendelian randomisation study

**DOI:** 10.1136/bmj.k3225

**Published:** 2018-08-29

**Authors:** Katerina Trajanoska, John A Morris, Ling Oei, Hou-Feng Zheng, David M Evans, Douglas P Kiel, Claes Ohlsson, J Brent Richards, Fernando Rivadeneira

**Affiliations:** 1 Department of Internal Medicine, Erasmus MC, University Medical Center, Rotterdam, Netherlands; 2 Department of Epidemiology, Erasmus MC, University Medical Center, Rotterdam, Netherlands; 3 Lady Davis Institute, Jewish General Hospital, McGill University, Montréal, Québec, Canada; 4 Department of Human Genetics, McGill University, Montréal, Québec, Canada; 5 DaP Lab, School of Life Sciences, Westlake University and Westlake Institute for Advanced Study, Hangzhou, Zhejiang, China; 6 Institute of Aging Research and the Affiliated Hospital, School of Medicine, Hangzhou Normal University, Hangzhou, Zhejiang, China; 7 Medical Research Council Integrative Epidemiology Unit, University of Bristol, Bristol, UK; 8 University of Queensland Diamantina Institute, University of Queensland, Translational Research Institute, Brisbane, Australia; 9 Institute for Aging Research, Hebrew SeniorLife, Harvard Medical School, Boston, MA, USA; 10 Department of Medicine, Beth Israel Deaconess Medical Center and Harvard Medical School, Boston, MA, USA; 11 Centre for Bone and Arthritis Research, Department of Internal Medicine, Institute of Medicine, Sahlgrenska, Gothenburg, Sweden

## Abstract

**Objectives:**

To identify the genetic determinants of fracture risk and assess the role of 15 clinical risk factors on osteoporotic fracture risk.

**Design:**

Meta-analysis of genome wide association studies (GWAS) and a two-sample mendelian randomisation approach.

**Setting:**

25 cohorts from Europe, United States, east Asia, and Australia with genome wide genotyping and fracture data.

**Participants:**

A discovery set of 37 857 fracture cases and 227 116 controls; with replication in up to 147 200 fracture cases and 150 085 controls. Fracture cases were defined as individuals (>18 years old) who had fractures at any skeletal site confirmed by medical, radiological, or questionnaire reports. Instrumental variable analyses were performed to estimate effects of 15 selected clinical risk factors for fracture in a two-sample mendelian randomisation framework, using the largest previously published GWAS meta-analysis of each risk factor.

**Results:**

Of 15 fracture associated loci identified, all were also associated with bone mineral density and mapped to genes clustering in pathways known to be critical to bone biology (eg, *SOST*, *WNT16*, and *ESR1*) or novel pathways (*FAM210A*, *GRB10*, and *ETS2*). Mendelian randomisation analyses showed a clear effect of bone mineral density on fracture risk. One standard deviation decrease in genetically determined bone mineral density of the femoral neck was associated with a 55% increase in fracture risk (odds ratio 1.55 (95% confidence interval 1.48 to 1.63; P=1.5×10^−68^). Hand grip strength was inversely associated with fracture risk, but this result was not significant after multiple testing correction. The remaining clinical risk factors (including vitamin D levels) showed no evidence for an effect on fracture.

**Conclusions:**

This large scale GWAS meta-analysis for fracture identified 15 genetic determinants of fracture, all of which also influenced bone mineral density. Among the clinical risk factors for fracture assessed, only bone mineral density showed a major causal effect on fracture. Genetic predisposition to lower levels of vitamin D and estimated calcium intake from dairy sources were not associated with fracture risk.

## Introduction

The United Nations recently predicted that the ratio of people aged 65 years and older to those aged 15-64 years will triple globally by 2100.^1^ Musculoskeletal conditions are the most common causes of severe pain and physical disability, and their prevalence will increase with the ageing of society.^2^ One of the largest musculoskeletal burdens is attributable to osteoporotic fractures, the incidence of which increases exponentially with age.^3^ Therefore, the prevention of fractures is an important public health goal.

The causes of multifactorial common diseases, such as osteoporotic fractures, include genetic and environmental influences, as well as their interactions (gene by environment, or G×E). Clinically useful risk factors for the prediction of osteoporotic fracture risk need not be necessarily causal and have been implemented by well validated risk score algorithms such as FRAX^4^ and the Garvan^5^ ^6^ fracture risk calculator. Yet, the extent to which modification of predictive clinical risk factors reduces fracture risk is not generally known. A better understanding of causal mechanisms will enable prevention strategies, direct the launch of proper clinical trials, and provide targets for effective lifestyle and pharmacological interventions. Acquiring this knowledge is particularly timely and relevant considering the increasing recognition that many individuals at high fracture risk often do not receive fracture prevention interventions.^7^

Fracture risk is a moderately heritable trait (whereby h^2^ is roughly 30%),^8^ ^9^ for which no large scale, genome wide association studies (GWAS) have been undertaken so far. Large GWAS meta-analyses can also be used to perform mendelian randomisation analyses to explore the causal effects of heritable risk factors on disease in people, while reducing bias due to confounding (because genetic variation is essentially randomly assigned at conception) or reverse causation (because allele assignment always precedes disease onset).^10^ Conceptually similar to a randomised controlled trial, the mendelian randomisation approach enables an assessment of the cumulative effect of a genetically determined exposure on fracture risk, minimising the biases that frequently weaken observational studies.

Understanding whether interventions aimed at clinical risk factors would reduce fracture risk is important, because clinicians often ensure that such risk factors are optimised in individuals at high risk of fracture. If the risk factors are not causal, then such optimisation would not decrease fracture risk. Therefore, to better understand genetic and clinical risk factors for fracture, we undertook a large scale GWAS for fracture risk in up to 264 973 participants (37 857 fracture cases) in the discovery stage and in conjunction with the largest available GWAS for clinical risk factors, determined the genetic correlation (shared heritability) of key clinical risk factors and fracture. We then performed mendelian randomisation studies to explore the causal effect of these risk factors on fracture.

## Methods

### Study populations

A total of 23 cohorts with genome wide genotyping and fracture data were recruited globally through the GEnetic Factors for OSteoporosis consortium (GEFOS; http://www.gefos.org/). These cohorts were predominantly of European descent and from Europe (n=13), North America (n=8), Australia (n=1), and east Asia (n=1; tables S1A and S2A), and included 20 439 fracture cases and 78 843 controls. After meta-analysis, replication of promising findings was performed initially in the GENOMOS consortium (18 779 cases and 32 078 controls from 29 additional studies, tables S1B and S2B). Two additional large GWAS (UK Biobank, 14 492 cases and 130 563 controls; EPIC-Norfolk study, 2926 cases and 17 710 controls) were then included in the discovery set, comprising in total 37 857 cases and 227 116 controls (aged 18-106 years, including 69% women). Genetic markers reaching genome wide significance in this expanded meta-analysis and previously reported bone mineral density markers associated with fracture^11^ were additionally replicated in 147 200 cases and 150 085 controls from 23andMe, a personal genetic company (23andMe GWAS participants were customers who consented to participate in research with self reported fracture data). Figure S1 shows the overall study design. To enable two-sample mendelian randomisation studies, we compiled summary level results from the largest available GWAS meta-analyses performed so far on a large set of clinical risk factors for fracture (table 1). All studies were approved by their respective institutional ethics review committees and all participants provided written informed consent.

**Table 1:** Fracture risk factors assessed and number of samples in each genome wide association study

Disease or trait	Total sample size
Femoral neck bone mineral density^11^	32 961
Lumbar spine bone mineral density^11^	31 800
Age at menopause^12^	69 360
Rheumatoid arthritis^13^	58 284 (14 361 cases)
Inflammatory bowel disease^14^	34 652 (12 882 cases)
Type 1 diabetes^15^	26 890 (9934 cases)
Thyroid stimulating hormone^16^	26 523
Homocysteine^17^	44 147
Grip strength^18^	142 035
Age of puberty^19^	182 416
Fasting glucose^20^ ^21^	58 074
Coronary heart disease^22^	107 432 (41 513 cases)
Type 2 diabetes^23^	56 862 (12 171 cases)
Vitamin D levels 24 25	33 996
Dairy calcium intake^26^*	171 213†

* Lactase intolerance (MCM6-rs4988235) was used as a proxy for dairy consumption.

† Effect estimates were derived from reference 26.

### Study endpoint (fracture definition)

To maximise the statistical power to detect genetic loci, we used an inclusive definition of fracture, which was successfully used in previous efforts to test bone mineral density associated variants for association with fracture^11^ ^27^ and allowed us to undertake the largest GWAS on fracture risk so far. Fracture cases were defined as those individuals (>18 years old) who had fractures at any skeletal site confirmed by medical, radiological, or questionnaire reports (table S3). Fractures of the fingers, toes, and skull as well as high trauma fractures were excluded whenever possible, although there have been some reports that even high trauma fractures are also predicted by low bone mineral density and are predictive of future low trauma fracture.^28^ ^29^ Controls were defined as individuals (>18 years old) from the same cohorts, without a history of fracture.

### Fracture GWAS meta-analysis and replication

Genome wide genotyping was performed in each cohort by use of Illumina or Affymetrix genome wide genotyping chips (table S4A) and was imputed to ensure accurate ascertainment of nearly all common genetic variation above a minor allele frequency threshold of 1%. After strict quality control criteria were applied to samples and single nucleotide polymorphisms (SNPs), we followed a consortium wide standardised analytical plan to assess the association of SNPs with risk of fracture. We used logistic regression adjusted for sex, age (simple and quadratic terms), height, and weight, testing additive (per allele) genetic effects. Before performing meta-analysis, three separate meta-analytical centres checked the data independently. All individual GWAS were corrected by genomic control before we performed a fixed effects meta-analysis using METAL software. A total of 2 539 801 autosomal SNPs present in more than two studies were meta-analysed. We took forward for replication a set of promising SNPs for de novo genotyping in 26 studies at LGC Genomics (UK), using KASP genotyping as described previously^27^ (table S4B) and tested them in three more studies (table S4C), for a total of 29 studies. Allele and genotype frequencies of all genotyped variants followed Hardy-Weinberg equilibrium proportions. To obtain unbiased estimates of effect size, all SNPs associated at a genome wide significant level (that is, P<5×10^−8^) and previously known bone mineral density fracture loci^11^ were tested for replication in the 23andMe cohort (table S4C).

### Genetic determinants of risk factors for fracture

We used the genetic determinants of 15 available clinical risk factors from the largest GWAS datasets available. Genome wide association analyses have been published for bone mineral density (femoral neck and lumbar spine),^11^ age of puberty,^19^ age at menopause,^12^ grip strength,^18^ vitamin D,^24^ ^25^ homocysteine,^17^ thyroid stimulating hormone level,^16^ fasting glucose,^20^ ^21^ type 1 diabetes,^15^ type 2 diabetes,^23^ rheumatoid arthritis,^13^ inflammatory bowel disease,^14^ and coronary artery disease.^22^ The well established lactose intolerance marker (LCT_(C/T- 13910)_ polymorphism; rs4988235)^30^ was used as a surrogate to assess long term differences in dairy derived calcium intake.^31^ Additional risk factors were considered for inclusion; however, at the time of analyses, well powered GWAS were not available for some risk factors of interest including alcohol intake,^32^ ^33^ smoking^34^ and plasma calcium levels.^35^ Body mass index^36^ was not evaluated given that the fracture discovery analysis was adjusted for body weight and height.

### Genetic correlation

We used LD score regression to estimate the genetic correlation of the selected clinical risk factors and fracture (table 1).^37^ This method estimates the degree of shared genetic risk factors between two diseases or traits, and was applied to 11 of the 15 selected risk factors for fracture (since genome wide association results were not publicly available for type 1 diabetes and thyroid stimulating hormone and dairy calcium intake). We accounted for multiple testing by using a conservative Bonferroni correction for 12 tests (that is, α=4.2×10^−3^). We also tested whether the above mentioned risk factors were genetically correlated with bone mineral density.

### Mendelian randomisation

**Fig 1:**
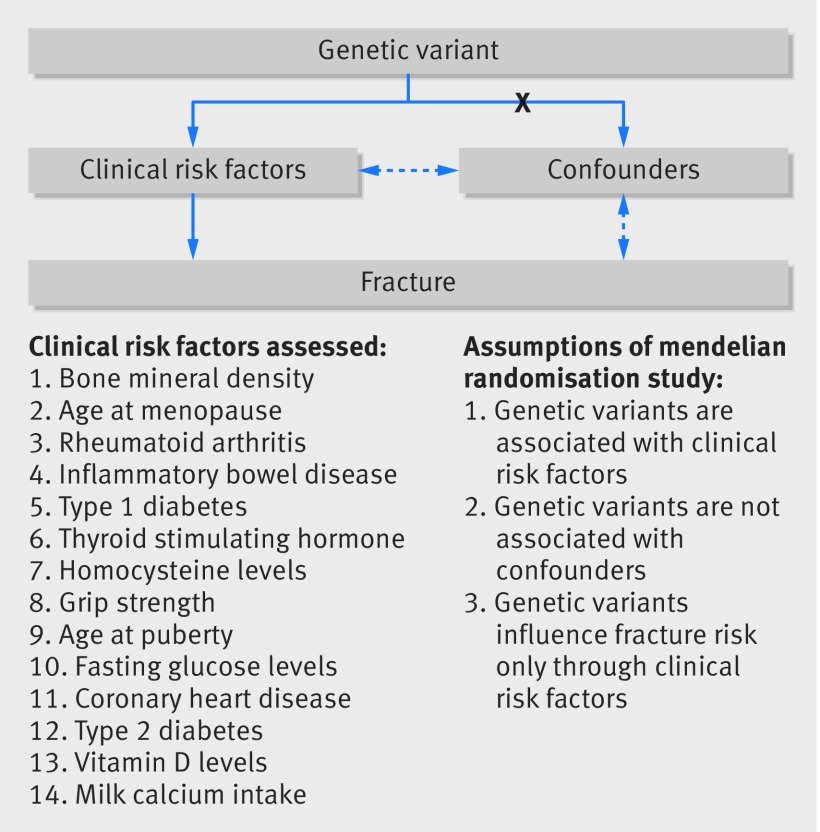
Mendelian randomisation study design

Next, we undertook mendelian randomisation analyses to estimate effects of 15 selected clinical risk factors in a two-sample mendelian randomisation framework. The mendelian randomisation approach was based on the following assumptions:

- The genetic variants used as instrumental variables are associated with the clinical risk factors.
- The genetic variants are not associated with any confounders of the exposure-outcome relation.
- The genetic variants are associated with fracture only through the clinical risk factors—that is, a lack of pleiotropy (fig 1).

We used the largest previously published GWAS meta-analyses of the risk factors, at the time of analyses, to maximise statistical power (table S5A).^38^ ^39^ ^40^ ^41^ To reduce potential bias due to population stratification, we restricted the analyses to studies with participants of European descent. To ensure independence between the SNPs used to evaluate the association of the risk factor and fracture risk, we grouped by LD (r^2^>0.05) those SNPs achieving genome wide significance, keeping only the SNP with the lowest P value per group. Next, we recorded the effect size and standard error attributed to each allele’s effect on the risk factor (table S5B). Finally, for age of menopause, we performed sex specific mendelian randomisation analysis in women only.

The resulting individual SNP effect estimates were pooled by use of the Wald type ratio estimator, which is formally analogous to an inverse weighted meta-analysis.^42^ Again, we applied a conservative Bonferroni corrected threshold (that is, α=3.3×10^−3^, because 15 risk factors were assessed) to account for the multiple risk factors tested. We also tested the assumptions underlying the mendelian randomisation approach (fig 1). To test the third assumption (a lack of pleiotropic effects of the SNPs on the outcome, independent of the exposure), we used mendelian randomisation-Egger regression.^43^ Moreover, as sensitivity analyses for robust causal inference, we additionally performed mendelian randomisation analyses using a weighted median estimator and penalised weighted median estimator. We also tested the effect of the same clinical risk factors on bone mineral density^27^ using the same methods. For the binary exposures, the odds ratios were converted (by multiplying log-odds ratios by 0.693 and then exponentiating) in order to represent the odds ratio per doubling of the odds of susceptibility to disease.^44^ Finally, we undertook mendelian randomisation power calculations^45^ for all such analyses.

### Patient involvement

No patients were directly involved in the design, recruitment, or conduct of the study. Nevertheless, several of the participating studies comprised collections of patients who were made aware of their contribution of medical data to research through their informed consents signed by all study participants. After publication, dissemination of the results will be sought across different countries involving respective patient organisations, the general public, and other stakeholders; typically, across social media, scientific meetings and media interviews. Finally, some studies sent newsletters informing their participants about important findings and their implications.

## Results

### Genetic loci associated with fracture

**Fig 2:**
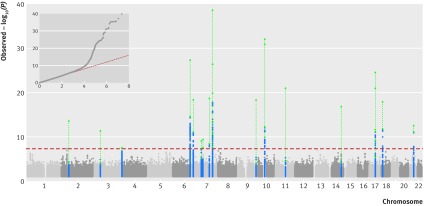
Manhattan plot of –log_10_ association P values for discovery meta-analysis, and quantile-quantile plot (QQ plot) of the distribution of observed −log_10_ association P values against the expected null distribution for discovery meta-analysis. Dashed horizontal red line=genome wide significant (GWS) threshold (P<5×10^−8^); blue dots=SNPs at GWS loci that are within 500kb of leading SNPs in previous genome wide association studies with different bone traits. Green lines and triangles=combined −log_10_ association P values after replication in the 23andMe cohort

We saw was no evidence of excessive genomic inflation (λ=1.02, LD score intercept=0.99) in the GWAS meta-analysis, suggesting that the results were not biased because of population stratification, genotyping artefacts, or cryptic family relationships (fig 2). As shown in table 2 and figure 2, 15 genomic loci were associated at a genome wide significant level with fracture risk after meta-analysis of the discovery (table S6A) and replication (tables S6B, S6C, and S6D) stages. All loci were at, or near, loci previously shown to be associated with bone mineral density,^11^ ^27^ ^46^ ^47^ ^48^ ^49^ ^50^ ^51^ ^52^ a major determinant of fracture risk (table S6E and figure S2). The effect sizes of these common SNPs on fracture risk was modest (odds ratios ranging from 1.03 to 1.10), which is consistent with GWAS findings for other complex diseases.^53^

**Table 2:** Genome wide significant single nucleotide polymorphisms (SNPs) for fracture

Locus	Candidate gene	SNP	Distance to gene (kb)	EA	EAF	Discovery stage*			Replication stage*			Combined*			
						Odds ratio (95% CI)	P		Odds ratio (95% CI)	P		Odds ratio (95% CI)	P	No of fracture cases	I^2^
2p16.2	*SPTBN1*	rs4233949	−23.21	G	0.61	1.03 (1.02 to 1.05)	6.9×10^−5^		1.04 (1.05 to 1.05)	8.9×10^−11^		1.03 (1.02 to 1.04)	2.8×10^−14^	185 057	22.4
3p22.1	*CTNNB1*	rs430727	107.2	T	0.45	1.03 (1.02 to 1.05)	1.0×10^−4^		1.03 (1.02 to 1.04)	1.1×10^−8^		1.03 (1.02 to 1.04)	5.0×10^−12^	185 057	0
6q22.33	*RSPO3*	rs10457487	0	C	0.51	1.06 (1.05 to 1.08)	2.3×10^−15^		1.04 (1.03 to 1.05)	1.7×10^−15^		1.05 (1.04 to 1.06)	4.8×10^−28^	185 057	5
6q25.1	*ESR1*	rs2982570	0	C	0.58	1.05 (1.04 to 1.07)	8.1×10^−12^		1.03 (1.02 to 1.04)	5.2×10^−10^		1.04 (1.03 to 1.05)	4.5×10^−19^	185 057	23
7q31.31	*WNT16*, *CPED1*	rs2908007	−3.25, 24.67	A	0.60	1.08 (1.06 to 1.10)	1.2×10^−20^		1.05 (1.04 to 1.06)	5.6×10^−22^		1.06 (1.05 to 1.07)	2.3×10^−39^	185 055	0
7q21.3	*C7orf76*, *SHFM1*	rs6465508	0, 0	G	0.34	1.05 (1.03 to 1.07)	4.0×10^−9^		1.04 (1.03 to 1.05)	4.1×10^−12^		1.04 (1.03 to 1.05)	2.0×10^−19^	185 056	35
7p14.1	*STARD3NL*	rs6959212	_−_89.01	T	0.34	1.04 (1.02 to 1.06)	6.9×10^−6^		1.02 (1.01 to 1.04)	1.1×10^−5^		1.03 (1.02 to 1.04)	8.8×10^−10^	185 057	15.6
7p12.1	*GRB10*, *COBL*	rs1548607	40.33, −182.4	G	0.32	1.05 (1.03 to 1.07)	3.2×10^−8^		1.02 (1.01 to 1.04)	2.1×10^−4^		1.03 (1.02 to 1.05)	4.7×10^−10^	185 052	40
9q34.11	*FUBP3*	rs7851693	0	G	0.35	1.03 (1.01 to 1.06)	1.3×10^−4^		1.05 (1.06 to 1.06)	4.8×10^−16^		1.04 (1.03 to 1.05)	5.0×10^−19^	185 057	23.5
10q21.1	*MBL2/DKK1*	rs11003047	−90.63	G	0.11	1.09 (1.07 to 1.12)	6.2×10^−12^		1.08 (1.07 to 1.10)	1.4×10^−21^		1.09 (1.07 to 1.10)	9.5×10^−33^	185 057	0
11q13.2	*LRP5*	rs3736228	0	T	0.15	1.05 (1.03 to 1.07)	3.0×10^−5^		1.07 (1.05 to 1.08)	2.8×10^−18^		1.06 (1.05 to 1.08)	1.0×10^−21^	185 056	24.6
14q32.12	*RPS6KA5*	rs1286083	0	T	0.82	1.04 (1.02 to 1.06)	8.8×10^−5^		1.05 (1.04 to 1.07)	3.0×10^−14^		1.05 (1.04 to 1.06)	1.6×10^−17^	185 085	43.3
17q21.31	*SOST*, *DUSP3*, *MEOX1*	rs2741856	−4.26, −16.65, 88.02	G	0.92	1.11 (1.08 to 1.14)	2.4×10^−12^		1.08 (1.06 to 1.11)	5.3×10^−15^		1.10 (1.07 to 1.11)	3.1×10^−25^	184 977	0
18p11.21	*FAM210A*, *RNMT*	rs4635400	0, −7.149	A	0.36	1.06 (1.04 to 1.07)	1.5×10^−12^		1.03 (1.02 to 1.04)	2.7×10^−9^		1.04 (1.03 to 1.05)	1.1×10^−18^	185 057	22
21q22.2	*ETS2*	rs9980072	141.9	G	0.73	1.06 (1.04 to 1.08)	8.4×10^−12^		1.03 (1.01 to 1.04)	1.8×10^−5^		1.04 (1.03 to 1.05)	3.4×10^−13^	185 057	36

EA=effect allele; EAF=effect allele frequency; I^2^=index of heterogeneity.

* Discovery stage (37 857 cases; 227 116 controls); replication stage (147 200 cases; 150 085 controls); combined (185 057 cases; 377 201 controls).

### Genetic correlations with clinical risk factors

SNPs influencing bone mineral density were strongly and inversely correlated with odds of fracture (table 3; genetic correlation −0.59, P=2×10^−24^ for femoral neck bone mineral density, with similar results for lumbar spine bone mineral density, −0.53, P=1×10^−20^). By contrast, none of the remaining clinical risk factors evaluated was strongly genetically correlated with risk of fracture with the exception of homocysteine (table 3). Genetically increased risk of type 2 diabetes was positively correlated with femoral neck bone mineral density, while genetically increased grip strength had positive correlations with bone mineral density of both the femoral neck and lumbar spine (table S7).

**Table 3:** Estimated genetic correlation between fracture and other clinical risk factors

Disease or trait		Genetic correlation (95%CI)	P
Femoral neck bone mineral density	−0.59 (−0.70 to −0.48)		2×10^−24^
Lumbar spine bone mineral density	−0.53 (−0.64 to −0.42)		1×10^−20^
Age at menopause	−0.12 (−0.23 to −0.003)		0.04
Rheumatoid arthritis	0.02 (−0.10 to 0.14)		0.74
Inflammatory bowel disease	−0.01 (−0.13 to 0.11)		0.90
Homocysteine levels	0.22 (0.07 to 0.37)		0.004
Grip strength	−0.10 (−0.21 to 0.01)		0.07
Age of puberty	0.03 (−0.05 to 0.11)		0.43
Fasting glucose	−0.05 (−0.19 to 0.09)		0.46
Coronary heart disease	−0.05 (−0.09 to 0.19)		0.48
Type 2 diabetes	−0.07 (−0.22 to 0.08)		0.35
Vitamin D levels	0.23 (−0.52 to 0.98)		0.56

### Mendelian randomisation

**Fig 3:**
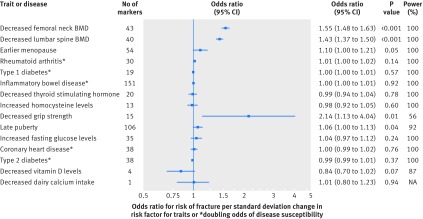
Forest plot showing effect of 15 genetically determined risk factors on fracture risk. Power=statistical power to detect an odds ratio of 1.15 at α≤3.3×10^−3^; NA=not applicable; BMD=bone mineral density.

Using mendelian randomisation analyses to assess the effect of the 15 risk factors on fracture, we saw evidence for a major effect of genetically decreased bone mineral density on fracture risk (fig 3 and table 4; odds ratio per standard deviation decrease in femoral neck bone mineral density=1.55, 95% confidence interval 1.48 to 1.63, P=1.5×10^−68^). We also observed a large effect of grip strength on fracture risk (2.14, 1.13 to 4.04, P=0.01), but these results had wide confidence intervals and were not significant after multiple testing correction. 

**Table 4:** Estimated effects of 15 genetically determined risk factors on fracture risk

Trait or disease	No of markers	Inverse variance weighted meta-analysis		Power (%)‡	Mendelian randomisation-Egger regression§	
		Odds ratio (95% CI)*	P		Intercept (95% CI)	P
Decreased femoral neck BMD¶	43	1.55 (1.48 to 1.63)	1.5×10^−68^	100	−0.0010 (−0.011 to 0.008)	0.83
Decreased lumbar spine BMD¶	40	1.43 (1.37 to 1.50)	2.3×10^−55^	100	0.0050 (−0.006 to 0.014)	0.93
Earlier menopause	54	1.10 (1.00 to 1.21)	0.05	100	0.0007 (−0.006 to 0.007)	0.83
Rheumatoid arthritis†	30	1.01 (1.00 to 1.02)	0.14	100	0.0099 (0.003 to 0.017)	0.005
Type 1 diabetes†	19	1.00 (1.00 to 1.01)	0.57	100	0.0028 (−0.004 to 0.010)	0.39
Inflammatory bowel disease†	151	1.00 (1.00 to 1.01)	0.92	100	0.0003 (−0.003 to 0.004)	0.86
Decreased thyroid stimulating hormone	20	0.99 (0.94 to 1.04)	0.78	100	0.0050 (−0.019 to 0.009)	0.47
Increased homocysteine levels	13	0.98 (0.92 to 1.05)	0.60	100	0.0134 (0.001 to 0.026)	0.03
Decreased grip strength	15	2.14 (1.13 to 4.04)	0.01	56	0.1070 (0.011 to 0.203)	0.03
Late puberty	106	1.06 (1.00 to 1.13)	0.04	92	0.0036 (−0.002 to 0.009)	0.21
Increased fasting glucose levels	35	1.04 (0.97 to 1.12)	0.24	100	−0.0083 (−0.014 to −0.002)	0.01
Coronary heart disease†	38	1.00 (0.99 to 1.02)	0.76	100	0.0028 (−0.007 to 0.013)	0.57
Type 2 diabetes†	38	0.99 (0.99 to 1.01)	0.37	100	−0.0089 (−0.016 to −0.002)	0.02
Decreased vitamin D levels	4	0.84 (0.70 to 1.02)	0.07	87	−0.0143 (−0.103 to 0.074)	0.56
Decreased dairy calcium intake	1	1.01 (0.80 to 1.23)	0.94	NA	NA	NA

NA=not applicable; BMD=bone mineral density.

* Odds ratio is for the risk of fracture per standard deviation change in the risk factor for traits (1 standard deviation change=0.13 g femoral neck BMD, 0.18 g lumbar spine BMD, 3.9 years earlier menopause, 0.76 mIU/L thyroid stimulating hormone, 11.3 kg grip strength, 1.42 years late puberty, 0.62 mmol/L fasting glucose, 25.2 nmol/L vitamin D), or †risk of fracture per doubling of odds of disease susceptibility; dairy calcium intake units are servings/day. Estimates obtained using a fixed effects model.

‡ Statistical power to detect an odds ratio of 1.15 at α≤3.3×10^−3^.

§ Egger regression analyses can be performed if the number of genetic variants is more than two; Egger effect estimates are presented in table S7.

¶ Findings that remain associated (that is, α<3.3×10^−3^) after correction for multiple testing.

Vitamin D levels assessed by use of 25-hydroxy-vitamin D variants were not found to be linearly associated with increased fracture risk (odds ratio per standard deviation decrease=0.84, 95% confidence interval 0.70 to 1.02, P=0.07). Most of these mendelian randomisation effects did not seem to be strongly influenced by directional pleiotropy, because the intercepts of the mendelian randomisation-Egger test were tightly centred around the null, except for rheumatoid arthritis, type 2 diabetes, grip strength, glucose, and homocysteine levels (table 4). The estimates from the inverse variance weighted fixed meta-analysis were very similar to the estimates from the weighted median and penalised weighted median method (table S8). However, despite some indication of causality of fasting glucose levels on fracture risk (table S8) in the median weighted analyses, it did not surpass the multiple testing threshold. 

Consistent with the results of the genetic correlation analyses, we found that none of the other evaluated clinical risk factors had evidence of a causal effect on risk of fracture, despite adequate statistical power (mean=98% (range 56-100%), table 4).^45^ When evaluating the effect of genetically increased risk factors on bone mineral density (table S9), only age of puberty had an effect on bone mineral density after accounting for multiple testing; fasting glucose, type 2 diabetes, and age at menopause had marginal effects, consistent with a recent mendelian randomisation study of type 2 diabetes and glycaemic traits on bone mineral density.^54^

We next undertook careful evaluation of the three mendelian randomisation assumptions. The first assumption was verified by the selection of only common variants (minor allele frequency >5%) strongly associated with the clinical risk factor (P<5×10^−8^). After performing a thorough literature search, we can exclude reported associations between the genetic variants and potential confounding factors (second assumption). Finally, by using mendelian randomisation-Egger regression, we found no evidence of the presence of pleiotropy between the instruments and the outcomes (table 4).

## Discussion

### Principal findings and interpretation

In this large GWAS for fracture, we identified genetic determinants (at 15 loci) of fracture and tested the role of 15 selected clinical risk factors on fracture risk and bone mineral density. Using mendelian randomisation analyses, we demonstrated that genetically decreased bone mineral density (and, to a lesser extent, hand grip strength) was the only clinical risk factor among those tested, with evidence for an effect on fracture risk. By contrast, despite high statistical power, none of the other tested and well accepted risk factors (eg, rheumatoid arthritis and other causes of secondary osteoporosis) or any of the other clinically relevant risk factors (vitamin D levels, dairy food derived calcium intake, fasting glucose, type 2 diabetes, and coronary heart disease) had evidence of a major causal effect on fracture risk. Furthermore, all identified genetic determinants of fracture also influenced bone mineral density.

In our previous work,^11^ we tested 96 bone mineral density markers for association with fracture. In the meta-analysis, 14 bone mineral density loci were associated with fracture risk (P<5×10^−4^), of which six surpassed genome wide significance (P<5×10^−8^). In our current project, we began with GWAS meta-analysis for fracture risk. We confirmed the 2p16.2 (*SPTBN1*), 7q21.3 (*SHFM1*), 10q21.1 (*MBL2*/*DKK1*), 11q13.2 (*LRP5*), and 18p11.21 (*FAM210A*) loci, and observed an increased signal at *SOST*, *CPED1*/*WNT16*, *FUPB3*, *DCDC5*, *RPS6KA5*, *STARD3NL*, and *CTNNB1*. Lastly, we added the 6q22.33 (*RSPO3*), 6q25.1 (*ESR1*), 7p12.1 (*GRB10*/*COBL*), and 21q22.2 (*ETS2*) loci to the list of novel fracture loci. Among the genome wide significant loci associated with fracture, several contain well established causal proteins for fracture risk that are targets for clinically useful osteoporotic fracture treatments, such as *ESR1*, which encodes the oestrogen receptor, and *SOST*, which encodes sclerostin.^55^ These discoveries highlight known and novel factors in pathways critical to bone biology (that is, Wnt, for mesenchymal stem cell differentiation) as well as potential new factors and biological pathways that might constitute future drug targets.^56^

All the discovered fracture loci are also associated with bone mineral density, implying that skeletal fragility characterised by reduced bone mineral density is central to the pathophysiology of osteoporotic fracture. This contention is in line with the significant genetic correlation we identified between bone mineral density and fracture. Our mendelian randomisation analyses also indicate that the suggestive effect of late puberty and earlier age at menopause on fracture risk is, at least partly, mediated through reduced bone mineral density. By contrast, hand grip strength was not found to be a determinant of bone mineral density, and vice versa. Still, grip strength could be a proxy for overall muscle strength and risk of falling, and could be involved in a pathway leading to fracture independently of bone mineral density.^5^ ^6^ However, the hand grip estimates holds wide confidence intervals in our analyses expressed in standard deviations to allow comparison to other risk factors. We believe that these large standard deviations can be attributed to multiple factors, including effort and encouragement of the participants, posture, position, and intrinsic measurement variability between individuals. A recent effort using UK Biobank data also showed through mendelian randomisation that higher grip strength is associated with decreased fracture risk.^18^ As such, inclusion of grip strength (or a different assessment of muscle function such as leg strength) could improve the predictive performance of risk prediction calculators that already contain bone mineral density, just as has been reported for a history of falls.^5^ ^6^

Older individuals at high risk of fractures often have low levels of vitamin D (owing to low dietary intake and sun exposure). Therefore, fracture prevention guidelines have suggested the use of vitamin D supplementation in the general population.^57^ ^58^ These recommendations have contributed to the marked increase in vitamin D use in older populations worldwide, where in the United States alone the proportion of individuals aged 70 years and older who use at least 1000 IU of vitamin D daily increased about 100-fold from 2000 to 2014.^59^ Despite these guideline recommendations, it is unclear whether modestly low levels of vitamin D, rather than profoundly low values, are causally associated with a higher risk of fracture. Our mendelian randomisation work examined a linear relation between vitamin D levels and fracture risk. We did not test for the possibility of a threshold dependent relation—that is, effects that could be present only at very low levels of vitamin D. Nevertheless, our analyses showed that vitamin D levels had no protective linear effect on fracture in community dwelling individuals, despite adequate statistical power. We also show, in line with other previous reports,^60^ ^61^ ^62^ no evidence for a causal effect of vitamin D levels on bone mineral density. Although a threshold effect is likely to be present, where profoundly lowered vitamin D levels do increase risk of fracture, our mendelian randomisation results strongly suggest that increasing levels of vitamin D in the (non-deficient) general population is unlikely to decrease risk of fracture.

Likewise, calcium supplementation has been called into question recently. A mendelian randomisation study found that higher levels of serum calcium are a risk factor for coronary heart disease,^63^ supporting the current recommendation of not exceeding total calcium intake of 1200 mg/day in older individuals.^64^ Further, our study assessed the lactose persistence variant as a surrogate of long term intake of dairy calcium (used previously as an instrument for dairy consumption in association with blood pressure^26^ and other traits), and found no evidence for a protective effect of sustained intake of dairy derived calcium on fracture risk.

### Comparison with other studies

Previous observational studies and clinical trials have reported the beneficial effect of vitamin D^65^ ^66^ or calcium^67^ supplementation on fracture risk reduction, findings which are not supported by our results. These discrepant findings can be due to inadequate methods or high heterogeneity induced, for example, by combining community dwelling participants and inpatients in the same analysis. Consistent with our findings, a recent meta-analysis of 33 randomised trials^68^ (n=51 145) found that supplementation with calcium, vitamin D, or both did not decrease the incidence of fractures in community dwelling older adults. Findings such as these should be interpreted with caution, because they do not necessarily apply to patients undergoing osteoporosis treatment, considering that trials evaluating osteoporosis treatment are carried out concomitant with vitamin D and calcium supplementation. 

Studies seeking to show whether these supplements do increase the efficacy of osteoporotic treatment or decrease adverse events (that is, hypocalcaemia) are lacking. In either case, screening for vitamin D deficiency and seeking its correction should be warranted before the initiation of anti-resorptive treatment. Moreover, in a recent mendelian randomisation study investigating the role of 25-hydroxy-vitamin D in maintaining bone mineral density,^62^ increased levels of 25-hydroxy-vitamin D had no effect on bone mineral density measured by dual energy x-ray absorptiometry (n=32 965; 0.02 g/cm^2^ change in femoral neck bone mineral density per standard deviation increase in 25-hydroxy-vitamin D). However, increased 25-hydroxy-vitamin D was associated with a slight reduction in heel bone mineral density estimated by ultrasonography (n=142 487; −0.03 g/cm^2^ change in estimated bone mineral density per standard deviation increase in 25-hydroxy-vitamin D). These results are consistent with our mendelian randomisation findings of no causal effect of vitamin D levels on fracture.

### Implications for clinicians

Our mendelian randomisation findings are relevant to clinical care. Although clinical risk factors, when used jointly in well validated prediction algorithms, predict fracture risk, our findings are a reminder that clinically relevant changes in most of these risk factors are unlikely to result in large differences in fracture risk. These findings also suggest clinical outcomes such as fracture risk can be subject to bias owing to uncontrolled confounding in observational epidemiological studies. A strength of our study design is that mendelian randomisation limits this potential confounding, because alleles are essentially assigned randomly at conception, and are therefore not generally affected by confounders. Furthermore, because allele assignment must precede fracture, mendelian randomisation is not prone to bias due to reverse causation. These findings provide guidance for the design of future clinical trials on interventions that are more likely to be successful in reducing fracture risk.

Epidemiological studies have shown that older people with a fracture will have abnormal bone mineral density in the osteoporotic range (that is, T score lower than −2.5 standard deviations), but most will have a fracture will be osteopenic (T score between −1 and −2.5 standard deviations). In fact, about 87% of women and 82% of men with a non-vertebral fracture have a T score lower than −1.0.^69^ Our findings suggest that low bone mineral density (not only after reaching the osteoporotic range) constitutes a risk factor that captures a substantial and causal part of the influences that increase risk for all types of fracture. Therefore, interventions targeting an increase in bone mineral density (presuming this is associated with improvements in bone structure or quality) are likely to have pivotal roles in reducing fracture risk.

The interpretation of our findings merits careful consideration for some of the risk factors. Hyperthyroidism is an established risk factor for fracture, and we have not used genetic determinants of hyperthyroidism risk, but rather genetic determinants of thyroid stimulating hormone level, which are likely to be different. Moreover, our study described the effect of clinical risk factors on fracture in the general population, and is therefore not generalisable to states or conditions of extreme circumstances known to cause fracture (eg, sustained vitamin D deficiency, rickets, or osteomalacia). Furthermore, our results apply only to 25-hydroxy-vitamin D, and might not necessarily reflect effects of its active form, 1,25-dihydroxy-vitamin D. However, vitamin D supplementation, as is commonly used, acts by influencing 25-hydroxy-vitamin D. Altogether, the course of action for effective fracture prevention relies on establishing vitamin D deficiency and seeking its correction, rather than the widespread use of non-indicated ineffective supplementation.

### Strengths and weaknesses of the study

To our knowledge, we have generated the largest and most comprehensive assessment of the genetic determinants of fracture risk so far. Moreover, use of the largest GWAS datasets available enabled adequate power to estimate the relation between genetically modified risk factors and fracture. Our study also had limitations. In our mendelian randomisation approach, we were unable to account for the sample overlap between the exposure and outcome GWAS datasets. However, we used powerful instruments to estimate the relation^39^ between the risk factors and the outcomes. Therefore, any sample overlap should not significantly bias our findings. Another potential limitation was that the first release of the UK Biobank selected some individuals based on a nested case-control study of smoking and lung function,^70^ and is therefore subject to selection bias.^71^ But after excluding the UK Biobank from our analyses, we observed no significant differences. Furthermore, the majority of our cohorts were imputed to HapMap (instead of more comprehensive reference panels), which could have affected the number of identified loci. However, given our power setting, our focus was mainly on common variants (which are well characterised in the HapMap imputation panel). In addition, analysis of large cohorts (UK Biobank and EPIC-Norfolk) imputed to more recent reference panels did not yield additional genome wide significant loci.

Moreover, we could not assess several relevant clinical risk factors. For example, body mass index^72^ ^73^ ^74^ could not be assessed in our mendelian randomisation framework because all GWAS analyses of fracture have been adjusted for body weight, preventing any inference assessment on causality. We also lacked power to estimate the casual relation between smoking and alcohol consumption: two potentially key risk factors for fracture. Similarly, we did not evaluate other risk factors that were not modifiable (such as age, sex, parental fracture history and body height), or those that have not been assessed by GWAS to yield genetic instruments for mendelian randomisation studies (such as falls, which are likely to be an important modifiable risk factor for fracture).^75^

Furthermore, factors unlikely to be predominantly genetic in origin (eg, occupation) might still have a role in the pathogenesis of fracture but could not be readily assessed through our mendelian randomisation approach. Nevertheless, proxy phenotypes for such risk factors are increasingly been investigated by GWAS (eg, education for occupation), and can be used as robust instruments in future research. In addition, because information on bone mineral density was not available for all study participants who were investigated for fracture, we could not determine directly the degree to which bone mineral density mediated the effect of genetic determinants on fracture risk. Mendelian randomisation is a helpful method to minimise several biases in observational studies, but the possibility of residual pleiotropy could bias estimates in this study. However, the likelihood of this bias is reduced because the mendelian randomisation-Egger regression test showed no clear directional pleiotropy for most of the factors. Lastly, because most of the study population was of European ancestry, results should not be directly generalised to other ethnicities.

Similarly, null results of a mendelian randomisation study could be influenced by canalisation, which is defined as compensatory feedback mechanisms that cannot be taken into account.^76^ The possible influence of the risk factors on fracture risk might be specifically linked to their complications or management of the disease, which we also could not take into account in mendelian randomisation. As in most epidemiological studies, mendelian randomisation also assumes a linear relation between the risk factor and the outcome, which might not invariably be the case for all risk factors of fracture. Some risk factors, such as vitamin D and estimated calcium intake, could have non-linear threshold associations, as discussed above. Furthermore, we could not account for the dose-response association (eg, between the lactose variant rs4988235 and dairy intake) within our design, or differences in biological effects across different types of grouped exposures (that is, fermented *v* non-fermented types of dairy products). Finally, the non-significant trend observed for vitamin D towards having increased risk of fracture could be attributed to the selection of healthy people (that is, participants with very low levels of vitamin D and fracture, as well as those who are older, frail, and physically impaired, could have been under-represented in the studies included in the GWAS meta-analyses). Therefore, the vitamin D estimates of the current study cannot be generalised to these groups of older people.

### Conclusion

From a study of over 500 000 individuals (about 185 000 fracture cases), we provide evidence that the main genetic determinants of osteoporotic fracture also influence bone mineral density, which was the only clinical risk factor to have shown a major effect on fracture risk among the study population assessed. By contrast, we found that other genetically estimated clinical risk factors for fracture, had either a very modest or no effect on fracture risk in the general population. Notably, genetic predisposition to lower levels of vitamin D and estimated calcium intake from dairy sources were not associated with fracture risk. Our study confirms bone mineral density as a pivotal cause of osteoporotic fracture and postulates that, among all the clinical risk factors we evaluated, interventions aimed at increasing bone mineral density are likely to have the most clinically relevant effect on fracture risk reduction.

**What is already known on this topic**

The genetic determinants of fracture risk are not well described, and whether commonly used clinical risk factors for fracture are causal is not known
For example, the effect of vitamin D supplementation in the general population on fracture risk is under debate; although such supplementation is part of clinical guidelines, recent randomised controlled trials have failed to consistently show a beneficial effect

**What this study adds**

This mendelian randomisation study provides evidence against a causal effect of several proposed clinical risk factors for fractures (eg, diabetes, glucose, rheumatoid arthritis, and vitamin D)
Genetic predisposition to lower vitamin D levels and estimated calcium intake from dairy sources were not associated with fracture risk
However, these results highlight the central causal role of low bone mineral density in the pathophysiology of fracture risk

## Web extra

Extra material supplied by authors

**Web appendix:** Supplementary note and full list of authorship details

## Notes

The following members of the GEFOS/GENOMOS consortium and the 23andMe research team are coauthors of this manuscript: Vincenzo Forgetta, Aaron Leong, Omar S Ahmad, Charles Laurin, Lauren E Mokry, Stephanie Ross, Cathy E Elks, Jack Bowden, Nicole M Warrington, Aaron Kleinman, Sara M Willems, Daniel Wright, Felix R Day, Anna Murray, Katherine S Ruth, Konstantinos K Tsilidis, Cheryl L Ackert-Bicknell, J H Duncan Bassett, Bram C J van der Eerden, Kaare M Gautvik, Sjur Reppe, Graham R Williams, Carolina Medina-Gómez, Karol Estrada, Najaf Amin, Joshua C Bis, Stephan Breda, Daniel I Chasman, Serkalem Demissie, Anke W Enneman, Yi-Hsiang Hsu, Thorvaldur Ingvarsson, Mika Kähönen, Candace Kammerer, Andrea Z Lacroix, Guo Li, Ching-Ti Liu, Yongmei Liu, Mattias Lorentzon, Reedik Mägi, Evelin Mihailov, Lili Mlani, Alireza Moayyeri, Carrie M Nielson, Nerea Alonso, Pack Chung Sham, Kristin Siggeirsdotir, Gunnar Sigurdsson, Unnur Thorsteinsdottir, Stella Trompet, Gudmar Thorleifsson, Liesbeth Vandenput, Nathalie van der Velde, Jorma Viikari, Su-Mei Xiao, Jing Hua Zhao, Kristina E Akesson, Marianne Andersen, Biljana Atanasovska, Susana Balcells, Joel Eriksson, Melissa M Formosa, Carmen Garcia-Ibarbia, Jesús Gonzalez-Macias, Natalia Garcia-Giralt, Goran Hallmans, Magnus Karlsson, Rita Khusainova, Beom-Jun Kim, Timothy C Y Kwok, Seung Hun Lee, Ping C Leung, Hans Mallmin, Laura Masi, Beatrice S Melin, Simona Mencej-Bedrac, Maria Nethander, José M Olmos, Panagoula Kollia, Janez Prezelj, Natasja M van Schoor, Olle Svensson, Pawel Szulc, Carmen Valero, Jean Woo, Maria Brandi, Sulin Cheng, Roland Chapurlat, Claus Christiansen, Cyrus Cooper, George Dedoussis, John A Eisman, Morten Frost, Sylvie Giroux, Daniel Grinberg, David Goltzman, Lynne J Hocking, Wim Van Hul, Jung-Min Koh, Lars Rejnmark, Jens-Erik B Jensen, Bente Langdahl, Joshua R Lewis, Roman S Lorenc, Elza Khusnutdinova,Janja Marc, Fiona E McGuigan, Dan Mellström, Karl Michaelsson, Xavier Nogues, Peter Nordström, Barbara Obermayer-Pietsch, Ulrika Pettersson-Kymmer, Richard L Prince, Jonathan Reeve, David M Reid, Jose A Riancho, Francois Rousseau, Nelson L S Tang, Angela Xuereb-Anastasi, William D Leslie, Daniel S Evans, Steven R Cummings, Jane Cauley, Cornelia M van Duijn, Matt Brown, Emma L Duncan, Lisette CPGM de Groot, Tonu Esko, Vilmundar Gudnason, Tamara B Harris, Rebecca D Jackson, J Wouter Jukema, M Arfan Ikram, David Karasik, Stephen Kaptoge, Kay-Tee Khaw, Annie Wai-Chee Kung, Terho Lehtimäki, Leo-Pekka Lyytikäinen, Paul Lips, Robert Luben, Andres Metspalu, Joyce B J van Meurs, Ryan L Minster, Eric Orwoll, Edwin Oei, Bruce M Psaty, Olli T Raitakari, Stuart H Ralston, Paul M Ridker, John A Robbins, Albert V Smith, Tim D Spector, Unnur Styrkarsdottir, Joseph Zmuda, Gregory J Tranah, Kari Stefansson, Andre G Uitterlinden, M Carola Zillikens, Evangelia E Ntzani, Evangelos Evangelou, John P A Ioannidis, John R B Perry, Joyce Y Tung, David A Hinds, Robert A Scott. Full details of the authorship, including job titles and affiliations, are included in the supplemental material.

23andMe research team: Michelle Agee, Babak Alipanahi, Adam Auton, Robert K Bell, Katarzyna Bryc, Sarah L Elson, Pierre Fontanillas, Nicholas A Furlotte, David A Hinds, Karen E Huber, Aaron Kleinman, Nadia K Litterman, Matthew H McIntyre, Joanna L Mountain, Elizabeth S Noblin, Carrie A M Northover, Steven J Pitts, J Fah Sathirapongsasuti, Olga V Sazonova, Janie F Shelton, Suyash Shringarpure, Chao Tian, Joyce Y Tung, Vladimir Vacic, and Catherine H Wilson.

GEFOS/GENOMOS consortium: Vincenzo Forgetta, Aaron Leong, Omar S Ahmad, Charles Laurin, Lauren E Mokry, Stephanie Ross, Cathy E Elks, Jack Bowden, Nicole M Warrington, Sara M Willems, Daniel Wright, Felix R Day, Anna Murray, Katherine S Ruth, Konstantinos K Tsilidis, Cheryl L Ackert-Bicknell, J H Duncan Bassett, Bram C J van der Eerden, Kaare M Gautvik, Sjur Reppe, Graham R Williams, Carolina Medina-Gómez, Karol Estrada, Najaf Amin, Joshua C Bis, Stephan Breda, Daniel I Chasman, Serkalem Demissie, Anke W Enneman, Yi-Hsiang Hsu, Thorvaldur Ingvarsson, Mika Kähönen, Candace Kammerer, Andrea Z LaCroix, Guo Li, Ching-Ti Liu, Yongmei Liu, Mattias Lorentzon, Reedik Mägi, Evelin Mihailov, Lili Mlani, Alireza Moayyeri, Carrie M Nielson, Nerea Alonso, Pack Chung Sham, Kristin Siggeirsdotir, Gunnar Sigurdsson, Unnur Thorsteinsdottir, Stella Trompet, Gudmar Thorleifsson, Liesbeth Vandenput, Nathalie van der Velde, Jorma Viikari, Su-Mei Xiao, Jing Hua Zhao, Kristina E Akesson, Marianne Andersen, Biljana Atanasovska, Susana Balcells, Joel Eriksson, Melissa M Formosa, Carmen Garcia-Ibarbia, Jesús Gonzalez-Macias, Natalia Garcia-Giralt, Goran Hallmans, Magnus Karlsson, Rita Khusainova, Beom-Jun Kim, Timothy C Y Kwok, Seung Hun Lee, Ping C Leung, Hans Mallmin, Laura Masi, Beatrice S Melin, Simona Mencej-Bedrac, Maria Nethander, José M Olmos, Panagoula Kollia, Janez Prezelj, Natasja M van Schoor, Olle Svensson, Pawel Szulc, Carmen Valero, Jean Woo, Maria Brandi, Sulin Cheng, Roland Chapurlat, Claus Christiansen, Cyrus Cooper, George Dedoussis, John A Eisman, Morten Frost, Sylvie Giroux, Daniel Grinberg, David Goltzman, Lynne J Hocking, Wim Van Hul, Jung-Min Koh, Lars Rejnmark, Jens-Erik B Jensen, Bente Langdahl, Joshua R Lewis, Roman S Lorenc, Elza Khusnutdinova, Janja Marc, Fiona E McGuigan, Dan Mellström, Karl Michaelsson, Xavier Nogues, Peter Nordström, Barbara Obermayer-Pietsch, Ulrika Pettersson-Kymmer, Richard L Prince, Jonathan Reeve, David M Reid, Jose A Riancho, Francois Rousseau, Nelson L S Tang, Angela Xuereb-Anastasi, William D Leslie, Daniel S Evans, Steven R Cummings, Jane Cauley, Cornelia M van Duijn, Matt Brown, Emma L Duncan, Lisette CPGM. de Groot, Tonu Esko, Vilmundar Gudnason, Tamara B Harris, Rebecca D Jackson, J Wouter Jukema, M Arfan Ikram, David Karasik, Stephen Kaptoge, Kay-Tee Khaw, Annie Wai-Chee Kung, Terho Lehtimäki, Leo-Pekka Lyytikäinen, Paul Lips, Robert Luben, Andres Metspalu, Joyce B J van Meurs, Ryan L Minster, Eric Orwoll, Edwin Oei, Bruce M Psaty, Olli T Raitakari, Stuart H Ralston, Paul M Ridker, John A Robbins, Albert V Smith, Tim D Spector, Unnur Styrkarsdottir, Joseph Zmuda, Gregory J Tranah, Kari Stefansson, Andre G Uitterlinden, M Carola Zillikens, Evangelia E Ntzani, Evangelos Evangelou, John P A Ioannidis, John R B Perry, and Robert A Scott.

Contributors: KT, JAM, LO, H-FZ, JBR, and FRi designed the study. KT, LO, H-FZ, VF, NMW, AK, NAm, JCB, SBr, SD, AWE, Y-HH, TI, CK, GL, C-TL, YL, RM, EM, LMi, AM, CMN, NAl, PCS, KSi, GS, ST, GT, LV, JV, S-MX, JHZ, MA, BA, JE, MMF, CG-I, JG-M, NG-G, GH, MKa, RK, B-JK, TCYK, SHL, PCL, HM, LMa, BSM, SMB, MN, JMO, JP, NvS, OS, CV, JW, SC, RC, CCh, MF, SG, LJH, WVH, J-MK, LR, J-EBJ, JRL, RSL, FEM, DM, XN, PN, JR, WDL, DSE, DK, SK, TL, L-PL, RL, JBJvM, RLM, EOe, PMR, JAR, AVS, US, JZ, GJT, MCZ, and DME were involved in study specific phenotyping and data analysis. DIC, MKä, AZL, ML, KSt, NvdV, KA, SBa, PK, PS, MBra, CCo, GD, JAE, DGr, DGo, BL, EK, JM, KM, BO-P, UP-K, RLP, DMR, JAR, FRo, NLST, AX-A, SRC, JC, CMvD, MBro, ELD, LdG, TE, VG, TBH, RDJ, JWJ, MAI, K-TK, AWCK, PL, AMe, EOr, BMP, OTR, SHR, TDS, UT, AGU, JYT, DAH, DPK, CO, JBR, and FRi were involved with study specific coordination and management. KT, JAM, KE, KKT, EEN, EE, and JPAI did the meta-analysis. CLA-B, JHDB, BCJvdE, KMG, SR, GRW, and CM-G did the functional work. KT, JAM, H-FZ, DME, DPK, CO, JBR, FRi, VF, AL, OSA, CL, LEM, SR, CEE, JB, SMW, DW, FRD, AM, KSR, JRBP, and RAS did the mendelian randomisation analysis. KT, JAM, JBR, and FRi wrote the first draft. KT, JAM, LO, H-FZ, DME, DPK, CO, JBR, and FRi wrote the final version of the paper. All authors reviewed the manuscript, added appropriate revisions, agreed to submission for publication, and approved the final version. KT, JAM, LO, and H-FZ (first) are equal contributing authors, and DME, DPK, CO, JBR, and FRi (senior) are equal contributing authors. FRi and JBR are the guarantors. The corresponding authors attest that all listed authors meet authorship criteria and that no others meeting the criteria have been omitted.

Ethical approval: The different cohorts had approvals from respective national ethical committees for medical research.

Data sharing: All data are available for unrestricted use through the GEFOS consortium and can be downloaded here: http://www.gefos.org/. We request that all use of such data be properly attributed through citation of this article.

The corresponding authors (JBR and FRi) affirm that the manuscript is an honest, accurate, and transparent account of the study being reported; that no important aspects of the study have been omitted; and that any discrepancies from the study as planned (and, if relevant, registered) have been explained.

## References

[1] Melorose J Perroy R Careas S World population prospects. United Nations, 2015, 10.1017/CBO9781107415324.004.

[2] Harvey N Dennison E Cooper C Osteoporosis: impact on health and economics [correction in: *Nat Rev Rheumatol* 2010;6:184]. Nat Rev Rheumatol 2010;6:99-105. 10.1038/nrrheum.2009.260 20125177

[3] Cooper C Melton LJ Magnitude and impact of osteoporosis and fractures. Academic Press, 1996.

[4] Kanis JA Hans D Cooper C Task Force of the FRAX Initiative Interpretation and use of FRAX in clinical practice. Osteoporos Int 2011;22:2395-411. 10.1007/s00198-011-1713-z 21779818

[5] Nguyen ND Frost SA Center JR Eisman JA Nguyen TV Development of a nomogram for individualizing hip fracture risk in men and women. Osteoporos Int 2007;18:1109-17. 10.1007/s00198-007-0362-8 17370100

[6] Nguyen ND Eisman JA Center JR Nguyen TV Risk factors for fracture in nonosteoporotic men and women. J Clin Endocrinol Metab 2007;92:955-62. 10.1210/jc.2006-1476 17164302

[7] Khosla S Shane E A crisis in the treatment of osteoporosis. J Bone Miner Res 2016;31:1485-7. 10.1002/jbmr.2888 27335158

[8] Andrew T Antioniades L Scurrah KJ Macgregor AJ Spector TD Risk of wrist fracture in women is heritable and is influenced by genes that are largely independent of those influencing BMD. J Bone Miner Res 2005;20:67-74. 10.1359/JBMR.041015 15619671

[9] Michaëlsson K Melhus H Ferm H Ahlbom A Pedersen NL Genetic liability to fractures in the elderly. Arch Intern Med 2005;165:1825-30. 10.1001/archinte.165.16.1825 16157825

[10] Smith GD Ebrahim S ‘Mendelian randomization’: can genetic epidemiology contribute to understanding environmental determinants of disease? Int J Epidemiol 2003;32:1-22. 10.1093/ije/dyg070 12689998

[11] Estrada K Styrkarsdottir U Evangelou E Genome-wide meta-analysis identifies 56 bone mineral density loci and reveals 14 loci associated with risk of fracture. Nat Genet 2012;44:491-501. 10.1038/ng.2249 22504420 PMC3338864

[12] Day FR Ruth KS Thompson DJ PRACTICAL consortium kConFab Investigators AOCS Investigators Generation Scotland EPIC-InterAct Consortium LifeLines Cohort Study Large-scale genomic analyses link reproductive aging to hypothalamic signaling, breast cancer susceptibility and BRCA1-mediated DNA repair. Nat Genet 2015;47:1294-303. 10.1038/ng.3412 26414677 PMC4661791

[13] Okada Y Wu D Trynka G RACI consortium GARNET consortium Genetics of rheumatoid arthritis contributes to biology and drug discovery. Nature 2014;506:376-81. 10.1038/nature12873 24390342 PMC3944098

[14] Jostins L Ripke S Weersma RK International IBD Genetics Consortium (IIBDGC) Host-microbe interactions have shaped the genetic architecture of inflammatory bowel disease. Nature 2012;491:119-24. 10.1038/nature11582 23128233 PMC3491803

[15] Bradfield JP Qu H-Q Wang K A genome-wide meta-analysis of six type 1 diabetes cohorts identifies multiple associated loci. PLoS Genet 2011;7:e1002293. 10.1371/journal.pgen.1002293 21980299 PMC3183083

[16] Porcu E Medici M Pistis G A meta-analysis of thyroid-related traits reveals novel loci and gender-specific differences in the regulation of thyroid function. PLoS Genet 2013;9:e1003266. 10.1371/journal.pgen.1003266 23408906 PMC3567175

[17] van Meurs JBJ Dhonukshe-Rutten RAM Pluijm SMF Homocysteine levels and the risk of osteoporotic fracture. N Engl J Med 2004;350:2033-41. 10.1056/NEJMoa032546 15141041

[18] Willems SM Wright DJ Day FR GEFOS Any-Type of Fracture Consortium Large-scale GWAS identifies multiple loci for hand grip strength providing biological insights into muscular fitness. Nat Commun 2017;8:16015. 10.1038/ncomms16015 29313844 PMC5510175

[19] Perry JRB Day F Elks CE Australian Ovarian Cancer Study GENICA Network kConFab LifeLines Cohort Study InterAct Consortium Early Growth Genetics (EGG) Consortium Parent-of-origin-specific allelic associations among 106 genomic loci for age at menarche. Nature 2014;514:92-7. 10.1038/nature13545 25231870 PMC4185210

[20] Scott RA Lagou V Welch RP DIAbetes Genetics Replication and Meta-analysis (DIAGRAM) Consortium Large-scale association analyses identify new loci influencing glycemic traits and provide insight into the underlying biological pathways. Nat Genet 2012;44:991-1005. 10.1038/ng.2385 22885924 PMC3433394

[21] Wessel J Chu AY Willems SM EPIC-InterAct Consortium Low-frequency and rare exome chip variants associate with fasting glucose and type 2 diabetes susceptibility. Nat Commun 2015;6:5897. 10.1038/ncomms6897 25631608 PMC4311266

[22] Deloukas P Kanoni S Willenborg C CARDIoGRAMplusC4D Consortium DIAGRAM Consortium CARDIOGENICS Consortium MuTHER Consortium Wellcome Trust Case Control Consortium Large-scale association analysis identifies new risk loci for coronary artery disease. Nat Genet 2013;45:25-33. 10.1038/ng.2480 23202125 PMC3679547

[23] Morris AP Voight BF Teslovich TM Wellcome Trust Case Control Consortium Meta-Analyses of Glucose and Insulin-related traits Consortium (MAGIC) Investigators Genetic Investigation of ANthropometric Traits (GIANT) Consortium Asian Genetic Epidemiology Network–Type 2 Diabetes (AGEN-T2D) Consortium South Asian Type 2 Diabetes (SAT2D) Consortium DIAbetes Genetics Replication And Meta-analysis (DIAGRAM) Consortium Large-scale association analysis provides insights into the genetic architecture and pathophysiology of type 2 diabetes. Nat Genet 2012;44:981-90. 10.1038/ng.2383 22885922 PMC3442244

[24] Mokry LE Ross S Ahmad OS Vitamin D and risk of multiple sclerosis: a Mendelian randomization study [correction in: *PLoS Med* 2016;13:e1001981]. PLoS Med 2015;12:e1001866. 10.1371/journal.pmed.1001866 26305103 PMC4549308

[25] Wang TJ Zhang F Richards JB Common genetic determinants of vitamin D insufficiency: a genome-wide association study. Lancet 2010;376:180-8. 10.1016/S0140-6736(10)60588-0 20541252 PMC3086761

[26] Ding M Huang T Bergholdt HK Nordestgaard BG Ellervik C Qi L CHARGE Consortium Dairy consumption, systolic blood pressure, and risk of hypertension: Mendelian randomization study [correction in: *BMJ* 2017;358:j3550]. BMJ 2017;356:j1000. 10.1136/bmj.j1000 28302601 PMC6168037

[27] Zheng HF Forgetta V Hsu Y-H AOGC Consortium UK10K Consortium Whole-genome sequencing identifies EN1 as a determinant of bone density and fracture. Nature 2015;526:112-7. 10.1038/nature14878 26367794 PMC4755714

[28] Mackey DC Lui L-Y Cawthon PM Study of Osteoporotic Fractures (SOF) and Osteoporotic Fractures in Men Study (MrOS) Research Groups High-trauma fractures and low bone mineral density in older women and men. JAMA 2007;298:2381-8. 10.1001/jama.298.20.2381 18042915

[29] Sanders KM Pasco JA Ugoni AM The exclusion of high trauma fractures may underestimate the prevalence of bone fragility fractures in the community: the Geelong Osteoporosis Study. J Bone Miner Res 1998;13:1337-42. 10.1359/jbmr.1998.13.8.1337 9718203

[30] Enattah NS Sahi T Savilahti E Terwilliger JD Peltonen L Järvelä I Identification of a variant associated with adult-type hypolactasia. Nat Genet 2002;30:233-7. 10.1038/ng826 11788828

[31] Koek WNH van Meurs JB van der Eerden BC The T-13910C polymorphism in the lactase phlorizin hydrolase gene is associated with differences in serum calcium levels and calcium intake. J Bone Miner Res 2010;25:1980-7. 10.1002/jbmr.83 20225268

[32] Frank J Cichon S Treutlein J Genome-wide significant association between alcohol dependence and a variant in the ADH gene cluster. Addict Biol 2012;17:171-80. 10.1111/j.1369-1600.2011.00395.x 22004471 PMC3245349

[33] Gelernter J Kranzler HR Sherva R Genome-wide association study of alcohol dependence:significant findings in African- and European-Americans including novel risk loci. Mol Psychiatry 2014;19:41-9. 10.1038/mp.2013.145 24166409 PMC4165335

[34] Taylor AE Fluharty ME Bjørngaard JH Investigating the possible causal association of smoking with depression and anxiety using Mendelian randomisation meta-analysis: the CARTA consortium. BMJ Open 2014;4:e006141. 10.1136/bmjopen-2014-006141 25293386 PMC4187451

[35] O’Seaghdha CM Wu H Yang Q SUNLIGHT Consortium GEFOS Consortium Meta-analysis of genome-wide association studies identifies six new Loci for serum calcium concentrations. PLoS Genet 2013;9:e1003796. 10.1371/journal.pgen.1003796 24068962 PMC3778004

[36] Locke AE Kahali B Berndt SI LifeLines Cohort Study ADIPOGen Consortium AGEN-BMI Working Group CARDIOGRAMplusC4D Consortium CKDGen Consortium GLGC ICBP MAGIC Investigators MuTHER Consortium MIGen Consortium PAGE Consortium ReproGen Consortium GENIE Consortium International Endogene Consortium Genetic studies of body mass index yield new insights for obesity biology. Nature 2015;518:197-206. 10.1038/nature14177 25673413 PMC4382211

[37] Bulik-Sullivan BK Loh P-R Finucane HK Schizophrenia Working Group of the Psychiatric Genomics Consortium LD Score regression distinguishes confounding from polygenicity in genome-wide association studies. Nat Genet 2015;47:291-5. 10.1038/ng.3211 25642630 PMC4495769

[38] Burgess S Butterworth A Thompson SG Mendelian randomization analysis with multiple genetic variants using summarized data. Genet Epidemiol 2013;37:658-65. 10.1002/gepi.21758 24114802 PMC4377079

[39] Pierce BL Burgess S Efficient design for Mendelian randomization studies: subsample and 2-sample instrumental variable estimators. Am J Epidemiol 2013;178:1177-84. 10.1093/aje/kwt084 23863760 PMC3783091

[40] Mokry LE Ross S Ahmad OS Vitamin D and risk of multiple sclerosis: a Mendelian randomization study [correction in: *PLoS Med* 2016;13:e1001981]. PLoS Med 2015;12:e1001866. 10.1371/journal.pmed.1001866 26305103 PMC4549308

[41] Dastani Z Hivert M-F Timpson N DIAGRAM+ Consortium MAGIC Consortium GLGC Investigators MuTHER Consortium DIAGRAM Consortium GIANT Consortium Global B Pgen Consortium Procardis Consortium MAGIC investigators GLGC Consortium Novel loci for adiponectin levels and their influence on type 2 diabetes and metabolic traits: a multi-ethnic meta-analysis of 45,891 individuals. PLoS Genet 2012;8:e1002607. 10.1371/journal.pgen.1002607 22479202 PMC3315470

[42] Johnson T. Efficient calculation for multi-SNP genetic risk scores. 2012; 2012.

[43] Bowden J Davey Smith G Burgess S Mendelian randomization with invalid instruments: effect estimation and bias detection through Egger regression. Int J Epidemiol 2015;44:512-25. 10.1093/ije/dyv080 26050253 PMC4469799

[44] Gage SH Jones HJ Burgess S Assessing causality in associations between cannabis use and schizophrenia risk: a two-sample Mendelian randomization study. Psychol Med 2017;47:971-80. 10.1017/S0033291716003172 27928975 PMC5341491

[45] Brion M-JA Shakhbazov K Visscher PM Calculating statistical power in Mendelian randomization studies. Int J Epidemiol 2013;42:1497-501. 10.1093/ije/dyt179 24159078 PMC3807619

[46] Richards JB Rivadeneira F Inouye M Bone mineral density, osteoporosis, and osteoporotic fractures: a genome-wide association study. Lancet 2008;371:1505-12. 10.1016/S0140-6736(08)60599-1 18455228 PMC2679414

[47] Rivadeneira F Styrkársdottir U Estrada K Genetic Factors for Osteoporosis (GEFOS) Consortium Twenty bone-mineral-density loci identified by large-scale meta-analysis of genome-wide association studies. Nat Genet 2009;41:1199-206. 10.1038/ng.446 19801982 PMC2783489

[48] Styrkarsdottir U Halldorsson BV Gretarsdottir S New sequence variants associated with bone mineral density. Nat Genet 2009;41:15-7. 10.1038/ng.284 19079262

[49] Styrkarsdottir U Halldorsson BV Gretarsdottir S Multiple genetic loci for bone mineral density and fractures. N Engl J Med 2008;358:2355-65. 10.1056/NEJMoa0801197 18445777

[50] Duncan EL Danoy P Kemp JP Genome-wide association study using extreme truncate selection identifies novel genes affecting bone mineral density and fracture risk. PLoS Genet 2011;7:e1001372. 10.1371/journal.pgen.1001372 21533022 PMC3080863

[51] Zheng H-F Tobias JH Duncan E WNT16 influences bone mineral density, cortical bone thickness, bone strength, and osteoporotic fracture risk. PLoS Genet 2012;8:e1002745. 10.1371/journal.pgen.1002745 22792071 PMC3390364

[52] Medina-Gomez C Kemp JP Estrada K Meta-analysis of genome-wide scans for total body BMD in children and adults reveals allelic heterogeneity and age-specific effects at the WNT16 locus. PLoS Genet 2012;8:e1002718. 10.1371/journal.pgen.1002718 22792070 PMC3390371

[53] Visscher PM Brown MA McCarthy MI Yang J Five years of GWAS discovery. Am J Hum Genet 2012;90:7-24. 10.1016/j.ajhg.2011.11.029 22243964 PMC3257326

[54] Ahmad OS Leong A Miller JA A Mendelian randomization study of the effect of type-2 diabetes and glycemic traits on bone mineral density. J Bone Miner Res 2017;32:1072-81. 10.1002/jbmr.3063. 27982478

[55] Richards JB Zheng H-F Spector TD Genetics of osteoporosis from genome-wide association studies: advances and challenges [correction in: *Nat Rev Genet* 2012;13:672]. Nat Rev Genet 2012;13:576-88. 10.1038/nrg3228 22805710

[56] Hurle MR Nelson MR Agarwal P Cardon LR Trial watch: Impact of genetically supported target selection on R&D productivity. Nat Rev Drug Discov 2016;15:596-7. 10.1038/nrd.2016.164 27573226

[57] Papaioannou A Morin S Cheung AM Scientific Advisory Council of Osteoporosis Canada 2010 clinical practice guidelines for the diagnosis and management of osteoporosis in Canada: summary. CMAJ 2010;182:1864-73. 10.1503/cmaj.100771 20940232 PMC2988535

[58] Kanis JA McCloskey EV Johansson H Cooper C Rizzoli R Reginster JY Scientific Advisory Board of the European Society for Clinical and Economic Aspects of Osteoporosis and Osteoarthritis (ESCEO) and the Committee of Scientific Advisors of the International Osteoporosis Foundation (IOF) European guidance for the diagnosis and management of osteoporosis in postmenopausal women. Osteoporos Int 2013;24:23-57. 10.1007/s00198-012-2074-y 23079689 PMC3587294

[59] Rooney MR Harnack L Michos ED Ogilvie RP Sempos CT Lutsey PL Trends in use of high-dose vitamin D supplements exceeding 1000 or 4000 international units daily, 1999-2014. JAMA 2017;317:2448-50. 10.1001/jama.2017.4392 28632857 PMC5587346

[60] Leong A Rehman W Dastani Z METASTROKE The causal effect of vitamin D binding protein (DBP) levels on calcemic and cardiometabolic diseases: a Mendelian randomization study. PLoS Med 2014;11:e1001751. 10.1371/journal.pmed.1001751 25350643 PMC4211663

[61] Li S-S Gao L-H Zhang X-Y Genetically low vitamin D levels, bone mineral density, and bone metabolism markers: a Mendelian randomisation study. Sci Rep 2016;6:33202. 10.1038/srep33202 27625044 PMC5021966

[62] Larsson SC Melhus H Michaëlsson K Circulating serum 25-hydroxyvitamin D levels and bone mineral density: Mendelian randomization study. J Bone Miner Res 2018;33:840-4. 10.1002/jbmr.3389. 29338102

[63] Larsson SC Burgess S Michaëlsson K Association of genetic variants related to serum calcium levels with coronary artery disease and myocardial infarction. JAMA 2017;318:371-80. 10.1001/jama.2017.8981 28742912 PMC5817597

[64] Bauer DC Clinical practice. Calcium supplements and fracture prevention. N Engl J Med 2013;369:1537-43. 10.1056/NEJMcp1210380 24131178 PMC4038300

[65] Bergman GJD Fan T McFetridge JT Sen SS Efficacy of vitamin D_3_ supplementation in preventing fractures in elderly women: a meta-analysis. Curr Med Res Opin 2010;26:1193-201. 10.1185/03007991003659814 20302551

[66] Bischoff-Ferrari HA Willett WC Orav EJ A pooled analysis of vitamin D dose requirements for fracture prevention [correction in: *N Engl J Med* 2012;367:481]. N Engl J Med 2012;367:40-9. 10.1056/NEJMoa1109617 22762317

[67] Tang BM Eslick GD Nowson C Smith C Bensoussan A Use of calcium or calcium in combination with vitamin D supplementation to prevent fractures and bone loss in people aged 50 years and older: a meta-analysis. Lancet 2007;370:657-66. 10.1016/S0140-6736(07)61342-7 17720017

[68] Zhao J-G Zeng X-T Wang J Liu L Association between calcium or vitamin D supplementation and fracture incidence in community-dwelling older adults: a systematic review and meta-analysis. JAMA 2017;318:2466-82. 10.1001/jama.2017.19344 29279934 PMC5820727

[69] Schuit SCE van der Klift M Weel AEAM Fracture incidence and association with bone mineral density in elderly men and women: the Rotterdam Study. Bone 2004;34:195-202. 10.1016/j.bone.2003.10.001 14751578

[70] Wain LV Shrine N Miller S UK Brain Expression Consortium (UKBEC) OxGSK Consortium Novel insights into the genetics of smoking behaviour, lung function, and chronic obstructive pulmonary disease (UK BiLEVE): a genetic association study in UK Biobank. Lancet Respir Med 2015;3:769-81. 10.1016/S2213-2600(15)00283-0 26423011 PMC4593935

[71] Munafò MR Tilling K Taylor AE Evans DM Davey Smith G Collider scope: when selection bias can substantially influence observed associations. Int J Epidemiol 2018;47:226-35. 10.1093/ije/dyx206 29040562 PMC5837306

[72] De Laet C Kanis JA Odén A Body mass index as a predictor of fracture risk: a meta-analysis. Osteoporos Int 2005;16:1330-8. 10.1007/s00198-005-1863-y 15928804

[73] Johansson H Kanis JA Odén A A meta-analysis of the association of fracture risk and body mass index in women. J Bone Miner Res 2014;29:223-33. 10.1002/jbmr.2017 23775829

[74] Compston JE Flahive J Hosmer DW GLOW Investigators Relationship of weight, height, and body mass index with fracture risk at different sites in postmenopausal women: the Global Longitudinal study of Osteoporosis in Women (GLOW). J Bone Miner Res 2014;29:487-93. 10.1002/jbmr.2051 23873741 PMC4878680

[75] El-Khoury F Cassou B Charles M-A Dargent-Molina P The effect of fall prevention exercise programmes on fall induced injuries in community dwelling older adults: systematic review and meta-analysis of randomised controlled trials. BMJ 2013;347:f6234. 24169944 10.1136/bmj.f6234 PMC3812467

[76] Lawlor DA Harbord RM Sterne JAC Timpson N Davey Smith G Mendelian randomization: using genes as instruments for making causal inferences in epidemiology. Stat Med 2008;27:1133-63. 10.1002/sim.3034 17886233

