# Supplementary material for: Assessment of the genetic and clinical determinants of fracture risk: genome wide association and mendelian randomisation study
Source: BMJ. 2018 Aug 29;362:k3225. doi: 10.1136/bmj.k3225 (PMC6113773; doi:10.1136/bmj.k3225)
Supplement: Supplementary file 1 — Web appendix: Supplementary note and full list of authorship details [file trak043508.ww.pdf]

SUPPLEMENTARY NOTE

CONTENT

I. Full authorship details

II. Supplementary tables

III. Supplementary figures

IV. Full acknowledgements

I. Full authorship details

Assessment of the genetic and clinical determinants of fracture risk: a Mendelian randomization approach

Katerina Trajanoska PhD student[1,2]*, John A. Morris PhD student[3,4]*, Ling Oei assistant professor[1,2]*, Hou-Feng Zheng professor [5,6]*, David M. Evans professor[7,8]**, Douglas P. Kiel professor[9,10]**, Claes Ohlsson professor[11]**, J. Brent Richards professor[3,4]** and Fernando Rivadeneira associate professor[1,2]
** on behalf of GEFOS/GENOMOS consortium and the 23andMe research team

[1] Department of Internal Medicine, Erasmus MC, University Medical Center, Rotterdam, The Netherlands.
[2] Department of Epidemiology, Erasmus MC, University Medical Center, Rotterdam, The Netherlands.
[3] Lady Davis Institute, Jewish General Hospital, McGill University, Montréal, Québec, Canada.
[4] Department of Human Genetics, McGill University, Montréal, Québec, Canada.
[5] Institute of Basic Medical Sciences, DaP Lab, School of Life Sciences, Westlake University and Westlake Institute for Advanced Study, Westlake University, Hangzhou, Zhejiang, China.
[6] Institute of Aging Research and the Affiliated Hospital, School of Medicine, Hangzhou Normal University, Hangzhou, Zhejiang, China.
[7] MRC Integrative Epidemiology Unit, University of Bristol, Bristol, UK.
[8] The University of Queensland Diamantina Institute, The University of Queensland, Translational Research Institute, Brisbane, Australia.
[9] Institute for Aging Research, Hebrew SeniorLife, Harvard Medical School, Boston, Massachusetts, USA
[10] Department of Medicine, Beth Israel Deaconess Medical Center and Harvard Medical School, Boston, MA, USA
[11] Centre for Bone and Arthritis Research, Department of Internal Medicine, Institute of Medicine, Sahlgrenska, Gothenburg, Sweden

*These authors contributed equally
**These authors jointly supervised this work

Full details of coauthors and affiliations listed in the endmatter:

The following members of GEFOS/GENOMOS consortium and the 23andMe team are co-authors of this manuscript: Vincenzo Forgetta research associate[1], Aaron Leong endocrinologist[2,3], Omar S. Ahmad scientist[4], Charles Laurin postdoctoral researcher[5], Lauren E. Mokry scientist[1], Stephanie Ross scientist[6], Cathy E. Elks scientist[5,7], Jack Bowden programme leader[5], Nicole M. Warrington NHMRC early career researcher[8], Aaron Kleinman senior scientist[9], Sara M. Willems scientist[10], Daniel Wright PhD student[10], Felix R. Day career development fellow[10], Anna Murray associate professor[11], Katherine S. Ruth research fellow[11], Konstantinos K. Tsilidis assistant professor[12,13], Cheryl L. Ackert-Bicknell associate professor[14], J.H. Duncan Bassett professor[15], Bram C.J. van der Eerden assistant professor[16], Kaare M. Gautvik medical consultant[18,19], Sjur Reppe senior researcher[18,20], Graham R. Williams professor[15], Carolina Medina-Gómez postdoctoral researcher[17,21], Karol Estrada scientific researcher[17], Najaf Amin associate professor[21], Joshua C. Bis scientific researcher[22], Stephan Breda PhD student[23], Daniel I. Chasman associate professor[24,25], Serkalem Demissie associate professor[26], Anke W. Enneman scientist[17], Yi-Hsiang Hsu associate scientist[27,28,29], Thorvaldur Ingvarsson associate professor[30,31], Mika Kähönen professor [32,33], Candace Kammerer associate professor[34], Andrea Z. LaCroix professor[35], Guo Li scientist[22], Ching-Ti Liu associate professor[26], Yongmei Liu researcher[36], Mattias Lorentzon professor[37], Reedik Mägi senior scientist[38], Evelin Mihailov scientist[38], Lili Mlani research professor[38], Alireza Moayyeri honorary research associate[39], Carrie M. Nielson associate professor[40], Nerea Alonso postdoctoral researcher[41], Pack Chung Sham professor[42], Kristin Siggeirsdotir managing director of development[43], Gunnar Sigurdsson proffesor[44,45], Unnur Thorsteinsdottir vice president of research[45,46], Stella Trompet assistant professor[47,48], Gudmar Thorleifsson researcher[46], Liesbeth Vandenput associate professor[49], Nathalie van der Velde geriatrician consultant[17,50], Jorma Viikari professor[51,52], Su-Mei Xiao

scientist[53], Jing Hua Zhao senior investigator scientist[10], Kristina E. Akesson professor[54], Marianne Andersen professor[55], Biljana Atanasovska PhD student[56], Susana Balcells professor[57], Joel Eriksson senior lecturer[49], Melissa M. Formosa postdoctoral researcher[58], Carmen Garcia-Ibarbia medical doctor[59,60], Jesús Gonzalez-Macias medical doctor[59,60], Natalia Garcia-Giralt postdoctoral researcher[61], Goran Hallmans professor[62], Magnus Karlsson professor[54,63], Rita Khusainova researcher [64], Beom-Jun Kim associate professor[65], Timothy C.Y. Kwok professor[66], Seung Hun Lee associate professor[65], Ping C. Leung researcher[67], Hans Mallmin adjunct professor[68], Laura Masi senior researcher[69,70], Beatrice S. Melin professor[63], Simona Mencej-Bedrac HTA and pricing expert[71], Maria Nethander statistician[49,72], José M. Olmos assistant professor [59,60], Panagoula Kollia associate professor[73], Janez Prezelj retired[74], Natasja M. van Schoor assistant professor[75], Olle Svensson professor emeritus[76], Pawel Szulc senior researcher[77], Carmen Valero medical doctor[59,60], Jean Woo professor[66], Maria Brandi professor[78], Sulin Cheng professor[79], Roland Chapurlat professor[77], Claus Christiansen chairman[80], Cyrus Cooper professor[81], George Dedoussis professor[82], John A. Eisman professor[83], Morten Frost senior scientist[55], Sylvie Giroux research assistant [84], Daniel Grinberg professor[57], David Goltzman professor[85], Lynne J. Hocking senior scientist[86], Wim Van Hul professor[87], Jung-Min Koh professor[65], Lars Rejnmark professor[88], Jens-Erik B. Jensen head of department[88], Bente Langdahl professor[88], Joshua R. Lewis national health and medical research council career development fellow[89,90,91], Roman S. Lorenc retired[92], Elza Khusnutdinova professor[64,93], Janja Marc professor[71], Fiona E. McGuigan associate professor [54], Dan Mellström professor[49], Karl Michaelsson professor[68], Xavier Nogues head of department[61], Peter Nordström professor[94], Barbara Obermayer-Pietsch professor[95], Ulrika Pettersson-Kymmer associate professor[96], Richard L. Prince professor[90,97], Jonathan Reeve honorary senior research fellow[98], David M. Reid professor[86], Jose A. Riancho director[59,60], Francois Rousseau professor[84], Nelson L.S. Tang professor[99], Angela Xuereb-Anastasi professor[58], William D. Leslie professor[100], Daniel S. Evans scientist[101], Steven R. Cummings professor[101], Jane Cauley professor[102], Cornelia M van Duijn professor[21], Matt Brown professor[103], Emma L. Duncan professor[103,104] Lisette CPGM. de Groot professor[105], Tonu Esko deputy director of research[38,106], Vilmundar Gudnason professor[43,45], Tamara B. Harris chief of the interdisciplinary studies[107], Rebecca D. Jackson professor[108], J. Wouter Jukema professor[47], M. Arfan Ikram professor[21], David Karasik professor[27,109], Stephen Kaptoge senior statistician[39], Kay-Tee Khaw professor[39,110], Annie Wai-Chee Kung professor[53], Terho Lehtimäki professor[111,112], Leo-Pekka Lyytikäinen researcher[111,112], Paul Lips professor[113], Robert Luben[39], Andres Metspalu professor[38,114], Joyce B. J. van Meurs associate professor[17], Ryan L. Minster assistant professor[34], Eric Orwoll professor[115], Edwin Oei radiologist[23], Bruce M. Psaty professor[116],[117], Olli T. Raitakari professor[118,119], Stuart H. Ralston professor[41], Paul M. Ridker professor[24,25], John A. Robbins professor[120], Albert V. Smith senior researcher[43,45], Tim D. Spector professor[121], Unnur Styrkarsdottir senior scientist[48], Joseph Zmuda associate professor[102], Gregory J. Tranah senior scientist[101], Kari Stefansson CEO[44,46], Andre G. Uitterlinden professor[17,21], M. Carola Zillikens associate professor[17], Evangelia E. Ntzani associate professor[12,122], Evangelos Evangelou associate professor[12,13], John PA. Ioannidis professor[123], John R.B. Perry programme leader track[10], Joyce Y. Tung vice president of research[9], David A. Hinds research fellow[9], Robert A Scott senior scientist[10]

[1] Centre for Clinical Epidemiology, Lady Davis Institute, Jewish General Hospital, McGill University, Montréal, Québec, Canada.
[2] Division of General Internal Medicine, Massachusetts General Hospital, Boston, Massachusetts, USA.
[3] Department of Medicine, Harvard Medical School, Boston, Massachusetts, USA.
[4] Department of Medicine, McGill University Montréal, Québec, Canada.
[5] MRC Integrative Epidemiology Unit, University of Bristol, Bristol, UK.
[6] Departments of Clinical Epidemiology and Biostatistics, McMaster University, Hamilton, Ontario, Canada.
[7] Personalised Healthcare & Biomarkers, Innovative Medicines and Early Development Biotech Unit, AstraZeneca, Cambridge.
[8] The University of Queensland Diamantina Institute, The University of Queensland, Translational Research Institute, Brisbane, Australia.
[9] Department of Research, 23andMe, Mountain View, California, USA.
[10] MRC Epidemiology Unit, University of Cambridge School of Clinical Medicine, Cambridge, UK.
[11] Genetics of Complex Traits, University of Exeter Medical School, University of Exeter, Exter, UK.

[12] Department of Hygiene and Epidemiology, University of Ioannina Medical School, Ioannina, Greece.
[13] Department of Epidemiology and Biostatistics, Imperial College London, Faculty of Medicine, School of Public Health, London, UK.
[14] Center for Musculoskeletal Research, Department of Orthopaedics, University of Rochester, Rochester, New York, USA.
[15] Molecular Endocrinology Laboratory, Department of Medicine, Imperial College London, London, UK .
[16] Department of Internal Medicine, Laboratory for Calcium and Bone Metabolism, Erasmus MC, University Medical Center, Rotterdam, The Netherlands.
[17] Department of Internal Medicine, Erasmus MC, University Medical Center, Rotterdam, The Netherlands.
[18] Unger-Vetlesen Institute, Lovisenberg Diakonale Hospital, Oslo, Norway.
[19] Institute of Basic Medical Sciences, University of Oslo, Oslo, Norway.
[20] Department of Medical Biochemistry, Oslo University Hospital, Oslo, Norway.
[21] Department of Epidemiology, Erasmus MC, University Medical Center, Rotterdam, The Netherlands.
[22] Cardiovascular Health Research Unit, Department of Medicine, University of Washington, Seattle, Washington, USA.
[23] Department of Radiology and Nuclear Medicine, Erasmus MC,University Medical Center, Rotterdam, The Netherlands.
[24] Division of Preventive Medicine, Brigham and Women's Hospital, Boston, Massachusetts, USA.
[25] Harvard Medical School, Boston, Massachusetts, USA.
[26] Department of Biostatistics, Boston University School of Public Health, Boston, Massachusetts, USA.
[27] Institute for Aging Research, Hebrew SeniorLife, Harvard Medical School, Boston, Massachusetts, USA.
[28] BROAD Institute of MIT and Harvard, Cambridge, MA, USA.
[29] Molecular and Integrative Physiological Sciences, Harvard School of Public Health, Boston, Massachusetts, USA.
[30] Department of Orthopedic Surgery, Akureyri Hospital, Akureyri, Iceland.
[31] Institution of Health Science, University of Akureyri, Akureyri, Iceland.
[32] Department of Clinical Physiology, Tampere University Hospital, Tampere, Finland.
[33] Department of Clinical Physiology, Finnish Cardiovascular Research Center - Tampere, Faculty of Medicine and Life Sciences, University of Tampere, Tampere, Finland.
[34] Department of Human Genetic, Graduate School of Public Health, University of Pittsburgh, Pittsburgh, Pennsylvania, USA.
[35] Department of Family Medicine and Public Health, University of California – San Diego, SanDiego, California, USA.
[36] Department of Epidemiology and Prevention, Division of Public Health Sciences, Wake Forest University, Winston-Salem, North Carolina, USA.
[37] Geriatric Medicine, Department of Internal Medicine and Clinical Nutrition Institute of Medicine, University of Gothenburg, Mölndal, Sweden.
[38] Estonian Genome Center, University of Tartu, Tartu, Estonia.
[39] Department of Public Health and Primary Care, University of Cambridge, Cambridge, UK.
[40] School of Public Health, Oregon Health & Science University, Portland, Oregon, USA.
[41] Institute of Genetics and Molecular Medicine, University of Edinburgh, Edinburgh, UK.
[42] Department of Psychiatry, The University of Hong Kong, Hong Kong, China.
[43] Icelandic Heart Association and University of Iceland, Kopavogur , Iceland.
[44] Department of Endocrinology and Metabolism, Landspitali, The National University Hospital of Iceland, Reykjavík, Iceland
[45] Faculty of Medicine, University of Iceland, Reykjavík, Iceland.
[46] deCODE Genetics / Amgen, Reykjavík, Iceland.
[47] Department of Cardiology, Leiden University Medical Center, Leiden, the Netherlands.
[48] Department of Internal Medicine, Section of Gerontology and Geriatrics, Leiden University Medical Center, Leiden, The Netherlands.
[49] Centre for Bone and Arthritis Research, Department of Internal Medicine, Institute of Medicine, Sahlgrenska Academy, University of Gothenburg, Gothenburg, Sweden.
[50] Department of Internal Medicine, Academic Medical Center, Amsterdam, The Netherlands.
[51] Division of Medicne, Turku University Hospital, Turku, Finland.
[52] Department of Medicne, University of Turku, Turku, Finland.
[53] Department of Medicine, The University of Hong Kong, Hong Kong, China.
[54] Clinical and Molecular Osteoporosis Research Unit, Department of Clinical Sciences, Lund University, Malmö, Sweden.
[55] Department of Endocrinology, Odense University Hospital, Odense, Denmark.
[56] Molecular Genetics Section, Department of Pediatrics, University Medical Center Groningen, University of Groningen, Groningen, The Netherlands.
[57] Department of Genetics, Microbiology and Statistics, CIBERER, IBUB, IRSJD, University of Barcelona, Barcelona, Spain.
[58] Department of Applied Biomedical Science, Faculty of Health Sciences, University of Malta, Msida, Malta.
[59] Department of Medicine, University of Cantabria, Santander, Spain.
[60] Department of Internal Medicine, Hospital U M Valdecilla-IDIVAL, Santander, Spain.
[61] Musculoskeletal Research Group, IMIM (Hospital del Mar Medical Research Institute), Centro de Investigación Biomédica en Red en Fragilidad y Envejecimiento Saludable (CIBERFES), ISCIII, Barcelona, Catalonia, Spain.
[62] Department of Public Health and Clinical Medicine, Umeå University, Umeå, Sweden.
[63] Department of Radiation Sciences, Oncology, Umeå University, Umeå, Sweden.
[64] Institute of Biochemistry and Genetics, Ufa Scientific Center of RAS, Ufa, Russia.

[65] Division of Endocrinology and Metabolism, Asan Medical Center, University of Ulsan College of Medicine, Seoul, South Korea.
[66] Department of Medicine & Therapeutics, The Chinese University of Hong Kong, Hong Kong, China.
[67] Jockey Club Centre for Osteoporosis Care and Control, The Chinese University of Hong Kong, Hong Kong, China.
[68] Department of Surgical Sciences, Section of Orthopedics, University Hospital, Uppsala University, Uppsala, Sweden.
[69] Metabolic Bone Diseases Unit,AOUC, University Hospital of Florence, Forence, Italy.
[70] Azienda Ospedaliero Universitaria Careggi (AOUC), Florence, Italy.
[71] Clinical Biochemistry, University of Ljubljana, Ljubljana, Slovenia.
[72] Bioinformatics Core Facility, University of Gothenburg, Gothenburg, Sweden.
[73] Department of Genetics and Biotechnology, Faculty of Biology, University of Athens, Athens, Greece.
[74] Department of Endocrinology, Clinical Center Ljubljana, Ljubljana, Slovenia.
[75] Department of Epidemiology and Biostatistics, VU University Medical Center, Amsterdam, The Netherlands.
[76] Department of Surgical and Perioperative Sciences, Umeå University, Umeå, Sweden.
[77] INSERM UMR 1033, University of Lyon, Lyon, France.
[78] Department of Surgery and Translational Medicine, University of Florence, Florence, Italy.
[79] Faculty of Sport and Health Sciences, University of Jyväskylä, Jyväskylä, Finland.
[80] Nordic Bioscience A/S, Herlev, Denmark.
[81] MRC Lifecourse Epidemiology Unit, University of Southampton, Southampton, UK.
[82] Department of Nutrition and Dietetics, Harokopio University, Athens, Greece.
[83] Garvan Institute for Medical Research; School of Medicine Sydney, University of Notre Dame Australia, St Vincent's Hospital, Sydney, Australia.
[84] Unité de Recherche en Génétique Humaine et Moléculaire and Department of molecular biology, medical biochemistry and pathology, Faculty of Medicine, CHU de Québec–Université Laval, Québec City, Canada.
[85] Department of Medicine, McGill University, Montréal, Québec, Canada.
[86] Musculoskeletal Research Programme, Division of Applied Medicine, University of Aberdeen, Aberdeen, UK.
[87] Department of Medical Genetics, University of Antwerp, Antwerp, Belgium.
[88] Department of Endocrinology and Internal Medicine, Aarhus University Hospital, Aarhus, Denmark.
[89] School of Medical and Health Sciences, Edith Cowan University, Joondalup, WA, Australia.
[90] Medical School, University of Western Australia, Perth, Australia.
[91] School of Public Health, University of Sydney, Sydney, NSW, Australia.
[92] Department of Biochemistry & Experimental Medicine, The Children's Memorial Health Institute, Warsaw, Poland.
[93] Department of Genetics and Fundamental Medicine, Bashkir State University.
[94] Community Medicine and Rehabilitation, Umeå University, Umeå, Sweden.
[95] Department of Internal Medicine, Division of Endocrinology and Diabetology, Medical University Graz, Graz, Austria.
[96] Departments of Pharmacology and Clinical Neurosciences, Umeå University, Umeå Sweden.
[97] Department of Endocrinology and Diabetes, Sir Charles Gardiner Hospital, Perth, Australia.
[98] Nuffield Department of Orthopaedics, Rheumatology and Musculo-skeletal Science, University of Oxford, Oxford, UK.
[99] Department of Chemical Pathology, The Chinese University of Hong Kong, Hong Kong, China.
[100] Departments of Internal Medicine and Radiology, University of Manitoba, Winnipeg, Canada.
[101] San Francisco Coordinating Center, California Pacific Medical Center Research Institute, San Francisco, California, USA.
[102] Department of Epidemiology, Graduate School of Public Health, University of Pittsburgh, Pittsburgh, Pennsylvania, USA.
[103] Translational Genomics Group, Institute of Health and Biomedical Innovation, Faculty of Health, Queensland University of Technology, Translational Research Institute, Princess Alexandra Hospital,Brisbane, Australia.
[104] Department of Endocrinology and Diabetes, Royal Brisbane & Women's Hospital, Butterfield St, Herston, Australia.
[105] Department of Human Nutrition, Wageningen University, Wageningen, The Netherlands.
[106] Population and Medical Genetics, BROAD Institute, Cambridge, MA, USA.
[107] Laboratory of Epidemiology, National Institute on Aging, National Institutes of Health, Bethesda, Maryland, USA.
[108] Department of Medicine, Division of Endocrinology, Diabetes and Metabolism, The Ohio State University, Columbus, Ohio, USA.
[109] Faculty of Medicine in the Galilee, Bar-Ilan University, Safed , Israel.
[110] Clinical Gerontology Unit, University of Cambridge, Cambridge, UK.
[111] Fimlab Laboratories, Department of Clinical Chemistry, Tampere, Finland.
[112] Department of Clinical Chemistry, Finnish Cardiovascular Research Center - Tampere, Faculty of Medicine and Life Sciences, University of Tampere, Tampere, Finland.
[113] Department of Internal Medicine, VU University Hospital, Amsterdam, the Netherlands.
[114] Institute of Molecular and Cell Biology, University Of Tartu, Tartu, Estonia.
[115] Bone and Mineral Research Unit, Department of Medicine, Oregon Health & Science University, Portland, Oregon, USA.
[116] Cardiovascular Health Research Unit, Departments of Epidemiology, Health Services, University of Washington, Seattle, Washington, USA.
[117] Kaiser Permanente Washington Health Research Institute, Seattle, WA.
[118] Department of Clinical Physiology and Nuclear Medicine, Turku University Hospital, Turku, Finland.
[119] Research Centre of Applied and Preventive Cardiovascular Medicine, University of Turku, Turku, Finland.

[120] Department of Medicine, University of California, Davis, Sacramento, California, USA.
[121] Department of Twin Research and Genetic Epidemiology, King's College London, London, UK.
[122] Center for Evidence-based Medicine, Brown University School of Public Health, Providence, Rhode Island, USA.
[123] Stanford Prevention Research Center, Stanford University, Stanford, California, USA.

Research groups:

23andMe research team: Michelle Agee, Babak Alipanahi, Adam Auton, Robert K. Bell, Katarzyna Bryc, Sarah L. Elson, Pierre Fontanillas, Nicholas A. Furlotte, David A. Hinds, Karen E. Huber, Aaron Kleinman, Nadia K. Litterman, Matthew H. McIntyre, Joanna L. Mountain, Elizabeth S. Noblin, Carrie A.M. Northover, Steven J. Pitts, J. Fah Sathirapongsasuti, Olga V. Sazonova, Janie F. Shelton, Suyash Shringarpure, Chao Tian, Joyce Y. Tung, Vladimir Vacic, Catherine H. Wilson.

GEFOS/GENOMOS consortium: Katerina Trajanoska, John A. Morris, Ling Oei, Hou-Feng Zheng, Vincenzo Forgetta, Aaron Leong, Omar S. Ahmad, Charles Laurin, Lauren E. Mokry, Stephanie Ross, Cathy E. Elks, Jack Bowden, Nicole M. Warrington, Sara M. Willems , Daniel Wright, Felix R. Day, Anna Murray, Katherine S Ruth, Konstantinos K. Tsilidis, Cheryl L. Ackert-Bicknell, J.H. Duncan Bassett, Bram C.J. van der Eerden, Kaare M. Gautvik, Sjur Reppe, Graham R. Williams, Carolina Medina-Gómez, Karol Estrada, Najaf Amin, Joshua C. Bis, Stephan Breda, Daniel I. Chasman, Serkalem Demissie, Anke W. Enneman, Yi-Hsiang Hsu, Thorvaldur Ingvarsson, Mika Kähönen, Candace Kammerer, Andrea Z. LaCroix, Guo Li, Ching-Ti Liu, Yongmei Liu, Mattias Lorentzon, Reedik Mägi, Evelin Mihailov, Lili Mlani, Alireza Moayyeri, Carrie M. Nielson, Nerea Alonso, Pack Chung Sham, Kristin Siggeirsdotir, Gunnar Sigurdsson, Unnur Thorsteinsdottir, Stella Trompet, Gudmar Thorleifsson, Liesbeth Vandenput, Nathalie van der Velde, Jorma Viikari, Su-Mei Xiao, Jing Hua Zhao, Kristina E Akesson, Marianne Andersen, Biljana Atanasovska, Susana Balcells, Joel Eriksson, Melissa M. Formosa, Carmen Garcia-Ibarbia, Jesús Gonzalez-Macias, Natalia Garcia-Giralt, Goran Hallmans, Magnus Karlsson, Rita Khusainova, Beom-Jun Kim, Timothy C.Y. Kwok, Seung Hun Lee, Ping C. Leung, Hans Mallmin, Laura Masi, Beatrice S. Melin, Simona Mencej-Bedrac, Maria Nethander, José M. Olmos, Panagoula Kollia, Janez Prezelj, Natasja M. van Schoor, Olle Svensson, Pawel Szulc, Carmen Valero, Jean Woo, Maria Brandi, Sulin Cheng, Roland Chapurlat, Claus Christiansen, Cyrus Cooper, George Dedoussis, John A. Eisman, Morten Frost, Sylvie Giroux, Daniel Grinberg, David Goltzman, Lynne J. Hocking, Wim Van Hul, Jung-Min Koh, Lars Rejnmark, Jens-Erik B. Jensen, Bente Langdahl, Joshua R. Lewis ,Roman S. Lorenc, Elza Khusnutdinova, Janja Marc, Fiona E. McGuigan, Dan Mellström, Karl Michaelsson, Xavier Nogues, Peter Nordström, Barbara Obermayer-Pietsch, Ulrika Pettersson-Kymmer, Richard L. Prince, Jonathan Reeve, David M. Reid, Jose A. Riancho, Francois Rousseau, Nelson L.S. Tang, Angela Xuereb-Anastasi, William D. Leslie, Daniel S. Evans, Steven R. Cummings, Jane Cauley, Cornelia M van Duijn, Matt Brown, Emma L. Duncan, Lisette CPGM. de Groot, Tonu Esko, Vilmundar Gudnason, Tamara B. Harris, Rebecca D. Jackson, J. Wouter Jukema, M. Arfan Ikram, David Karasik, Stephen Kaptoge, Kay-Tee Khaw, Annie Wai-Chee Kung, Terho Lehtimäki, Leo-Pekka Lyytikäinen, Paul Lips, Robert Luben, Andres Metspalu, Joyce B. J. van Meurs, Ryan L Minster, Eric Orwoll, Edwin Oei, Bruce M. Psaty, Olli T. Raitakari, Stuart H. Ralston, Paul M. Ridker, John A. Robbins, Albert V. Smith, Tim D. Spector, Unnur Styrkarsdottir, Joseph Zmuda, Gregory J. Tranah, Kari Stefansson, Andre G. Uitterlinden, M. Carola Zillikens, Evangelia E. Ntzani, Evangelos Evangelou, John PA. Ioannidis, John R.B. Perry, Robert A Scott, David M. Evans, Douglas P. Kiel, Claes Ohlsson, J. Brent Richards, Fernando Rivadeneira.

II. Supplementary tables

Table S1A. Descriptive study design characteristics for Discovery Cohorts

Table S1B. Descriptive study design characteristics for Replication Cohorts

Table S2A. Discovery study-specific descriptive statistics

Table S2B. Replication study-specific descriptive statistics

Table S3A. Discovery study-specific fracture counts

Table S3B. Replication study-specific fracture counts

Table S4A. Genotyping/Imputation discovery

Table S4B. Genotyping GENOMOS replication

Table S4C. Genotyping/Imputation in silico replication

Table S5A. Number of SNPs and Variance explained by Instrumental Variables for Each Risk Factor

Table S5B. Individual summary statistics from the SNP-Exposure/Fracture association

Table S6A. Independent Signals from the discovery phase with p-values $< 5 \times 10^{-6}$

Table S6B. Signals with $p<5x10^{-8}$ in the discovery followed for replication in 23andMe

Table S6C. Meta-analysis results across stages for SNPs that did not replicate in GENOMOS

Table S6D. Previously identified BMD-associated loci with risk of any type of fracture

Table S7. Estimates of the Genetic Correlation between Other Clinical Risk Factors and BMD

Table S8. Estimating the effect of 15 Fracture Related Risk Factors on Fracture Risk

Table S9. Associations of different risk factors with bone mineral density

Table S1A | Descriptive Study Design Characteristics for Discovery Cohorts

Short name	Full name	Study Design	Country of Origin	City of Origin	Ethnicity	Study Type	Total Sample Size with DNA and Phenotype Information (N)	Study Description	References
**AGES**	Age, Gene/Environment Susceptibility Reykjavik Study	Cohort	Iceland	Reykjavik	Northern European	Population based	3185	The Age Gene/Environment Susceptibility Reykjavik Study originally comprised a random sample of 30,795 men and women born in 1907 1935 and living in Reykjavik in 1967. A total of 19,381 people attended, resulting in a 71% recruitment rate. The study sample was divided into six groups by birth year and birth date within month. One group was designated for longitudinal follow up and was examined in all stages; another was designated as a control group and was not included in examinations until 1991. Other groups were invited to participate in specific stages of the study. Between 2002 and 2006, the AGES Reykjavik study re-examined 5,764 survivors of the original cohort who had participated before in the Reykjavik Study.	[PMID: 17351290] (Harris, 2007 Age,Gene/Environment Susceptibility Reykjavik Study: multidisciplinary applied phenomics)
**AOGC**	Anglo-Australasian Osteoporosis Genetics Consortium	Population cohort, and case/control for fracture cases	Australia	Geelong	North-Western European	Population based, clinical based	2922	Population based BMD cohort (from electoral rolls) and case control for fracture cases; all drawn from Geelong general population of men and women	[PMID: 19707703] (Henry, 2010 Bone mineral density reference ranges for Australian men: Geelong Osteoporosis Study); [PMID: 11090233] (Henry, 2000 Prevalence of osteoporosis in Australian women: Geelong Osteoporosis Study)
**BPROOF**	B-vitamins for the PRevention Of Osteoporotic Fractures	Intervention study	The Netherlands	Wageningen/Amsterdam/Rotterdam (multi-center)	North-Western European	General population	2669	B-PROOF is a trial investigating the effect of 2-year supplementation with 400 mcg folic acid and 500 mcg vitamin B12 on fracture incidence in hyperhomoycsteinemic persons aged 65y and older.	[PMID: 22136481] (Rationale and design of the B-PROOF study, a randomized controlled trial on the effect of supplemental intake of vitamin B12 and folic acid on fracture incidence)

**CHS**	Cardiovascular Health Study	Cohort	Sacramento County, CA; Forsyth County, NC; Washington County, MD; Allegheny County, PA	Sacramento, Pittsburgh	European American	Population based	3261	A population based cohort study of risk factors for coronary heart disease and stroke in adults 65 years conducted across four field centers. The original predominantly Caucasian cohort was recruited in 1989 1990 from random samples of the Medicare eligibility lists. genotyping was performed at the General Clinical Research Center's Phenotyping/Genotyping Laboratory at Cedars Sinai using the Illumina 370CNV BeadChip system on 3980 CHS participants who were free of CVD at baseline, consented to genetic testing, and had DNA available for genotyping.	[PMID: 20031568] (Fried, 2009 Cohorts for Heart and Aging Research in Genomic Epidemiology (CHARGE) Consortium: Design of prospective meta analyses of genome wide association studies from 5 cohorts)
**DeCODE**	DeCODE Genetics Study	Cross sectional	Iceland	Reykjavik	North-Western European	Population based, clinical based		The study includes 40,000 individuals taking part in various disease projects	[PMID: 18445777] (Styrkarsdottir, 2008 Multiple genetic loci for bone mineral density and fractures); [PMID: 19079262] (Styrkarsdottir, 2009 Multiple genetic loci for bone mineral density and fractures)
**EGCUT-I**	Estonian Genome Center University of Tartu-I	Cohort	Estonia	Tartu	Northern European	Population based	4513	Estonian Biobank is a population-based biobank of the Estonian Genome Center at the University of Tartu (EGCUT). The total cohort size is currently 51,535 gene donors (≥18 years of age), which closely reflects the age, sex and geographical distribution of the Estonian population. Estonians represent 83%, Russians 14%, and other nationalities 3% of all participants. All subjects have been recruited randomly by general practitioners (GP) and physicians in hospitals.	[PMID: 19424496] (Nelis, 2009 Genetic structure of Europeans: a view from the North-East); [PMID: 24518929] (Leitsalu, 2014 Cohort Profile: Estonian Biobank of the Estonian Genome Center, University of Tartu)
**EGCUT-II**	Estonian Genome Center University of Tartu-II	Cohort	Estonia	Tartu	Northern European	Population based	1788	Estonian Biobank is a population-based biobank of the Estonian Genome Center at the University of Tartu (EGCUT). The total cohort size is currently 51,535 gene donors (≥18 years of age), which closely reflects the age, sex and geographical distribution of the Estonian population. Estonians represent 83%, Russians 14%, and other nationalities 3% of all participants. All subjects have been	[PMID: 19424496] (Nelis, 2009 Genetic structure of Europeans: a view from the North-East); [PMID: 24518929] (Leitsalu, 2014 Cohort Profile: Estonian Biobank of the Estonian Genome Center, University of Tartu)

								recruited randomly by general practitioners (GP) and physicians in hospitals.	
**EPICNOR**	European Prospective Investigation into Cancer, Norfolk study	Cohort	United Kingdom	Norfolk	North-Western European	Population based	20,663	A random sample of 3,036 men and women among the 20,663 participant EPIC Norfolk prospective study (all samples with DNA available and through QCs) were included with DXA measurements on BMD.	[PMID: 12753873] (Kaptoge, 2003 Effects of gender, anthropometric variables, and aging on the evolution of hip strength in men and women aged over 65); [PMID: 10466767] (Day, 1999 EPIC Norfolk: study design and characteristics of the cohort. European Prospective Investigation of Cancer); [PMID: 19079261] (Willer, 2009 Six new loci associated with body mass index highlight a neuronal influence on body weight regulation);
**ERF**	Erasmus Rucphen Family	Cohort	The Netherlands	Rucphen	North-Western European	Family based isolate	1602	A family based cohort study that is embedded in the Genetic Research in Isolated Populations (GRIP) program in the South West of the Netherlands. The aim of this program was to identify genetic risk factors in the development of complex disorders. For the ERF study, 22 families that had at least five children baptized in the community church between 1850 1900 were identified with the help of genealogical records. All living descendants of these couples and their spouses were invited to take part in the study. Data collection started in June 2002 and was finished in February 2005	[PMID: 15054401] (Aulchenko, 2004 Linkage disequilibrium in young genetically isolated Dutch population)
**FHS**	Framingham Heart Study	Cohort	United States of America	Framingham	European American	Population based, family based	3886	The Framingham Osteoporosis Study is an ancillary study of the parent, Framingham Study. The Framingham Study is a family based, multigenerational cohort study initiated originally to study the risk factors for cardiovascular disease	[PMID: 14819398] (Dawber, 1951 Epidemiological approaches to heart disease: the Framingham Study); [PMID: 474565] (Kannel, 1979 An investigation of coronary heart disease in families. The Framingham offspring study) [PMID: 17372189] (Splansky, 2007 The Third Generation

									Cohort of the National Heart, Lung, and Blood Institute's Framingham Heart Study: design, recruitment, and initial examination)
**GOOD**	Gothenburg Osteoporosis and Obesity Determinants Study	Cohort	Sweden	Gothenburg	Northern European	Population based	938	A study initiated to determine both environmental and genetic factors involved in the regulation of bone and fat mass.	[PMID: 16007330] (2005, Free testosterone is a positive whereas free estradiol is a negative predictor of cortical bone size in young Swedish men The GOOD Study)
**HABC**	Health Aging and Body Composition	Cohort	United States of America	Pittsburgh, PA; Memphis, TN	European American	Population based	1649	A population based, prospective cohort study of well-functioning, unrelated men and women aged 70 and older. It was initiated to assess changes in body composition.	[PMID: 12028178] (Visser, 2002 Leg muscle mass and composition in relation to lower extremity performance in men and women aged 70 to 79: the health, aging and body composition study); [PMID: 16043679] (Strotmeyer, 2005 Nontraumatic fracture risk with diabetes mellitus and impaired fasting glucose in older white and black adults: the health, aging, and body composition study); [PMID: 15176990] (Strotmeyer, 2004 Diabetes is associated independently of body composition with BMD and bone volume in older white and black men and women: The Health, Aging, and Body Composition Study)
**HKOS**	Case control	Case/ control	China	Hong Kong	Southern Han Chinese	Population based, clinic based	800	A sample of 800 unrelated subjects with extreme BMD phenotype (Z score 1.28 or +1.0 at either lumbar spine or femoral neck) were selected from a growing database of Hong Kong Southern Chinese (more than 7,000 volunteers)	[PMID:20096396] (Kung, 2010 Association of JAG1 with bone mineral density and osteoporotic fractures: a genome wide association study and follow up replication studies);

**MROS**	MrOS	Cohort	United States of America	Birmingham, AL; Minneapolis, MN; Palo Alto, CA; Monongahela Valley near Pittsburgh, PA; Portland, OR; and San Diego, CA	European American	Clinic based	4473	The Osteoporotic Fractures in Men Study (MrOS) is a multi-center prospective, longitudinal, observational study of risk factors for vertebral and all non-vertebral fractures in older men, and of the sequelae of fractures in men. The MrOS study population consists of community dwelling, ambulatory men aged 65 years or older from six communities in the United States. Inclusion criteria were designed to provide a study cohort that is representative of the broad population of older men. The inclusion criteria were: (1) ability to walk without the assistance of another, (2) absence of bilateral hip replacements, (3) ability to provide self-reported data, (4) residence near a clinical site for the duration of the study, (5) absence of a medical condition that (in the judgment of the investigator) would result in imminent death, (6) ability to understand and sign an informed consent, and (7) 65 years or older. To qualify as an enrollee, the participant had to provide written informed consent, complete the self-administered questionnaire (SAQ), attend the clinic visit, and complete at least the anthropometric, DEXA, and vertebral X-ray procedures. The MrOS cohort recruited only men.	[PMID: 16598372] (Mellström, 2006 Free testosterone is an independent predictor of BMD and prevalent fractures in elderly men: MrOS Sweden)
**PROSPER**	The PROpective Study of Pravastatin in the Elderly at Risk	Cohort, randomized controlled trial	The Netherlands/ United Kingdom /Ireland	Leiden/ Glasgow/ Cork	North-Western European	Clinic based	5242	A randomized controlled clinical trial to test the effect of pravastatin on cardiovascular outcomes in the elderly at risk.	[PMID: 12457784] (Shepherd, 2002 Pravastatin in elderly individuals at risk of vascular disease (PROSPER): a randomised controlled trial)
**RS I**	Rotterdam Study I	Cohort	The Netherlands	Rotterdam	North-Western European	Population based	5746	A prospective population based cohort study of chronic disabling conditions in Dutch elderly individuals aged 55 years and over. The RS III cohort included individuals aged 45 years and over.	[PMID:19700477] (Estrada, 2009 GRIMP: a web and grid based tool for high speed analysis of large scale genome wide association using imputed data); [PMID:19728115] (Hofman, 2009 The Rotterdam Study: 2010 objectives and design update); [PMID:1833235] (Hofman, 1991

									Determinants of disease and disability in the elderly: the Rotterdam Elderly Study);
**RS II**	Rotterdam Study II	Cohort	The Netherlands	Rotterdam	North-Western European	Population based	2157	A prospective population based cohort study of chronic disabling conditions in Dutch elderly individuals aged 55 years and over. The RS III cohort included individuals aged 45 years and over.	[PMID:19700477] (Estrada, 2009 GRIMP: a web and grid based tool for high speed analysis of large scale genome wide association using imputed data); [PMID:19728115] (Hofman, 2009 The Rotterdam Study: 2010 objectives and design update); [PMID:1833235] (Hofman, 1991 Determinants of disease and disability in the elderly: the Rotterdam Elderly Study);
**RS III**	Rotterdam Study III	Cohort	The Netherlands	Rotterdam	North-Western European	Population based	1212	A prospective population based cohort study of chronic disabling conditions in Dutch elderly individuals aged 55 years and over. The RS III cohort included individuals aged 45 years and over.	[PMID:19700477] (Estrada, 2009 GRIMP: a web and grid based tool for high speed analysis of large scale genome wide association using imputed data); [PMID:19728115] (Hofman, 2009 The Rotterdam Study: 2010 objectives and design update); [PMID:1833235] (Hofman, 1991 Determinants of disease and disability in the elderly: the Rotterdam Elderly Study);

**SOF**	Study of Osteoporotic Fractures	Cohort	United States of America	Baltimore, Maryland; Minneapolis, Minnesota; Portland, Oregon; and the Monongahela Valley, Pennsylvania	European American	Clinic based	3309	The Study of Osteoporotic Fractures (SOF) is a prospective multicentre study of risk factors for vertebral and non-vertebral fractures. The cohort is comprised of community dwelling women 65 years old or older recruited from populations-based listings in four U.S. areas. The SOF participants were followed up every four months by postcard or telephone to ascertain the occurrence of falls, fractures and changes in address. To date, follow-up rates have exceeded 95% for vital status and fractures. All fractures are validated by x-ray reports or, in the case of most hip fractures, a review of pre-operative radiographs. The inclusion criteria were: 1) 65 years or older, (2) ability to walk without the assistance of another, (3) absence of bilateral hip replacements, (4) ability to provide self-reported data, (5) residence near a clinical site for the duration of the study, (6) absence of a medical condition that (in the judgment of the investigator) would result in imminent death, and (7) ability to understand and sign an informed consent.	[PMID: 7862179] (Cummings, 1995 Risk factors for hip fracture in white women. Study of Osteoporotic Fractures Research Group)
**TUK123**	TwinsUK	Cohort	United Kingdom	London	North-Western European	Population based, family based	4312	TwinsUK is a population based registry of British Twins representative of the general British population.	[PMID: 19841454] (Richards, 2009 Collaborative meta-analysis: associations of 150 candidate genes with osteoporosis and osteoporotic fracture); [PMID: 18455228] (Richards, 2008 Bone mineral density, osteoporosis, and osteoporotic fractures: a genome wide association study)
**UKBB**	UK Biobank	Cohort	United Kingdom	National	Mixed (North-Western European subset used for analysis)	Cohort	498,933	In 2006-2010, the UK Biobank recruited 502,643 individuals aged between 37-73 years (99.5% were 40-69 years) from across the country. Each participant provided a large amount of information regarding their health and lifestyle using touch screen questionnaires, physical measurements and agreement to have their health	Biobank UK. UK Biobank: Protocol for a Large-scale Prospective Epidemiological Resource. 2010. http://www.ukbiobank.ac.uk/wp-content/uploads/2011/11/UKBiobank-Protocol.pdf

								followed and they also provided blood, urine and saliva samples for future analysis. A subset of 152,248 individuals currently have genetic data available from the May 2015 release.	
WGHS	Women's Genome Health Study	Cohort	United States of America	National	European American	Population based	22330	A population based cohort derived from the approximately 72% of women who provided a blood sample in the Women's Health Study, a trial of aspirin and vitamin E in prevention of cardiovascular disease and cancer among middle aged, female health care professionals. The WGHS now has over 20 years of follow up for incident clinical events, including bone fracture	[PMID: 18070814] (Ridker, 2008 Women's Genome Health Study Working Group. Rationale, design, and methodology of the Women's Genome Health Study: a genome wide association study of more than 25,000 initially healthy american women)
**WHICT**	Women's Health Initiative Clinical Trial	Quasi case /control	United States of America	Multi center (n=3)	European American	Population based	3923	Hip fracture portion of GeCHIP. This is a case control sample from the Women's Health Initiative. All hip fractures in the WHI Clinical Trial (CT) and Observational Study (OS) through Aug2007 were selected and matched to controls. In the OS, controls were matched on age (+/ 1yr), race/ethnicity (exact), enrolment date (+/ 365 days) and current HT use at baseline (exact). For the CT age (+/ 1yr), race/ethnicity (exact), earliest randomization date (+/ 365 days), HT use (active vs. placebo, or if not enrolled current HT use at baseline; exact) and CaD use (active vs. placebo vs. not enrolled; exact). Controls were excluded if they self-reported a prior history of postmenopausal fracture (age >= 55 years)	[PMID: 9492970] (1998, Design of the Women's Health Initiative clinical trial and observational study. The Women's Health Initiative Study Group)
**WHIOS**	Women's Health Initiative Observational Study	Quasi case /control	United States of America	Multi center (n=3)	European American	Population based	3923	Hip fracture portion of GeCHIP. This is a case control sample from the Women's Health Initiative. All hip fractures in the WHI Clinical Trial (CT) and Observational Study (OS) through Aug2007 were selected and matched to controls. In the OS, controls were matched on age (+/ 1yr), race/ethnicity (exact), enrolment date (+/ 365 days) and current HT use at baseline (exact). For the CT age (+/ 1yr), race/ethnicity (exact), earliest randomization date (+/ 365 days), HT	[PMID: 9492970] (1998, Design of the Women's Health Initiative clinical trial and observational study. The Women's Health Initiative Study Group)

								use (active vs. placebo, or if not enrolled current HT use at baseline; exact) and CaD use (active vs. placebo vs. not enrolled; exact). Controls were excluded if they self-reported a prior history of postmenopausal fracture (age >= 55 years)	
**YFS**	CV risk in Young Finns Study	Cohort	Finland	Multicenter	Northern European	Population based	1586	The Cardiovascular Risk in Young Finns Study is one of the largest follow-up studies into cardiovascular risk from childhood to adulthood. The main aim of the Young Finns Study is to determine the contribution made by childhood lifestyle, biological and psychological measures to the risk of cardiovascular diseases in adulthood.	[PMID: 18263651] (Raitakari, 2008 Cohort profile: the cardiovascular risk in Young Finns Study)

Table S1B | Descriptive Study Design Characteristics for Replication Cohorts

Short name	*Type*	Study Name	Study Design	Country of Origin	City of Origin	Ethnicity	Study Type	Total Sample Size with DNA and Phenotype Information (N)	Short Study Description	References
**23andMe**	*in silico*	23andMe, Inc.	Cohort	United States of America	Mountain View	European	Population based	297,285	All participants were drawn from the customer base of 23andMe, Inc., a consumer genetics company. This cohort has been described in detail previously (Refs). Participants provided informed consent and participated in the research online, under a protocol approved by the external AAHRPP-accredited IRB, Ethical & Independent Review Services (E&I Review).	Tung JY, Do CB, Hinds DA, Kiefer AK, Macpherson JM, Chowdry AB, Francke U, Naughton BT, Mountain JL, Wojcicki A, Eriksson N. "Efficient replication of over 180 genetic associations with self-reported medical data." PLoS One. 2011;6(8):e23473.; Eriksson N, Macpherson JM, Tung JY, Hon LS, Naughton B, Saxonov S, Avey L, Wojcicki A, Pe'er I, Mountain J. "Web-based, participant-driven studies yield novel genetic associations for common traits."PLoS Genet. 2010 Jun 24;6(6):e1000993.
**AOGC-GOS**	*de-novo*	Anglo-Australasian Osteoporosis Genetics Consortium - Geelong Osteoporosis Study	Population cohort, and case/control for fracture cases	Australia	Geelong	North-Western European	Population based, clinical-based	2922	Population-based BMD cohort (from electoral rolls) and case-control for fracture cases; all drawn from Geelong general population of men and women	[PMID: 19707703] (Henry, 2010 Bone mineral density reference ranges for Australian men: Geelong Osteoporosis Study); [PMID: 11090233] (Henry, 2000 Prevalence of osteoporosis in Australian women: Geelong Osteoporosis Study)
**AOGC-SHEFFIELD**	*de-novo*	Anglo-Australasian Osteoporosis Genetics Consortium - Sheffield	Cohort	United Kingdom	Sheffield	North-Western European	Population based	4014	Large population-based cohort of community-dwelling elderly women aged ≥ 75 years	[PMID: 17042717] (McCloskey, 2007 Clodronate reduces the incidence of fractures in community-dwelling elderly women unselected for osteoporosis: results of a double-blind, placebo-controlled randomized study)

**APOSS**	*de-novo*	Aberdeen Prospective Osteoporosis Screening Study	Cohort	United Kingdom	Aberdeen	North-Western European	Population based	3268	APOSS is a longitudinal population-based study of osteoporotic fracture risk assessment in Caucasian women aged 45-54 years of age at baseline.	[PMID: 16355284] (Macdonald, 2006 Large-scale population-based study shows no evidence of association between common polymorphism of the VDR gene and BMD in British women)
**AROS**	*de-novo*	Aarhus Osteoporosis Study	Case control	Denmark	Aarhus	Northern European	Clinic based	801	Men and women with and without osteoporosis	[PMID: 16299058] (Gugatschka, 2002 Molecularly defined lactose malabsorption, milk consumption and anthropometric differences in adult males); [PMID: 14753735] (Obermayer Pietsch, 2004 Genetic predisposition for adult lactose intolerance and relation to diet, bone density, and bone fractures)
**AUSTRIOS-A**	*de-novo*	Austrios A "Young cohort"	Cohort	Austria	Graz	Central European	General population	893	Men and women with and without osteoporosis	[PMID: 18349089] (van Meurs, 2008 Large-scale analysis of association between LRP5 and LRP6 variants and osteoporosis)
**AUSTRIOS-B**	*de-novo*	Austrios B "old cohort"	Cohort	Austria	Graz	Central European	Nursing home patients	1158	95 nursing homes in Austria, patients had to be relatively healthy	[PMID: 18349089] (van Meurs, 2008 Large-scale analysis of association between LRP5 and LRP6 variants and osteoporosis)
**BARCOS**	*de-novo*	Barcelona Cohorte Osteoporosis	Cohort	Spain	Barcelona	Mediterranean European	General population	1400	The Barcelona is a cohort study of unrelated women aged 44 years and over that were recruited from the Menopausal Unit of the Hospital del Mar, Barcelona. All of the participants were consecutive, unselected, postmenopausal women who had presented to the outpatient clinic for a baseline visit due to menopause.	[PMID: 17878995] (Bustamante, 2007 Promoter 2 -1025 T/C polymorphism in the RUNX2 gene is associated with femoral neck bmd in Spanish postmenopausal women); [PMID: 17984249] (Bustamante, 2007 Polymorphisms in the interleukin-6 receptor gene are associated with bone mineral density and body mass index in Spanish postmenopausal women)

**CABRIO-C**	*de-novo*	Cantabria-Camargo	Cross-sectional	Spain	Santander	Mediterranean European	Community based	1450	Community-based study designed to evaluate the prevalence of metabolic bone diseases in postmenopausal women and men older than 50 attended at a primary care center in Northern Spain.	[PMID: 20594548] (Olmos, 2010 Bone turnover markers in Spanish adult men The Camargo Cohort Study); [PMID: 19737549] (Martínez, 2009 Bone turnover markers in Spanish postmenopausal women: the Camargo cohort study)
**CABRIO-CC**	*de-novo*	Cantabria Osteoporosis Case-control	Case-control, cross-sectional	Spain	Santander	Mediterranean European	Clinic based plus volunteers	2321	Clinic-based study of control individuals and patients with osteoporosis living in Cantabria, a region in Northern Spain	[PMID: 17118999] (Riancho, 2007 Identification of an aromatase haplotype that is associated with gene expression and postmenopausal osteoporosis); [PMID: 17218734] (Zarrabeitia, 2007 Adiposity, estradiol, and genetic variants of steroid-metabolizing enzymes as determinants of bone mineral density
**LSAW (CAIFOS)**	*de-novo*	Longitudinal Study of Aging in Women Calcium Intake Fracture Outcome Study	Cohort, randomized controled trial	Australia	Perth	North-Western European	Population based	1347	Randomized controled trial and cohort study	[PMID: 16636212] (Prince, 2006 Effects of calcium supplementation on clinical fracture and bone structure: results of a 5 year, double blind, placebo controlled trial in elderly women)
**CALEX**	*de-novo*	Calex-family study	Cohort	Finland	Jyväskylä and its surrondings	Northern European	General population	994	The Calex-family study is a family-based study to study Fractures in Puberty - Causes and Implications in Old Age	[PMID: 19171028] (Cheng, 2009 Trait-specific tracking and determinants of body composition: a 7-year follow-up study of pubertal growth in girls); [PMID: 19481189] (Cheng, 2009 Low volumetric BMD is linked to upper-limb fracture in pubertal girls andpersists into adulthood: a seven-year cohort study); [PMID: 20200961] (Wang, 2010 Familial resemblance and diversity in bone mass and strength in the population are established during the first year of postnatal life)

**CAMOS**	*de-novo*	Canadian Multicentre Osteoporosis Study	Cohort	Canada	Vancouver, Calgary, Saskatoon, Hamilton, Toronto, Kingston, Québec City, Halifax, St John's	European Canadian	General population	2388	The CaMos Study is a population-based, randomly selected, prospective cohort study from 9 Canadian cities followed for 14 years for osteoporosis-related traits and outcomes.	[PMID: 11199195] (Tenenhouse, 2000 Estimation of the prevalence of low bone density in Canadian women and men using a population-specific DXA reference standard: the Canadian Multicentre Osteoporosis Study (CaMos)); [PMID: 17129177] (Richards, 2007 Changes to osteoporosis prevalence according to method of risk assessment);(Berger C 2008 Change in bone mineral density as a function of age in women and men and association with the use of antiresorptive agents [PMID: 17242321] (Richards, 2007 Effect of selective serotonin reuptake inhibitors on the risk of fracture) [PMID: 18559803]
**DOES**	*de-novo*	Dubbo Osteoporosis Epidemiology Study	Cohort	Australia	Sydney (Dubbo)	North-Western European	Population based, family based	1457	A cohort study of approximately 2/3rds of the men and women in Dubbo, aged 60 years or older from 1989 every 2 years to the present. Data collected include BMD, life style, medical assessment, medication use and a wide range of health conditions and outcomes. It has been extended recently to include any person older than 20 years	[PMID: 19190316] (Bliuc, 2009 Mortality risk associated with low-trauma osteoporotic fracture and subsequent fracture in men and women); [PMID: 19419321] (Frost, 2009 Timing of repeat BMD measurements: development of an absolute risk-based prognostic model)
**DOPS**	*de-novo*	Danish Osteoporosis Prevention Study Cohort	Cohort	Denmark	Aarhus, Odense, Cope	Northern European	Population based	1716	Population based study of perimenopausal women. The women were followed for 10 years. App. 35% were treated with HRT	[PMID: 10340280] (Mosekilde, 1999 The Danish Osteoporosis Prevention Study (DOPS): project design and inclusion of 2000 normal perimenopausal women)
**EDOS**	*de-novo*	Edinburgh Osteoporosis Study	Cross-sectional	UK	Edinburgh and Lothian	North-Western European	Clinical referral population (patients)	2024	Clinical referral population of patients assessed for evaluation of osteoporosis	None

**EPICNOR**	*de-novo*	European Prospective Investigation of Cancer (Norfolk, UK cohort)	Cohort	UK	Norfolk	North-Western European	General population	3977	EPIC-Norfolk was conceived as part of a large Europe-wide study into the dietary and lifestyle determinants of cancer and had recruited 25,311 men and women aged 45-74 years. It was later broadened to include collaborative studies into other disease end-points including cardiovascular disease, diabetes, and bone fragility. The bone fragility study was initiated some 18 months after the inception of EPIC-Norfolk and recruited a random sample of 1511 men and women in the top decade of age (i.e. aged 65-74 at recruitment to EPIC-Norfolk).	[PMID: 12753873] (Kaptoge, 2003 Effects of gender, anthropometric variables, and aging on the evolution of hip strength in men and women aged over 65); [PMID: 10466767] (Day, 1999 EPIC-Norfolk: study design and characteristics of the cohort. European Prospective Investigation of Cancer); [PMID: 19079261] (Willer, 2009 Six new loci associated with body mass index highlight a neuronal influence on body weight regulation)
**EPOLOS**	*de-novo*	Early risk identification and effective prevention of osteoporosis based bone fractures in Polish population.	Cross-sectional	Poland	Warsaw, Lodz, Poznan, Krakow, Wroclaw, Bydgoszcz	Central European	Population based	715	The EPOLOS Study is a population-based, cross-sectional study of unrelated men and women aged 19-81 years, initiated to identify early risk and effective prevention of osteoporosis based bone fractures in Polish population.	[PMID: 20502405] (Skowrońska-Jóźwiak, 2010 Comparison of selected methods for fracture risk assessment in postmenopausal women: analysis of the Łódź population in the EPOLOS study); [PMID: 20502404] (Skowrońska-Jóźwiak, 2010 Effect of sex, age, and anthropometric parameters on the size and shape of vertebrae in densitometric morphometry: results of the EPOLOS study); [PMID: 19396748] (Skowrońska-Jóźwiak, 2009 Identification of vertebral deformities in the Polish population by morphometric X-ray absorptiometry - results of the EPOLOS study)

**EPOS**	*de-novo*	European Prospective Osteoporosis Study	Cohort	18 centres across 13 countries in Europe	Europe	European	General population	2106	EPOS was an extension of the European Vertebral Osteoporosis Study (EVOS) study and aimed to quantify incidence of vertebral and non-vertebral fractures. EVOS had recruited some 17,342 men and women aged over 50 years from 36 centres in 19 European countries. Each centre had recruited a random sample of up to 300 men and 300 women from population registers stratified into six 5-year age bands: 50-54, ..., 70-74 and 75+. A total of 7,273 participants from 31 EVOS centres took part in the EPOS follow up study.	[PMID: 8797123] (O'Neill, 1996 The prevalence of vertebral deformity in european men and women: the European Vertebral Osteoporosis Study); [PMID: 10824241] (Ismail, 2000 Validity of self-report of fractures: results from a prospective study in men and women across Europe. EPOS Study Group. European Prospective Osteoporosis Study Group); [PMID: 11918229] (EPOS study group, 2002 Incidence of vertebral fracture in europe: results from the European Prospective Osteoporosis Study (EPOS))
**FLOS**	*de-novo*	FLORENCE study	Cohort	Italy	Florence	Southern European	Population based	1000	The FLOS Study is a population based cohort study of unrelated men and women aged 50 years and over, collected to perform genetic studies in osteoporosis.	[PMID: 11344237] (Masi, 2001 Polymorphism of the aromatase gene in postmenopausal Italian women: distribution and correlation with bone mass and fracture risk)
**GEOS**	*de-novo*	Quebec sample	Cohort	Canada	Quebec	Western European	General population	2379	Population-based sample collected for the sudy of bone mineral variation. Only women from 18 to 84 years	[PMID: 19821770] (Elfassihi, 2010 Association with replication between estrogen-related receptor gamma (ESRRgamma) polymorphisms and bone phenotypes in women of European ancestry)
**GEVUR**	*de-novo*	Institute of Biochemistry and Genetics Ufa Scientific Centre RAS	case-control	Russia	Ufa	Russians, Tatars	General population, patients	999	The GEVUR Study is case-control and population-based, prospective cohort study of unrelated women aged 50 years and over, men with osteoporotic fractures and healthy men	[PMID: 15657606] (Laan, 2005 X-chromosome as a marker for population history: linkage disequilibrium and haplotype study in Eurasian populations); [PMID: 16465065] (Kutuev, 2006 From East to West: patterns of genetic diversity of populations living in four Eurasian regions); [PMID:18619040] (Selezneva, 2008 Association of polymorphisms and

										haplotypes in the 5' region of COLIA1 gene with the risk of osteoporotic fractures in Russian women from Volga-Ural region)
**GEVUR-2**	*de-novo*	Institute of Biochemistry and Genetics Ufa Scientific Centre RAS	case-control	Russia	Ufa	Russians, Tatars	General population, patients	999	The GEVUR Study is case-control and population-based, prospective cohort study of unrelated women aged 50 years and over, men with osteoporotic fractures and healthy men	[PMID: 15657606] (Laan, 2005 X-chromosome as a marker for population history: linkage disequilibrium and haplotype study in Eurasian populations); [PMID: 16465065] (Kutuev, 2006 From East to West: patterns of genetic diversity of populations living in four Eurasian regions); [PMID:18619040] (Selezneva, 2008 Association of polymorphisms and haplotypes in the 5' region of COLIA1 gene with the risk of osteoporotic fractures in Russian women from Volga-Ural region)
**GROS**	*de-novo*	Genetic Analysis of Osteoporosis in Greece	Cohort, case-control	Greece	Athens	Mediterranean European	General population, patients	491	The GROS study is a population-based, prospective cohort study of unrelated Greek men and women aged 43 years and over who visited the Department of Orthopaedic Surgery, University Hospital of Thessalia, Larissa, Greece.	None
**HCS**	*de-novo*	Hertfordshire Cohort Study	Cohort	UK		North-Western European	General population	2973	The Hertfordshire Cohort Study is a population-based cohort study of men and women born between 1931 and 1939 in the county of Hertfordshire, UK. It was initiated to evaluate interactions between the genome, the intrauterine and early postnatal development, and adult diet and lifestyle in the aetiology of chronic disorders in later life.	[PMID: 15964908] (Syddall, 2005 Cohort Profile: The Hertfordshire Cohort Study)
**HKOS**	*de-novo*	Chinese community elderly Men and Women study	Cohort	Hong Kong, China	Hong Kong, China	Chinese	General population	4000	Two thousand Chinese men and two thousand Chinese women living in the community, aged 65 years and above, were recruited by posting public advertisements at community centers for the elderly and housing estates in Hong Kong since 2001.	[PMID: 20949110;] (Styrkarsdottir, 2010 European bone mineral density loci are also associated with BMD in East-Asian populations); [PMID: 19766747] (Tang, 2010 Sex-specific effect of

										Pirin gene on bone mineral density in a cohort of 4000 Chinese)
**KORAMC**	*de-novo*	Korean osteoporosis study at Asan Medical Center	Cross sectional	Korea	Seoul	East Asian, Korean	Clinic based	1397	KorAMC study is a hospital registered, cross sectional study of postmenopausal Korean women	[PMID: 17620055] (Koh, 2007 Association of FLT3 polymorphisms with low BMD and risk of osteoporotic fracture in postmenopausal women)
**LASA**	*de-novo*	Longitudinal Aging Study Amsterdam	Cohort	The Netherlands	Amsterdam, Zwolle, Oss and surroundings	North-Western European	General population	831	LASA is an ongoing multidisciplinary cohort study on predictors and consequences of changes in physical, cognitive, emotional and social functioning in older persons.	[PMID: 11927198] (Deeg, 2002 Attrition in the Longitudinal Aging Study Amsterdam: The effect of differential inclusion in side studies)
**MANMC**	*de-novo*	Manitoba McGill Fracture Study	Cross-Sectional	Canada	Winnipeg	North-Western European	General Population	1062	The Manitoba-McGill Fracture Study is a population-based sample of women experiencing a validated clinical hip or forearm fracture requiring surgical intervention.	[PMID: 21124974] (Ladouceur, 2010 An Efficient Paradigm for Genetic Epidemiology Cohort Creation)
**MINOS**	*de-novo*	MINOS	Cohort	France	Montceau les Mines	Western European	General Population	495	MINOS is a prospective cohort study of male osteoporosis. Its aim was to assess predictors of bone loss and fractures in men. Participants were recruited in 1995 to 1996 from the Société de Secours Minière de Bourgogne (SSMB) rolls in Montceau les Mines. Letters inviting participation were sent to a randomly selected sample of 3400 clients of SSMB aged 50 to 85 years. Among them, 841 agreed to participate and provided informed consent. Forty-three men refused bone densitometry or had radiographs of poor quality. The cohort was followed up for 7.5 years (questionnaire and DXA every 18 months, spine radiograph after 3 and 7.5 years). Then, for 2.5 years, the men were followed up to obtain information on incident nonvertebral fractures. Men were followed to the first of the following: fracture, last contact, death, or end of follow-up. Men were followed to the first of the following: fracture, last contact, death, or end of follow-up.	[PMID: 10678406] (Szulc, 2000 Cross-sectional assessment of age-related bone loss in men)

**MOFS**	*de-novo*	Malta Osteoporotic Fracture Study	Case-Control	Malta	Msida	Southern European	General population	1046	For the Malta Osteoporotic Fracture Study eligible postmenopausal women referred by medical practitioners to the Bone Density Unit at the Department of Obstetrics and Gynaecology, Mater Dei Hospital, Malta, were recruited as controls. Fracture cases were recruited from the Bone Density Unit, Orthopaedic department (Mater Dei Hospital), and Karin Grech Rehabilitation Hospital. All research subjects studied were healthy Caucasian women with ages ranging from 40–79 years.	[PMID: 26400554] (Formosa, 2016 Biochemical predictors of low bone mineral density and fracture susceptibility in Maltese postmenopausal women); [PMID: 21994215] (Vidal, 2011 Functional polymorphisms within the TNFRSF11B (osteoprotegerin) gene increase the risk for low bone mineral density) [PMID: 19580891] (Vidal, 2009 Effects of a synonymous variant in exon 9 of the CD44 gene on pre-mRNA splicing in a family with osteoporosis)
**MROSS**	*de-novo*	MrOS Sweden	Cohort	Sweden	Gothenburg, Uppsala and Malmö	Northern European	Population-based	2924	The Osteoporotic Fractures in Men (MrOS) study is a multicenter, prospective study including 3,014 elderly men in Sweden, Hong Kong (~2,000), and the United States (~6,000). The MrOS Sweden cohort consist of three sub-cohorts from three different Swedish cities (n=1,005 in Malmö, n=1,010 in Göteborg, and n=999 in Uppsala). Study subjects (men aged 69–80 years) were randomly identified using national population registers, contacted and asked to participate. To be eligible for the study, the subjects had to be able to walk without assistance, provide self-reported data, and sign an informed consent; there were no other exclusion criteria. The study was approved by the ethics committees at the Universities of Gothenburg, Lund, and Uppsala. Informed consent was obtained from all study participants.	[PMID: 16598372] (Mellström, 2006 Free testosterone is an independent predictor of BMD and prevalent fractures in elderly men: MrOS Sweden)

**NOSOS**	*de-novo*	North of Scotland Osteoporosis Study	Cohort	United Kingdom	Aberdeen, Dingwall	North-Western European	Population-based	1293	NOSOS is a population-based osteoporosis screening programme of postmenopausal females aged 60-82 years of age at baseline.	[PMID: 18633668] (Mavroeidi, 2009 Physical activity and dietary calcium interactions in bone mass in Scottish postmenopausal women); [PMID: 20966103] (Judson, 2010 The Functional ACTN3 577X Variant Increases the Risk of Falling in Older Females: Results From Two Large Independent Cohort Studies)
**OAS**	*de-novo*	Odense Androgen Study	Cross-sectional	Denmark	NA	Northern European	Population-based	581	The Odense Androgen Study (OAS) is a population-based, prospective, observational study on endocrine status and bone metabolism in older men. 4875 males aged 60–74 years were randomly selected from the civil registration database in Funen County, Denmark, and invited by mail to participate in the study. A total of 3743 men returned the questionnaire, and 600 were included in the study.	[PMID: 16418764] (Nielsen, 2006 Prevalence of overweight, obesity and physical inactivity in 20- to 29-year-old, Danish men. Relation to sociodemography, physical dysfunction and low socioeconomic status: the Odense Androgen Study); [PMID: 23370486] (Harsløf, 2013 Polymorphisms of muscle genes are associated with bone mass and incident osteoporotic fractures in Caucasians)
**OPRA**	*de-novo*	Osteoporosis Population-based Risk Assessment Trial	Cohort	United States of America	Western Washington State	European American	Clinical based	972	The OPRA Trial was conducted at Group Health Cooperative (GHC), a mixed-model health maintenance organization in western Washington State with more than 47,000 women enrollees aged 60 and older. Women aged 60–80 were eligible to participate if they had not taken hormone replacement therapy or other osteoporosis medications for at least 12 months. Potentially eligible women were identified from GHC enrollment files on the basis of age and computerized pharmacy information. All women who participated in the bone density testing examination completed a detailed baseline questionnaire assessing their demographic characteristics, fracture risk factors including previous fractures. Hip and other fracture events	[PMID: 15725986] (Lacroix, 2005 Evaluation of three population-based strategies for fracture prevention: results of the osteoporosis population-based risk assessment (OPRA) trial)

									were regarded as exploratory outcomes and ascertained through December 31, 2000, by searching automated hospitalization and outpatient visit records for ICD-9 codes indicating the occurrence of nonpathologic fracture.	
**OSTEOS-II**	*de-novo*	Osteoporosis: SNPs To Environment Study	Cross-sectional	Greece	Athens	Mediterranean European	Population-based	564	OSTEOS is a cross-sectional study of unrelated men and women, aimed to assess genetic and enrironmental factors, especially nutrition, and their possible interactions on QUS parameters (BUA, SOS, SI)	
**PERF**	*de-novo*	Prospective Epidemiological Risk Factor	Randomized-controled trial	Denmark	Copenhagen	Northern European	Clinical-based	3973	The Prospective Epidemiological Risk Factor (PERF) Study is based on subjects who were screened for or enrolled into RCT to identify genetic and other risk factors of diseases in the elderly	[PMID: 17109061] (Bagger, 2006 Links between cardiovascular disease and osteoporosis in postmenopausal women: serum lipids or atherosclerosis per se?)
**SLO-PREVAL**	*de-novo*	Prevalence of osteoporosis in Slovenia	Cross-sectional	Slovenia	Ljubljana	Central European	General Population	716	SLO-PREVAL study is a cross-sectional study where premenopausal women aged between 35-50 years and postmenopausal women and men aged over 50 years were included to perform genotype-phenotype association studies.	[PMID:12213850] (Arko, 2002 Sequence variations in the osteoprotegerin gene promoter in patients with postmenopausal osteoporosis); [PMID:18502820] (Mencej, 2008 Tumour necrosis factor superfamily member 11 gene promoter polymorphisms modulate promoter activity and influence bone mineral density in postmenopausal women with osteoporosis); [PMID:19781675] (Trošt, 2010 A microarray based identification of osteoporosis-related genes in primary culture of human osteoblasts)

**TWINGENE**	*in silico*	TwinGene	Cohort	Sweden	Stockholm	Northern European	General population - Family based	6347	In the TwinGene project twins born before 1958 are contacted to participate. Health and medication data are collected from self-reported questionnaires, and blood sampling material is mailed to the subject who then contacts a local health care center for blood sampling and a health check-up. In the simple health check-up, height, weight, circumference of waist and hip, and blood pressure are measured. The follow-up data were linked to the baseline data by the personal identification numbers assigned to every resident. Fractures in the cohort were identified through linkage to the national Swedish Patient Register by use of the individual personal registration number provided to all citizens and by a comprehensive computer-assisted telephone interview conducted between 1998 and 2002.	[PMID: 16157825] (Michaëlsson, 2005 Genetic liability to fractures in the elderly); [PMID: 23137839] (Magnusson, 2013 The Swedish Twin Registry: establishment of a biobank and other recent developments)
**UFO-HIP**	*in silico*	The Umeå Fracture and Osteoporosis Study - Hip Fractures	Nested case-cohort	Sweden	Umeå	Northern European	General population	1876	The UFO study is a nested case-control study investigating associations between genes, lifestyle and osteoporotic fractures. The study is based on the prosepctive and populationbased Northern Sweden Health and Disease Study cohort, initiated to assess risk factors for diabetes and cardiovascular disesase. Genome-wide association study data was generated for the hip fracture cases and matched controls.	[PMID: 20464545] (Englund, 2010 Physical activity in middle-aged women and hip fracture risk: the UFO study); [PMID:14660243] (Hallmans, 2003 Cardiovascular disease and diabetes in the Northern Sweden Health and Disease Study Cohort - evaluation of risk factors and their interactions)
**UFO-WRIST**	*in silico*	The Umeå Fracture and Osteoporosis Study - Wrist Fractures	Nested case-cohort	Sweden	Umeå	Northern European	General population	2115	The UFO study is a nested case-control study investigating associations between genes, lifestyle and osteoporotic fractures. The study is based on the prosepctive and populationbased Northern Sweden Health and Disease Study cohort, initiated to assess risk factors for diabetes and cardiovascular disesase. Genome-wide association study data was generated for the wrist fracture cases and matched controls.	[PMID: 20464545] (Englund, 2010 Physical activity in middle-aged women and hip fracture risk: the UFO study); [PMID:14660243] (Hallmans, 2003 Cardiovascular disease and diabetes in the Northern Sweden Health and Disease Study Cohort - evaluation of risk factors and their interactions)

**UFO-2**	*in silico*	The Umeå Fracture and Osteoporosis Study - 2	Nested case-cohort	Sweden	Umeå	Northern European	General population	2022	The UFO study is a nested case-control study investigating associations between genes, lifestyle and osteoporotic fractures. The study is based on the prosepctive and populationbased Northern Sweden Health and Disease Study cohort, initiated to assess risk factors for diabetes and cardiovascular disesase.	[PMID: 20464545] (Englund, 2010 Physical activity in middle-aged women and hip fracture risk: the UFO study); [PMID:14660243] (Hallmans, 2003 Cardiovascular disease and diabetes in the Northern Sweden Health and Disease Study Cohort - evaluation of risk factors and their interactions)

Table S2A | Discovery study-specific descriptive statistics

Study	Trait	*Men* N	mean	sd	min	max	*Women* N	mean	sd	min	max
AGES	Age (yrs)	1351	76.5	5.3	67	94	1865	76.3	5.6	66	95
	Weight (kg)	1351	83.2	13.3	42	144.7	1865	70.5	13.3	37.2	128.3
	Height (cm)	1351	175.4	6.2	153.1	196.4	1865	160.9	5.8	139.3	182.6
AOGC	Age (yrs)	0	NA	NA	NA	NA	1951	69.6	8.6	48	86
	Weight (kg)	0	NA	NA	NA	NA	1941	70.1	16.3	34.5	136
	Height (cm)	0	NA	NA	NA	NA	1937	159.7	6.8	127.5	188
BPROOF	Age (yrs)	1085	73.6	6.2	64	98	1113	74.9	6.8	63	97
	Weight (kg)	1085	83.1	11.8	48	180	1102	72.9	12.7	37	135
	Height (cm)	1085	176	6.5	158.2	197.5	1101	162.7	6.5	138.7	189
CHS	Age (yrs)	1276	73.5	5.9	65	95	1985	73.0	5.9	65	101
	Weight (kg)	1276	79.1	11.9	49.7	129.7	1985	66.1	13.2	33.3	132.9
	Height (cm)	1276	173.3	6.5	151	192.5	1985	159.0	6.1	124	178
DeCODE	Age (yrs)	1136	66.1	14.2	20.1	96.1	6469	59.7	13.8	20	97.8
	Weight (kg)	1136	83.4	14.4	38.4	129.5	6461	71.1	13.4	30	129.3
	Height (cm)	1136	176.5	6.7	148.5	196	6469	164.4	6.2	116.5	188
EGCUT-I	Age (yrs)	2254	54.8	19.3	18	100	2258	55.9	21.1	18	103
	Weight (kg)	2254	84.3	16.1	46	153	2258	72.3	15.8	28	145
	Height (cm)	2254	177	7.3	154	206	2258	163.3	7	140	193
EGCUT-II	Age (yrs)	875	40	16.6	18	90	912	40.5	15.6	18	91
	Weight (kg)	875	84.1	15.3	50	191	912	70.8	16.1	39	160
	Height (cm)	875	179.6	7.2	158	204	912	165.3	6.3	144	184
EPICNOR	Age (yrs)	9640	59.6	9.2	39.5	79.1	11023	58.7	9.3	39.8	78.2
	Weight (kg)	9635	80.3	11.3	42.8	160.0	11015	67.8	11.6	36.0	139.2
	Height (cm)	9631	174.0	6.6	139.4	200.2	11013	161.1	6.2	129.0	185.3

ERF	Age (yrs)	616	49.95	14.8	18.07	86.5	986	48.13	14.43	18.08	86.07
	Weight (kg)	616	83.05	14.4	50.6	154.7	986	69.2	13.8	42.1	151.8
	Height (cm)	616	174.5	7.3	152.2	196	986	161.7	6.6	141	182.8
FHS	Age (yrs)	1548	64.5	10.9	35	92	2081	64.9	11.5	29	96
	Weight (kg)	1544	86	14.9	46.3	170.1	2066	69.8	15	36.3	158.8
	Height (cm)	1540	174	7	152	200	2060	160	7	138	183
GOOD	Age (yrs)	938	18.9	0.6	18	20.1	0	NA	NA	NA	NA
	Weight (kg)	938	73.9	11.6	51.3	127	0	NA	NA	NA	NA
	Height (cm)	938	182	7	161	203	0	NA	NA	NA	NA
HABC	Age (yrs)	879	73.9	2.9	69	80	784	73.6	2.8	69	80
	Weight (kg)	879	81.6	12.4	52.2	134.5	784	66.4	12.1	40.8	123
	Height (cm)	879	173.6	6.4	151.1	194.8	784	159.4	5.8	141.6	175.6
HKOS	Age (yrs)	0	NA	NA	NA	NA	800	48.9	15.6	20	84
	Weight (kg)	0	NA	NA	NA	NA	800	54.7	10.2	33.5	93.5
	Height (cm)	0	NA	NA	NA	NA	800	155	6.7	127	175
MROS	Age (yrs)	5108	73.9	5.9	64	100	0	NA	NA	NA	NA
	Weight (kg)	5108	83.6	13.1	50.8	144.1	0	NA	NA	NA	NA
	Height (cm)	5108	174.5	6.6	147.2	198.9	0	NA	NA	NA	NA
PROSPER	Age (yrs)	2524	75	3.3	70.2	83.3	2718	75.7	3.4	69.4	83.4
	Weight (kg)	2524	78.7	11.9	40	127	2718	68.3	12.7	35.5	138
	Height (cm)	2524	172.1	6.7	143	198	2718	158.8	6.6	135	180
RS-I	Age (yrs)	2427	68.1	8.2	55	97.8	3547	70.3	9.6	55	99.2
	Weight (kg)	2375	78.6	10.7	41	122.3	3383	69.6	11.3	40.1	146.5
	Height (cm)	2372	174.8	6.8	151	198	3375	161.3	6.6	101	191.5
RS-II	Age (yrs)	785	63.7	6.8	55.1	89.3	902	63.8	7.4	55.1	92.3
	Weight (kg)	785	83.5	11.4	54	126.8	902	72.8	12.5	44.1	125.3
	Height (cm)	785	176	6.5	156.8	203	902	162.9	6.2	141.5	189.6
RS-III	Age (yrs)	528	56.1	5.5	45.9	84.2	683	56.1	5.4	45.8	87.9
	Weight (kg)	525	89.7	14.1	60.8	149.9	683	75.2	14.3	35	137.6

	Height (cm)	525	178.8	6.7	160.5	197.5	683	165	6.2	146.5	184.5
SOF	Age (yrs)	0	NA	NA	NA	NA	3299	71.5	5.2	65	89
	Weight (kg)	0	NA	NA	NA	NA	3260	67.8	11.9	40.8	112
	Height (cm)	0	NA	NA	NA	NA	3281	159.4	5.8	142.5	175.5
TUK123	Age (yrs)	373	49.8	14.6	18.3	81.4	3962	49.9	13.5	16.2	82.1
	Weight (kg)	373	80.4	11.8	40.5	122.7	3962	67.5	12.9	35.1	166
	Height (cm)	365	175.1	7.1	161	191	3882	162.2	6.3	148	177
UKBB	Age (yrs)	68105	57.29	8.12	40	73	76949	56.62	7.93	39	73
	Weight (kg)	68105	86.3	14.53	40.8	197.1	76949	71.66	14.2	32.1	196
	Height (cm)	68105	175.6	6.77	122	205	76949	162.5	6.22	126	192
WGHS	Age (yrs)	NA	NA	NA	NA	NA	22330	54.1	7.1	38	89
	Weight (kg)	NA	NA	NA	NA	NA	22330	70	14.2	38.6	175.1
	Height (cm)	NA	NA	NA	NA	NA	22330	164	6	13	201
WHICT	Age (yrs)	NA	NA	NA	NA	NA	1705	69	6.4	50	79
	Weight (kg)	NA	NA	NA	NA	NA	1705	72.6	14.5	40	171.5
	Height (cm)	NA	NA	NA	NA	NA	1705	161.5	6.3	137	183.4
WHIOS	Age (yrs)	NA	NA	NA	NA	NA	2592	69	6.5	50	79
	Weight (kg)	NA	NA	NA	NA	NA	2592	70.4	14.9	37.5	171.5
	Height (cm)	NA	NA	NA	NA	NA	2592	161.6	6.5	116	183.4
YFS	Age (yrs)	699	38	5	30	45	887	38	5	30	45
	Weight (kg)	699	86	15.4	54	166	887	69.9	13.9	42	166
	Height (cm)	699	179.5	6.6	157	203	887	166.3	5.9	147	189

Table S2B | Replication study-specific descriptive statistics

Study	Trait	*Men*					*Women*				
		**N**	**mean**	**sd**	**min**	**max**	**N**	**mean**	**sd**	**min**	**max**
23andMe	Age (yrs)	141571	53.5	16.7	1	117	155714	53.2	16	1	117
	Weight (kg)	NA	NA	NA	NA	NA	NA	NA	NA	NA	NA
	Height (cm)	NA	NA	NA	NA	NA	NA	NA	NA	NA	NA
AOGC-GOS	Age (yrs)	1322	59.3	17.9	20	94	1600	54.3	19.6	20.3	95.5
	Weight (kg)	1319	82.8	14.4	41.8	154.7	1598	68.8	14.4	35.3	138.9
	Height (cm)	1321	174.2	7.2	153.6	201	1598	160.5	7	132.3	186
AOGC-SHEFFIELD	Age (yrs)	0	NA	NA	NA	NA	3979	80.1	4	74.3	100
	Weight (kg)	0	NA	NA	NA	NA	4008	65.1	11.3	35.8	116.3
	Height (cm)	0	NA	NA	NA	NA	3971	155.9	6	134.3	178
APOSS	Age (yrs)	0	NA	NA	NA	NA	3268	48.5	2.4	44.2	56.3
	Weight (kg)	0	NA	NA	NA	NA	3264	66	12	40	146
	Height (cm)	0	NA	NA	NA	NA	3264	161.3	5.9	136	185
AROS	Age (yrs)	176	54.3	15.7	19	85	621	61.8	12.9	20	87
	Weight (kg)	145	78.1	12.4	54.5	119	551	63.8	11	38.6	118.4
	Height (cm)	144	176.2	7.4	158	203	548	161.7	6.7	143	194
AUSTRIOS-A	Age (yrs)	271	56.6	12	22	77	534	47.1	15.8	18	85
	Weight (kg)	268	83.4	12.1	58	125	496	63.9	10.6	36	106
	Height (cm)	268	176.7	6.6	160	197	496	164.2	6.3	148	183
AUSTRIOS-B	Age (yrs)	327	84.3	5.7	69	101	1737	83.9	6.2	68	103
	Weight (kg)	314	68.2	12.5	39	113	1648	60.4	12.2	31	111
	Height (cm)	312	164.9	8.2	140	185	1645	153.3	7.4	125	198
BARCOS	Age (yrs)	0	NA	NA	NA	NA	1451	65.5	9.1	35	100
	Weight (kg)	0	NA	NA	NA	NA	1443	65	10.5	41	134

	Height (cm)	0	NA	NA	NA	NA	1442	156.8	6.3	135	180
CABRIO-C	Age (yrs)	543	63.9	8.5	50	92	907	62	9.8	42	94
	Weight (kg)	543	81.5	11	44.5	118.4	887	68.7	12	42	119
	Height (cm)	543	168.2	6.1	150	189	889	155.9	6	138	188
CABRIO-CC	Age (yrs)	538	73.3	11.9	39	100	1771	74.5	12.3	43	104
	Weight (kg)	326	78.1	11.4	46	137	995	65	10.4	36	110
	Height (cm)	326	166.7	6.8	145	192	992	154.7	6.1	138	178
CAIFOS	Age (yrs)	0	NA	NA	NA	NA	1347	80.2	2.7	75	87
	Weight (kg)	0	NA	NA	NA	NA	1135	67.6	12.1	39.6	114.4
	Height (cm)	0	NA	NA	NA	NA	1137	157.5	6	115	178
CALEX	Age (yrs)	190	55.2	15.1	19.9	87.3	457	46	19	18	91.7
	Weight (kg)	190	81.4	10.7	56.8	108.6	457	68.1	12.3	46.1	127
	Height (cm)	190	175.9	6.5	154	194	457	164.2	6.2	148	180
CAMOS	Age (yrs)	705	65.4	16.6	18	95	1616	67.3	14.9	18	99
	Weight (kg)	701	81.7	13.4	47.7	126.5	1601	69.4	13.7	38.2	158.5
	Height (cm)	701	174.1	7.1	151	198	1600	160.6	6.4	141	182.9
DOES	Age (yrs)	569	75.6	5.5	61	90	888	76.2	6.3	60	99
	Weight (kg)	569	78.4	13.2	43	128	888	65.2	13.1	33	128
	Height (cm)	569	171.3	6.3	154	196	888	157.8	6.1	139	186
DOPS	Age (yrs)	0	NA	NA	NA	NA	1716	50.6	2.8	43.7	59
	Weight (kg)	0	NA	NA	NA	NA	1715	67.7	11.8	34	135.5
	Height (cm)	0	NA	NA	NA	NA	1715	164.5	6	147	189
EDOS-EPICNOR	Age (yrs)	3253	60	10	17.2	93.1	5498	63	11.2	20.4	99.5
	Weight (kg)	3234	80	12.5	40.6	144.8	5450	66.2	12.3	40	136.5
	Height (cm)	3237	173.6	7	101	200	5480	159.6	6.8	102	190
EPOLOS	Age (yrs)	317	50.4	16.5	19.8	80.9	398	55.5	15.6	20	81.6
	Weight (kg)	317	80	12.7	48	120	397	68	12.3	40	109

	Height (cm)	317	173.3	7.4	151	190	398	159.6	6.4	128	176
EPOS	Age (yrs)	719	62.9	8.2	43.9	90.4	1373	63.4	8.8	40.5	95
	Weight (kg)	661	78.7	11.5	47	120	1177	68.1	11.5	41.3	115
	Height (cm)	661	168.9	7.3	145	188	1179	157.4	6.9	136	186
FLOS	Age (yrs)	161	53.9	14.7	20	78	839	60.9	12	19	89
	Weight (kg)	161	80.8	13.6	60	185	839	61.8	9.3	37	116
	Height (cm)	161	175.6	7	156	192	839	160	6.7	138	181
GEOS	Age (yrs)	0	NA	NA	NA	NA	2379	53.8	9.6	18	84
	Weight (kg)	0	NA	NA	NA	NA	2374	65	11.9	40.2	118.8
	Height (cm)	0	NA	NA	NA	NA	2377	158.6	6	130	184
GEVUR	Age (yrs)	134	59.2	12.9	19	83	839	62.2	8.2	40	85
	Weight (kg)	96	76.2	14	49	120	830	70.6	13.2	40	128
	Height (cm)	97	170.8	7.5	156	192	833	159.3	6.3	134	185
GEVUR-2	Age (yrs)	402	60.6	12	31	87	11	58	9.6	40	71
	Weight (kg)	402	78.1	14.7	45	133	11	67.8	10.7	50	83
	Height (cm)	402	168.1	7.6	138	192	11	160.5	4.9	154	168
GROS	Age (yrs)	83	70.2	12.8	43	90	523	69.1	11.7	43	95
	Weight (kg)	83	71.5	12.3	40	120	521	71.7	10.9	40	112
	Height (cm)	83	164.8	10.4	121	182	523	161.8	7.3	146	182
HCS	Age (yrs)	1571	65.7	2.9	59.2	72.6	1356	66.7	2.7	60.9	73.1
	Weight (kg)	1563	82.4	12.6	45.5	144.5	1354	71.2	13.2	40	135.5
	Height (cm)	1564	174.1	6.5	149.6	195.6	1354	160.9	5.9	140.9	180.8
HKOS	Age (yrs)	1888	72.4	5	65	92	1984	72.6	5.4	65	98
	Weight (kg)	1888	62.4	9.4	35.9	103.1	1984	54.5	8.5	28.8	89
	Height (cm)	1888	163.1	5.8	141.9	187.2	1984	150.9	5.3	133.7	170.3
KORAMC	Age (yrs)	0	NA	NA	NA	NA	1397	59.5	7.4	45	87
	Weight (kg)	0	NA	NA	NA	NA	1397	56.2	7.2	27	86

	Height (cm)	0	NA	NA	NA	NA	1397	155	5.3	121	171
LASA	Age (yrs)	464	72.4	6.5	61.9	85.6	485	72.6	6.6	61.8	85.3
	Weight (kg)	440	77.9	11.3	47	119	449	71.6	12	42	120.5
	Height (cm)	438	173.4	6.7	156.4	195.3	442	160.5	6.3	141.9	177.6
MANMC	Age (yrs)	0	NA	NA	NA	NA	1105	56.4	8.6	27	86
	Weight (kg)	0	NA	NA	NA	NA	0	NA	NA	NA	NA
	Height (cm)	0	NA	NA	NA	NA	0	NA	NA	NA	NA
MINOS	Age (yrs)	794	65.4	7.3	51	85	0	NA	NA	NA	NA
	Weight (kg)	794	80.1	12.7	51	140	0	NA	NA	NA	NA
	Height (cm)	794	169	6.3	147	188	0	NA	NA	NA	NA
MOFS	Age (yrs)	0	NA	NA	NA	NA	1046	64.4	8.9	40	79
	Weight (kg)	0	NA	NA	NA	NA	1046	66.9	12.4	42	141
	Height (cm)	0	NA	NA	NA	NA	1046	154	6.2	134	178
MROSS	Age (yrs)	2922	75.4	3.2	69.9	81	0	NA	NA	NA	NA
	Weight (kg)	2922	80.7	12.1	37	138.3	0	NA	NA	NA	NA
	Height (cm)	2922	174.8	6.5	145.2	199.4	0	NA	NA	NA	NA
NOSOS	Age (yrs)	0	NA	NA	NA	NA	1,268	69.7	5.5	60.2	82.2
	Weight (kg)	0	NA	NA	NA	NA	1,291	67.8	12.3	40	132.5
	Height (cm)	0	NA	NA	NA	NA	1,293	158.4	6	131	177
OAS	Age (yrs)	600	68.1	4.2	60	76	0	NA	NA	NA	NA
	Weight (kg)	593	83.7	12.5	48	137.9	0	NA	NA	NA	NA
	Height (cm)	593	174.3	6.7	128.4	191.8	0	NA	NA	NA	NA
OPRA	Age (yrs)	0	NA	NA	NA	NA	1044	75.2	0.2	75	76
	Weight (kg)	0	NA	NA	NA	NA	1042	67.8	11.6	41	110
	Height (cm)	0	NA	NA	NA	NA	1024	160.5	5.7	140	180
OSTEOS-II	Age (yrs)	53	48.1	16.5	21	73	510	51.7	12.3	20	85
	Weight (kg)	53	81.7	16.4	48.8	136.6	510	72.3	14.4	42.6	126.8

	Height (cm)	53	173.2	7.5	154	185	510	161.1	6.4	142	185
PERF	Age (yrs)	0	NA	NA	NA	NA	3927	64.1	7.9	45.2	80.8
	Weight (kg)	0	NA	NA	NA	NA	3973	66.1	9.7	36.1	116.6
	Height (cm)	0	NA	NA	NA	NA	3973	161.8	6	134.3	190
SLO-PREVAL	Age (yrs)	123	67.9	6.5	55	89	593	62.1	10.6	38	93
	Weight (kg)	123	81.6	12.6	55	130	593	69.1	12.2	45	115
	Height (cm)	123	171.7	6.3	159	192	593	160.4	6.3	137	181
TWINGENE	Age (yrs)	4432	65.6	8.04	47.6	93.3	4948	64.7	8.22	47.4	93.9
	Weight (kg)	4349	81.9	12.22	50	179.1	4827	68.8	11.96	37.6	171.5
	Height (cm)	4432	180	7	140	210	4948	160	6	140	190
UFO-HIP	Age (yrs)	807	60.4	12.1	20	91	1069	64.7	11.1	21	90
	Weight (kg)	794	81.5	12.1	56	135	1037	67.9	12.6	40	132
	Height (cm)	795	176.7	6.7	130	197	1035	163.4	6	144	184
UFO-WRIST	Age (yrs)	306	59	7.3	31.8	75.9	1809	61.6	6.7	39.2	80.1
	Weight (kg)	305	83	13.1	56.5	163.2	1706	67.9	10.9	33	112
	Height (cm)	305	176.6	5.8	160	192	1706	163.7	5.8	142	191
UFO-2	Age (yrs)	421	48.2	10.3	24	70	1601	53.3	10.2	19	80
	Weight (kg)	421	82.1	11.4	51	129	1601	68.1	12.1	36	127
	Height (cm)	421	177.9	6.1	152	198	1601	164	6.1	130	190

Table S3A | Discovery study-specific fracture counts

Study	Fracture Assessment Method	Fracture N	Non-Fracture N	Total
AGES	Medical and radiographic records	1,458	1,727	3,185
AOGC	Questionnaire, radiography	685	1,113	1,798
BPROOF	History of fractures before baseline: questionnaire. Incident fractures: self-report, validated at GP/hospital	715	1,483	2,198
CHS	Self-report of incident fracture of the hip, leg, arm, or vertebra	519	2,742	3,261
DeCODE	Medical records, radiographic documentation, questionnaire	1,836	14,560	16,396
EGCUT-I	Medical records, questionnaire	217	4,296	4,513
EGCUT-II	Medical records, questionnaire	71	1,717	1,788
EPICNOR	Medical records	2,926	17,710	20,636
ERF	Interview	260	1,342	1,602
FHS	Medical records, questionnaire	1,520	2,782	4,302
GOOD	Radiographic document	273	597	870
HEALTHABC	Radiographic	308	1,353	1,661
HKOS	Medical records, Radiographic and Questionnaire	79	627	706
MROS	Questionnaire, radiographic documentation	918	3,555	4,473
PROSPER	Medical records	426	4,816	5,242
RS-I	Medical records, questionnaire	2,163	3,574	5,737
RS-II	Medical records, questionnaire	932	1,220	2,152
RS-III	Medical records, questionnaire	505	2,421	2,926
SOF	Questionnaire, radiographic documentation	1,611	1,698	3,309
TUK123	Medical records, Radiographic and Questionnaire	839	4,111	4,950
UKBB	Questionnaire, based on answering yes to the question “Have you fractured/broken any bones in the last 5 years?” at either baseline or first follow-up.	14,492	130,563	145,055
WGHS	Questionnaire	1,832	20,498	22,330
WHICT	Medical records	1,058	647	1,705
WHIOS	Medical records	1,603	989	2,592
YFS	Medical records	611	975	1,586
**Total**		**37,857**	**227,116**	**264,973**

Table 3B | Replication study-specific fracture counts

Study	Fracture Assessment Method	Fracture N	Non-Fracture N	Total
23andMe	Questionnaire	147,200	150,085	297,285
AOGC-GOS	Questionnaire, radiography	574	1,763	2,337
AOGC-SHEFFIELD	Questionnaire, radiography lateral morphometry	1,373	2,013	3,386
APOSS	Self-reported	560	2,275	2,835
AROS	Radiographic	335	130	465
AUSTRIOS-A	Medical records, partly radiographic documentation	234	503	737
AUSTRIOS-B	Medical records, partly radiographic documentation	960	1,104	2,064
BARCOS	Medical records, radiographic documentation	179	1,258	1,437
CABRIO-C	Patient-referred/Medical records	341	1,109	1,450
CABRIO-CC	Patient-referred/Medical records	1,122	1,193	2,315
CAIFOS	Self-reported xray report verified	749	598	1,347
CALEX	First by questionnaire, then confirmed by medical records, radiographic documentation	113	411	524
CAMOS	Medical records, radiographic documentation	410	1785	2,195
DOES	X-ray reports	546	835	1,381
DOPS	Radiographic	425	1,291	1,716
EDOS	Medical records, radiographic documentation	1626	149	1,775
EPICNOR	Prevalent fractures were self-reported on questionnaire and (where possible) were confirmed by review of medical records and radiographic documentation.	1,647	2004	3,651
EPOLOS	Self -reports (fractures data from questionnaire filled in by physician)	248	467	715
EPOS	Prevalent limb fractures were self-reported on questionnaire and (where possible) were confirmed by review of medical records and radiographic documentation. Prevalent vertebral fractures confirmed on baseline radiograph.	564	1,168	1,732
FLOS	Medical records, radiographic documentation	144	577	721
GEOS	110 cases reported by the patient. No medical reports.	110	1,799	1,909
GEVUR	Medical records, radiographic documentation	407	592	999
GEVUR-2	Medical records, radiographic documentation	263	147	410
GROS	Medical records, radiographic documentation	394	151	545
HCS	Self-report	356	2,435	2,791
HK	X-ray films or radiology reports, medical record	794	3,078	3,872
KORAMC	Self-report and radiographic	171	1,226	1,397
LASA	Self-report (fracture calendar/interview), GP questionnaire (FX verified at GP or hospital)	326	623	949
MANMC	Medical records, Surgical Report, ICD-9 Codes	848	0	848

MINOS	Questionnaire and Radiographic	66	429	495
MOFS	Medical records, radiographic documentation	262	764	1,026
MROSS	Prevalent: Questionnaire, Incident: Radiographic documentation	1255	1,651	2,906
NOSOS	Self-report	385	843	1,228
OAS	Radiographic	97	503	600
OPRA	Medical records	493	479	972
OSTEOS-II	Medical records	100	447	547
PERF	Medical records, radiographic documentation	1,051	2,405	3,456
SLO-PREVAL	Medical records	91	172	263
TWINGENE	Medical records, questionnaire	2,045	4,302	6,347
UFO-HIP	Medical records, radiographic documentation	1,014	862	1,876
UFO-WRIST	Medical records, radiographic documentation	1,060	1,055	2,115
UFO-2	Medical records, radiographic documentation	191	1,831	2,022
**Total**		**171,129**	**196,512**	**367,641**

Table S4A | Genotyping/Imputation discovery

	Genotyping						Imputation*			Association Analyses			
			Inclusion Criteria					Inclusion Criteria					
Cohort	Platform	Genotype Calling Algorithm	MAF	Call Rate*	P for HWE	SNPs Post-QC	Imputation Software	MAF	Imputation Quality*	Analysis Software	No. analyzed SNPs	No. filtered SNPs	λ
**AGES**	Illumina Hu370CNV	BEadstudio Genecall	≥ 1%	>97%	$> 10^{-6}$	329,804	MACH	≥1%	MACH R2 ≥ 0.3	Prob-ABEL	2,409,007	6	1.02
**AOGC**	Illumina Infinium II 370CNVQuad (n=1882); HumHap300 (n=140), 370CNVDuo (n=4) and 610Quad (n=10)	BeadStudio	≥ 1%	≥ 98%	$> 10^{-7}$	289,499	MACH	≥ 1%	MACH R2 ≥ 0.3	MACH-2DAT	2,407,019	384	1.02
**BPROOF**	Illumina Omni-express	Beadstudio Genecall	≥ 1%	≥ 97.5%	$> 10^{-6}$	572,784	MACH	≥ 1%	MACH R2 ≥ 0.3	MACH-2DAT via GRIMP	2,450,941	59	1.01
**CHS**	Illumina 370CNV	BeadStudio	> 1%	≥97%	$> 10^{-5}$	306,655	BimBam	≥1%	$(O/E)\sigma^2$ ratio ≥ 0.3	R	2,190,158	41	1.00
**DeCODE**	Illumina HH300 and 370CNV	BeadStudio	> 1%	> 96%	$> 10^{-6}$	281,410	IMPUTE	≥1%	Prop_info >0.4	SNP-TEST	2,399,139	0	1.13
**EGCUT-I**	Illumina HumanOmniExpress	Genome Studio	> 1%	> 95%	$> 10^{-6}$	617,595	IMPUTE	≥ 1%	Prop_info >0.4	SNP-TEST	2,434,283	379	1
**EGCUT-II**	Illumina HumanCNV370	Genome Studio	> 1%	> 95%	$> 10^{-6}$	320,784	IMPUTE	≥1%	Prop_info >0.4	SNP-TEST	2,417,274	6,905	1.01
**EPICNOR**	UK BioBank Axiom	Axiom GT1	Chip-specific criteria	≥ 95%	> 10-8	728,244	ShapeIT, IMPUTE	See keft	Prop_info >=0.4	SNP-TEST	32,390,434	3,820,949	1.04
**ERF**	Ilumina 318K, 370K, Afymetrix 250K	Beadstudio, BRLMM	> 1%	> 98%	$> 10^{-6}$	487,573	MACH	≥1%	MACH R2 ≥ 0.3	Prob-ABEL	2,408,722	2,209	1.09
**FHS**	Affymetrix 500K Dual GeneChip + 50K gene-centered MIP set	BRLMM	≥ 1%	≥ 97%	$\geq 10^{-6}$	378,163	MACH	≥1%	$(O/E)\sigma^2$ ratio ≥ 0.3	Kinship R-Package	2,411,851	0	1
**GOOD**	Illumina / HumanHap 610 Quad	Beadstudio Genecall	≥ 1%	≥ 98%	$> 10^{-6}$	521,160	MACH	≥1%	MACH R2 ≥ 0.3	MACH-2DAT via GRIMP	2,449,205	1,768	1.02
**HABC**	Illumina/ Human 1M-Duo	Beadstudio	≥1%	≥97%	$> 10^{-6}$	914,263	MACH	≥1%	MACH R2 ≥ 0.3	SNP-TEST	2,469,391	747	0.99
**HKOS**	Illumina / Human610-Quad	Illumina's GenomeStudio	≥ 1%	≥ 95%	$> 10^{-4}$	488,853	IMPUTE	≥1%	Prop_info >0.4	SNP-TEST	2,091,885	13,395	1.02

**MROS**	Illumina HumanOmni1_Quad_v1-0 B	BeadStudio	≥1%	≥97%	$> 10^{-4}$	740,713	MACH/ minimac	≥1%	MACH R2 ≥ 0.3	MACH-2DAT	2,460,836	4,365	1.01
**PROSPER/PHASE**	Illumina Beadchip 660K-quad	Beadstudio Genecall	≥ 1%	≥ 97.5%	$> 10^{-6}$	557,192	MACH	≥1%	MACH R2 ≥ 0.3	PLINK	2,448,014	270	1
**RS-I**	Illumina / HumanHap 550K V.3 /HumanHap 550 V.3 DUO;	Beadstudio Genecall	≥ 1%	≥ 97.5%	$> 10^{-6}$	512,349	MACH	≥1%	MACH R2 ≥ 0.3	MACH-2DAT via GRIMP	2,448,227	0	1.02
**RS-II**	Illumina / HumanHap 550K V.3 /HumanHap 550 V.3 DUO;	Beadstudio Genecall	≥ 1%	≥ 97.5%	$> 10^{-6}$	466,389	MACH	≥1%	MACH R2 ≥ 0.3	MACH-2DAT via GRIMP	2,447,128	5,051	1.00
**RS-III**	Illumina / HumanHap610	Beadstudio Genecall	≥ 1%	≥ 97.5%	$> 10^{-6}$	514,073	MACH	≥1%	MACH R2 ≥ 0.3	MACH-2DAT via GRIMP	2,447,902	166	1.03
**SOF**	Illumina HumanOmni1_Quad_v1-0 B	BeadStudio	≥1%	≥97%	$> 10^{-4}$	740,713	MACH/ minimac	≥1%	MACH R2 ≥ 0.3	MACH-2DAT	2,368,741	17	1
**TUK-1,2,3**	Illumina HumanHap 300 & 550. Illumina HumanCNV370 Duo. Illumina 610k	Beadstudio Genecall	≥ 1%	≥ 95%	$> 10^{-6}$	545,026	IMPUTE	≥1%	Prop_info >0.4	Gen-ABEL	2,376,877	26	1
**UKBB**	UK Biobank Axiom Array (N~450,000) and UK BiLEVE Array (N~50,000)	Affymetrix Power Tools software and the Affymetrix Best Practices Workflow	<1%			641,018	Phasing: Modified version of SHAPEIT2; Imputation: modified version of IMPUTE2	<1%	0.3	BOLT-LMM	72,355,668	54,968,115	
**WGHS**	Illumina/HumanHap300 Duo Plus	Beadstudio v3.3	> 1%	≥ 98%	$> 10^{-6}$	339,596	MACH	≥1%	MACH R2 ≥ 0.3	Prob-ABEL	2,435,874	0	1.00
**WHICT**	Illumina HumanHap 550K, Illumina HumanHap 610K	Beadstudio Gencall	≥ 1%	≥ 98%	$> 10^{-6}$	499,982	MACH	≥1%	MACH R2 ≥ 0.3	R	2,454,978	117	1.02
**WHIOS**	Illumina HumanHap 550K, Illumina HumanHap 610K	Beadstudio Gencall	≥ 1%	≥ 98%	$> 10^{-6}$	499,982	MACH	≥1%	MACH R2 ≥ 0.3	R	2,456,006	22	0.99
**YFS**	Illumina Custom BeadChip Human670K	Illumina	> 1%	> 95%	$> 10^{-6}$	546,677	MACH	≥1%	MACH R2 ≥ 0.3	Prob ABEL	2,424,091	135	1.02

*23 cohorts were imputed to HapMap release 22, EPIC was imputed to 1,000 genomes reference panel only and UKBB was imputed using UK10K consortium panel combined with the 1000 genomes reference panel

Table S4B | Genotyping GENOMOS replication

Study	Genotyping				
	Genotyping Centre	Genotyped SNPs	SNP Call Rate	SNPs Post-QC	Samples Post-QC
AOGC-GOS	AOGC	2	>90%	2	2,635
AOGC-SHEFFIELD	AOGC	2	>90%	2	3,817
APOSS	KBIO	2	>90%	2	3,066
AROS	KBIO	24	>90%	23	802
AUSTRIOS-A	KBIO	24	>90%	Failed	Failed
AUSTRIOS-B	KBIO	24	>90%	20	1,017
BARCOS	KBIO	24	>90%	21	1,437
CABRIO-C	KBIO	24	>90%	21	1,508
CABRIO-CC	KBIO	24	>90%	20	2,233
CAIFOS	KBIO	24	>90%	21	1,341
CALEX	KBIO	24	>90%	21	989
CAMOS	KBIO	24	>90%	20	2,384
DOES	DECODE	2	>90%	2	1,347
DOPS	KBIO	24	>90%	20	1,732
EDOS	KBIO	24	>90%	22	2,644
EPICNOR	KBIO	22	>90%	22	3,978
EPOLOS	KBIO	2	>90%	2	688
EPOS	KBIO	9	>90%	7	2,003
FLOS	KBIO	24	>90%	23	1,465
GEOS	KBIO	24	>90%	21	2,330
GEVUR	KBIO	24	>90%	23	1,035
GEVUR-2	KBIO	22	>90%	22	433
GROS	KBIO	24	>90%	21	491
HCS	KBIO	24	>90%	22	3,196
HKOS	KBIO/DECODE	24	>90%	24	3,884

KORAMC	KBIO/DECODE	24	>90%	23	1,397
LASA	KBIO	24	>90%	22	1,171
MANMC	KBIO	24	>90%	22	1,038
MINOS	KBIO	22	>90%	22	742
MOFS	KBIO	22	>90%	22	1,029
MROSS	KBIO	24	>90%	19	2,825
NOSOS	KBIO	2	>90%	2	1,191
OAS	KBIO	24	>90%	23	590
OPRA	KBIO	22	>90%	21	1,022
OSTEOS-II	KBIO	22	>90%	21	573
PERF	KBIO	2	>90%	2	3,346
SLO-PREVAL	KBIO	24	>90%	22	708
UFO-1	KBIO	24	>90%	23	2,033

Table S4C | Genotyping/Imputation in silico replication

Genotyping							Imputation			Association Analyses	
			Inclusion Criteria					Inclusion Criteria			
**Cohort**	**Platform**	**Genotype Calling Algorithm**	**MAF**	**Call Rate***	**HWE P-value**	**SNPs Post-QC**	**Imputation Software**	**MAF**	**Imputation Quality***	**Analysis Software**	**N SNPs Analysed**
23andMe							Minimac3		$R^2 \geq$ 0.5	C++	20
UFO-hip	Illumina 660 Quad Array	Beadstudio Genecall	≥ 1%	≥ 97.5%	> 10^{-6}	531,659	MACH	≥1%	MACH $R^2 \geq$ 0.3	MACH2DAT via GRIMP	49
UFO-wrist	Illumina Omni Express	Beadstudio Genecall	≥ 1%	≥ 97.5%	> 10^{-6}	663,853	MACH	≥1%	MACH $R^2 \geq$ 0.3	MACH2DAT via GRIMP	49
TwinGene	Illumina Omni-express 700k	GenomeStudio 2010.3	≥ 1%	≥ 97%	> 10^{-7}	644,556	IMPUTE2	≥1%	Prop_info ≥0.4	PLINK	47 (2,432,506)*

[a] complete sample size for secondary analyses

Table S5A | Number of SNPs and Variance explained by Instrumental Variables for Each Risk Factor

Disease or Trait	N SNPs	Variance Explained by SNPs	Reference
**Femoral Neck BMD**	**43**	5.8%	15
**Lumbar Spine BMD**	**40**	6.4%	15
**Age at Menopause**	**54**	6.0%	21
**Rheumatoid Arthritis***	30	5.5%*	31
**Type 1 Diabetes**	19	6.7%	32
**Inflammatory Bowel Disease****	151	7.5%	26
**Thyroid-Stimulating Hormone**	20	5.6%	25
**Grip Strength**	15	1.4%	22
**Age of Puberty**	106	2.7%	20
**Fasting Glucose**	35	6.0%	27,28
**Coronary Heart Disease**	38	5.7%	33
**Type 2 Diabetes**	38	5.7%	30
**Vitamin D**	4	2.4%	23,24
**Milk Calcium Intake**	1	n/a	69

*The variance explained in rheumatoid arthritis by these SNPs is at least 5.5%, as this estimate excludes the MHC locus.
**The variance explained by SNPs for IBD is based on ulcerative colitis. The variance explained for Crohn's disease is 13.6%.

Table S5B | Individual summary statistics from the SNP-Exposure/Fracture association

					Summary Statistics from SNP-Exposure Associations			Summary Statistic for SNP-Fracture Associations		
Trait	**SNP**	**EA**	**NEA**	**EAF**	**Beta**	**se**	**p-value**	**Beta**	**se**	**p-value**
FN BMD										
	rs10048146	G	A	0.80	-0.056	0.011	$1.0x10^{-14}$	0.022	0.010	0.04
	rs1026364	G	T	0.37	-0.041	0.009	$4.1x10^{-10}$	-0.009	0.009	0.32
	rs1053051	T	C	0.48	-0.036	0.008	$9.6x10^{-10}$	0.021	0.008	0.01
	rs11623869	T	G	0.65	-0.041	0.009	$5.2x10^{-16}$	0.026	0.009	$2.7x10^{-3}$
	rs12407028	C	T	0.60	-0.048	0.008	$3.4x10^{-23}$	0.022	0.008	0.01
	rs1286083	T	C	0.19	-0.059	0.011	2.0x10-^{15}	0.042	0.011	$7.9x10^{-5}$
	rs13336428	A	G	0.57	-0.043	0.008	$1.5x10^{-16}$	-0.009	0.008	0.28
	rs1346004	A	G	0.50	-0.052	0.008	$1.1x10^{-25}$	-0.015	0.008	0.07
	rs1366594	C	A	0.54	-0.092	0.008	$4.5x10^{-61}$	0.023	0.008	0.01
	rs1373004	T	G	0.87	-0.055	0.013	$1.5x10^{-8}$	0.084	0.013	$1.1x10^{-10}$
	rs1566045	T	C	0.20	-0.074	0.011	$1.9x10^{-22}$	0.031	0.011	0.01
	rs163879	T	C	0.32	-0.042	0.009	$2.1x10^{-8}$	0.031	0.009	$4.6x10^{-4}$
	rs17040773	C	A	0.76	-0.045	0.010	$1.5x10^{-9}$	0.020	0.010	0.05
	rs2016266	A	G	0.32	-0.045	0.009	$3.7x10^{-10}$	0.024	0.009	$5.0x10^{-3}$
	rs2062377	A	T	0.43	-0.063	0.008	$9.1x10^{-25}$	-0.007	0.008	0.40
	rs227584	A	C	0.30	-0.060	0.009	$2.6x10^{-24}$	0.041	0.009	4.9x10-^{6}

rs2887571	A	G	0.24	-0.037	0.010	$6.5x10^{-9}$	0.024	0.010	0.01
rs3736228	T	C	0.84	-0.054	0.011	$4.8x10^{-11}$	0.047	0.011	$3.7x10^{-5}$
rs3755955	A	G	0.84	-0.061	0.012	$1.5x10^{-14}$	0.047	0.012	$4.7x10^{-5}$
rs3790160	C	T	0.50	-0.043	0.008	$3.6x10^{-12}$	0.004	0.008	0.65
rs3801387	A	G	0.26	-0.071	0.009	$5.0x10^{-40}$	0.062	0.009	$1.7x10^{-11}$
rs430727	T	C	0.52	-0.074	0.009	$4.4x10^{-25}$	0.032	0.008	$1.2x10^{-4}$
rs4727338	G	C	0.67	-0.081	0.009	$8.1x10^{-48}$	0.051	0.009	$4.4x10^{-9}$
rs4790881	C	A	0.69	-0.051	0.009	$9.8x10^{-19}$	0.021	0.009	0.02
rs479336	T	G	0.26	-0.050	0.009	$8.5x10^{-15}$	0.003	0.009	0.75
rs4796995	G	A	0.63	-0.040	0.009	$4.9x10^{-8}$	0.060	0.008	$1.9x10^{-12}$
rs4869742	T	C	0.69	-0.068	0.009	$4.2x10^{-18}$	0.043	0.009	$1.9x10^{-6}$
rs4985155	A	G	0.33	-0.031	0.009	$1.7x10^{-10}$	0.005	0.009	0.55
rs6426749	G	C	0.17	-0.108	0.011	$7.4x10^{-57}$	0.052	0.011	$1.9x10^{-6}$
rs6532023	G	T	0.34	-0.051	0.009	$5.0x10^{-26}$	0.012	0.009	0.18
rs6959212	T	C	0.68	-0.030	0.009	$1.2x10^{-13}$	0.039	0.009	$6.4x10^{-6}$
rs7084921	C	T	0.39	-0.032	0.008	$9.0x10^{-10}$	-0.004	0.008	0.60
rs7108738	T	G	0.17	-0.093	0.011	$1.1x10^{-32}$	0.026	0.011	0.02
rs7217932	G	A	0.46	-0.045	0.008	$1.9x10^{-11}$	0.021	0.008	0.01
rs7584262	C	T	0.23	-0.053	0.010	$1.3x10^{-9}$	0.023	0.010	0.02
rs7812088	G	A	0.13	-0.061	0.013	$7.3x10^{-9}$	0.035	0.013	$4.9x10^{-3}$
rs7851693	G	A	0.64	-0.047	0.009	$3.4x10^{-22}$	0.034	0.009	$1.3x10^{-4}$

	rs7932354	C	T	0.31	-0.050	0.009	5.1x10^{-18}	0.029	0.009	1.2x10^{-3}
	rs7953528	T	A	0.18	-0.058	0.011	1.9x10^{-12}	0.007	0.011	0.53
	rs884205	A	C	0.73	-0.042	0.010	3.2x10^{-10}	0.007	0.010	0.49
	rs9466056	A	G	0.62	-0.048	0.008	2.7x10^{-13}	0.020	0.008	0.02
	rs9533090	T	C	0.51	-0.054	0.008	4.9x10^{-23}	0.028	0.008	7.5x10^{-04}
	rs9921222	T	C	0.52	-0.043	0.008	5.2x10-^{12}	0.021	0.008	0.01
LS BMD										
	rs10048146	G	A	0.80	-0.061	0.011	3.1x10^{-11}	0.022	0.010	0.04
	rs10416218	T	C	0.27	-0.056	0.010	6.6x10^{-11}	0.012	0.009	0.21
	rs10835187	T	C	0.45	-0.036	0.009	4.9x10^{-8}	0.020	0.008	0.02
	rs11623869	T	G	0.65	-0.030	0.009	5.1x10^{-11}	0.026	0.009	0.003
	rs11755164	C	T	0.60	-0.052	0.010	5.6x10^{-11}	-0.014	0.009	0.09
	rs12407028	C	T	0.60	-0.081	0.009	3.1x10^{-45}	0.022	0.008	0.01
	rs12821008	C	T	0.39	-0.047	0.009	1.2x10^{-15}	0.011	0.008	0.19
	rs1286083	T	C	0.19	-0.074	0.011	1.8x10^{-14}	0.042	0.011	7.9x10^{-5}
	rs13204965	C	A	0.76	-0.044	0.012	3.6x10^{-10}	-0.013	0.010	0.20
	rs13336428	A	G	0.57	-0.036	0.009	1.7x10^{-13}	-0.009	0.008	0.28
	rs1346004	A	G	0.50	-0.049	0.009	3.9x10^{-30}	-0.015	0.008	0.07
	rs1373004	T	G	0.87	-0.073	0.014	1.6x10^{-12}	0.084	0.013	1.1x10^{-10}
	rs163879	T	C	0.32	-0.049	0.009	2.2x10^{-11}	0.031	0.009	4.6x10^{-4}
	rs1864325	T	C	0.78	-0.057	0.011	4.9x10^{-11}	0.014	0.010	0.18

rs1878526	G	A	0.22	-0.048	0.011	$1.2x10^{-10}$	-0.001	0.010	0.91
rs2016266	A	G	0.32	-0.063	0.009	$3.0x10^{-20}$	0.024	0.009	$4.96E^{-3}$
rs2062377	A	T	0.43	-0.081	0.009	$3.2x10^{-39}$	-0.007	0.008	0.40
rs227584	A	C	0.30	-0.048	0.010	$9.9x10^{-10}$	0.041	0.009	$4.9x10^{-6}$
rs2887571	A	G	0.24	-0.052	0.010	$5.6x10^{-12}$	0.024	0.010	0.01
rs344081	C	T	0.87	-0.057	0.014	$4.5x10^{-12}$	0.015	0.012	0.22
rs3736228	T	C	0.84	-0.081	0.012	$2.1x10^{-26}$	0.047	0.011	$3.71x10^{-5}$
rs3755955	A	G	0.84	-0.068	0.013	$5.2x10^{-15}$	0.047	0.012	$4.73x10^{-5}$
rs3790160	C	T	0.50	-0.057	0.009	$3.1x10^{-19}$	0.004	0.008	0.65
rs3801387	A	G	0.26	-0.083	0.010	$3.2x10^{-51}$	0.062	0.009	$1.7x10^{-11}$
rs3905706	C	T	0.22	-0.063	0.011	$2.4x10^{-16}$	-0.008	0.010	0.46
rs4233949	G	C	0.38	-0.062	0.009	$2.3x10^{-18}$	0.033	0.008	$7.0x10^{-5}$
rs430727	T	C	0.52	-0.056	0.009	$1.5x10^{-18}$	0.032	0.008	$1.2x10^{-4}$
rs4727338	G	C	0.67	-0.074	0.009	$2.1x10^{-35}$	0.051	0.009	$4.4x10^{-9}$
rs4792909	G	T	0.37	-0.052	0.009	$9.4x10^{-10}$	0.058	0.009	$7.8x10^{-12}$
rs4869742	T	C	0.69	-0.087	0.009	$4.0x10^{-35}$	0.043	0.009	$1.9x10-^{6}$
rs4985155	A	G	0.33	-0.045	0.009	$2.2x10^{-09}$	0.005	0.009	0.55
rs6426749	G	C	0.17	-0.105	0.011	$1.9x10^{-44}$	0.052	0.011	0.00
rs6532023	G	T	0.34	-0.061	0.009	$1.2x10^{-27}$	0.012	0.009	0.18
rs6959212	T	C	0.68	-0.077	0.009	$3.8x10^{-38}$	0.039	0.009	$6.4x10^{-6}$
rs7071206	T	C	0.22	-0.074	0.011	$5.0x10-^{19}$	-0.002	0.010	0.86

	rs7932354	C	T	0.31	-0.041	0.009	$5.5x10^{-12}$	0.029	0.009	0.001
	rs884205	A	C	0.73	-0.065	0.011	$1.6x10^{-17}$	0.007	0.010	0.49
	rs9466056	A	G	0.62	-0.036	0.009	$3.6x10^{-08}$	0.020	0.008	0.02
	rs9533090	T	C	0.51	-0.110	0.009	$4.8x10^{-68}$	0.028	0.008	$7.5x10^{-4}$
	rs9921222	T	C	0.52	-0.049	0.009	$1.0x10^{-16}$	0.021	0.008	0.01
Rheumatoid Arthritis										
	rs10175798	A	G	0.38	0.068	0.037	$4.2x10^{-8}$	-0.005	0.008	0.59
	rs10985070	C	A	0.47	0.068	0.037	$4.2x10^{-9}$	0.007	0.008	0.38
	rs11574914	A	G	0.35	0.113	0.039	$1.8x10^{-15}$	-0.001	0.009	0.95
	rs11889341	T	C	0.21	0.086	0.040	$1.4x10^{-12}$	0.001	0.010	0.92
	rs1516971	T	C	0.18	0.307	0.060	$3.2x10^{-11}$	-0.016	0.012	0.18
	rs1571878	C	T	0.41	0.148	0.038	$2.4x10^{-18}$	0.015	0.008	0.06
	rs17264332	G	A	0.18	0.113	0.048	$4.1x10^{-20}$	0.001	0.010	0.92
	rs1858037	T	A	0.38	0.030	0.038	$2.0x10^{-09}$	0.021	0.009	0.02
	rs1980422	C	T	0.24	0.148	0.046	$1.9x10^{-13}$	0.001	0.010	0.93
	rs2451258	T	C	0.43	0.068	0.041	$1.6x10^{-10}$	-0.004	0.009	0.65
	rs2476601	A	G	0.14	0.542	0.053	$8.9x10^{-170}$	-0.018	0.013	0.18
	rs2561477	G	A	0.38	0.104	0.040	$2.2x10^{-11}$	0.016	0.009	0.08
	rs3087243	G	A	0.46	0.122	0.039	$3.6x10^{-22}$	0.001	0.008	0.88
	rs3806624	G	A	0.50	0.058	0.037	$2.8x10^{-8}$	0.008	0.008	0.33
	rs4239702	C	T	0.26	0.148	0.042	$1.1x10^{-16}$	0.001	0.009	0.94

	rs4272	G	A	0.25	0.113	0.048	$1.2x10^{-8}$	-0.004	0.010	0.72
	rs4409785	C	T	0.12	0.223	0.047	$3.6x10^{-9}$	-0.004	0.011	0.73
	rs4452313	T	A	0.32	0.077	0.041	$5.2x10^{-11}$	-0.002	0.009	0.79
	rs624988	T	C	0.49	0.104	0.040	$8.0x10^{-10}$	0.021	0.008	0.01
	rs678347	G	A	0.27	0.104	0.040	$7.3x10^{-9}$	0.001	0.009	0.91
	rs706778	T	C	0.45	0.148	0.038	$4.6x10^{-15}$	0.007	0.008	0.39
	rs7752903	G	T	0.03	0.399	0.108	$1.7x10^{-20}$	0.055	0.024	0.02
	rs8026898	A	G	0.25	0.086	0.040	$5.9x10^{-18}$	0.013	0.009	0.15
	rs8032939	C	T	0.35	0.122	0.043	$3.2x10^{-14}$	-0.001	0.010	0.95
	rs909685	A	T	0.36	0.113	0.039	$6.4x10^{-12}$	0.016	0.009	0.09
	rs9372120	G	T	0.24	0.182	0.045	$3.8x10^{-8}$	-0.013	0.010	0.20
	rs947474	A	G	0.16	0.095	0.049	$3.3x10^{-10}$	0.003	0.011	0.76
	rs9653442	C	T	0.43	0.131	0.039	$9.8x10^{-15}$	0.012	0.008	0.14
	rs968567	C	T	0.18	0.148	0.050	$1.8x10^{-8}$	0.018	0.011	0.09
	rs998731	T	C	0.46	0.095	0.040	$6.6x10^{-9}$	0.002	0.008	0.86
Coronary Heart Disease										
	rs10947789	T	C	0.76	0.060	0.010	$1.6x10^{-8}$	-0.017	0.010	0.08
	rs1122608	G	T	0.76	0.092	0.034	$6.3x10^{-14}$	-0.017	0.009	0.08
	rs11556924	C	T	0.64	0.083	0.010	$6.8x10^{-17}$	-0.009	0.009	0.31
	rs12190287	C	G	0.61	0.072	0.030	$4.9x10^{-13}$	0.012	0.009	0.16
	rs12936587	G	A	0.58	0.055	0.009	$1.2x10^{-9}$	0.009	0.008	0.26

rs1561198	T	C	0.45	0.052	0.009	$4.5x10^{-9}$	-0.007	0.008	0.37
rs17114036	A	G	0.91	0.106	0.016	$5.8x10^{-12}$	-0.014	0.014	0.33
rs17514846	A	C	0.44	0.058	0.009	$4.5x10^{-10}$	0.005	0.008	0.51
rs2047009	G	T	0.50	0.053	0.009	$1.6x10^{-9}$	0.003	0.008	0.73
rs2048327	C	T	0.37	0.060	0.009	$6.9x10^{-11}$	-0.003	0.008	0.73
rs2075650	G	A	0.14	0.104	0.016	$8.6x10^{-12}$	0.004	0.012	0.73
rs2252641	C	T	0.45	0.048	0.009	$3.7x10^{-8}$	0.004	0.008	0.61
rs2281727	G	A	0.36	0.050	0.009	$7.8x10^{-9}$	-0.005	0.009	0.57
rs2505083	C	T	0.42	0.061	0.009	$1.4x10^{-11}$	0.000	0.008	0.96
rs264	G	A	0.85	0.071	0.012	$5.1x10^{-9}$	-0.012	0.012	0.30
rs273909	G	A	0.13	0.077	0.013	$1.4x10^{-8}$	-0.030	0.013	0.02
rs2895811	C	T	0.43	0.056	0.009	$4.1x10^{-10}$	-0.008	0.008	0.36
rs2954029	A	T	0.54	0.048	0.009	$4.5x10^{-8}$	-0.011	0.008	0.20
rs3184504	T	C	0.42	0.068	0.010	$5.4x10^{-11}$	0.020	0.008	0.01
rs3217992	T	C	0.38	0.145	0.009	$7.8x10^{-57}$	-0.008	0.009	0.32
rs4252120	T	C	0.72	0.062	0.010	$5.0x10^{-9}$	0.006	0.009	0.51
rs445925	C	T	0.90	0.122	0.021	$8.8x10^{-9}$	-0.001	0.014	0.92
rs4773144	G	A	0.43	0.068	0.010	$1.4x10^{-11}$	0.008	0.009	0.37
rs4845625	T	C	0.44	0.049	0.009	$3.6x10^{-8}$	0.011	0.008	0.19
rs501120	T	C	0.84	0.067	0.012	$1.8x10^{-8}$	0.016	0.012	0.18
rs515135	C	T	0.83	0.075	0.012	$4.8x10^{-10}$	0.021	0.011	0.05

	rs579459	C	T	0.21	0.071	0.013	$2.7x10^{-8}$	0.011	0.010	0.26
	rs602633	G	T	0.78	0.116	0.011	$1.5x10^{-25}$	0.016	0.010	0.11
	rs6544713	T	C	0.29	0.061	0.010	$8.7x10^{-10}$	0.006	0.009	0.50
	rs6725887	C	T	0.13	0.111	0.014	$1.2x10^{-15}$	-0.009	0.012	0.46
	rs7173743	T	C	0.57	0.065	0.009	$6.8x10^{-13}$	0.007	0.008	0.38
	rs7692387	G	A	0.80	0.065	0.011	$4.6x10^{-9}$	-0.014	0.010	0.19
	rs9319428	A	G	0.32	0.055	0.009	$1.0x10^{-8}$	-0.011	0.009	0.20
	rs9369640	A	C	0.65	0.088	0.009	$7.5x10^{-22}$	0.003	0.008	0.74
	rs9515203	T	C	0.73	0.079	0.011	$5.9x10^{-12}$	-0.002	0.010	0.88
	rs974819	T	C	0.29	0.065	0.010	$3.6x10^{-11}$	-0.002	0.009	0.87
	rs9818870	T	C	0.15	0.070	0.012	$2.6x10^{-9}$	0.013	0.011	0.24
	rs9982601	T	C	0.14	0.119	0.014	$7.7x10^{-17}$	0.007	0.012	0.58
Age at Menarche										
	rs10144321	A	G	0.75	0.040	0.006	$9.0x10^{-15}$	0.007	0.010	0.48
	rs1038903	T	C	0.73	0.040	0.006	$2.0x10^{-11}$	-0.007	0.009	0.47
	rs10423674	A	C	0.34	0.040	0.005	$9.2x10^{-12}$	-0.019	0.009	0.03
	rs10453225	G	T	0.68	0.090	0.005	$5.8x10^{-66}$	0.003	0.009	0.73
	rs10789181	A	G	0.39	0.030	0.005	$3.5x10^{-8}$	0.004	0.008	0.66
	rs1079866	G	C	0.15	0.070	0.007	$9.3x10^{-24}$	-0.002	0.012	0.85
	rs10895140	G	A	0.66	0.040	0.005	$6.7x10^{-14}$	-0.001	0.009	0.88

rs10938397	A	G	0.57	0.040	0.005	$4.0x10^{-13}$	0.014	0.008	0.11
rs10980921	C	T	0.09	0.090	0.009	$1.7x10^{-23}$	-0.003	0.015	0.86
rs11022756	A	C	0.29	0.050	0.006	$7.4x10^{-20}$	-0.015	0.009	0.09
rs11165924	A	G	0.69	0.030	0.006	$2.2x10^{-9}$	0.006	0.009	0.51
rs11215400	C	A	0.27	0.040	0.006	$6.8x10^{-11}$	-0.010	0.009	0.26
rs1129700	T	C	0.44	0.030	0.005	$2.3x10^{-9}$	-0.009	0.008	0.31
rs11578152	G	A	0.44	0.030	0.005	$4.5x10^{-8}$	-0.002	0.008	0.80
rs11715566	T	C	0.50	0.050	0.005	$2.4x10^{-27}$	0.016	0.008	0.04
rs11767400	A	C	0.30	0.040	0.006	$4.1x10^{-11}$	0.013	0.009	0.14
rs11792861	A	C	0.70	0.040	0.005	$1.7x10^{-11}$	0.014	0.009	0.13
rs12148769	G	A	0.90	0.050	0.008	$5.2x10^{-11}$	0.011	0.014	0.43
rs12446632	A	G	0.13	0.040	0.007	$1.3x10^{-8}$	0.010	0.012	0.41
rs12472911	C	T	0.20	0.040	0.006	$6.7x10^{-10}$	-0.005	0.010	0.60
rs1254337	T	A	0.31	0.040	0.005	$2.1x10^{-16}$	0.001	0.009	0.87
rs12571664	T	C	0.79	0.040	0.006	$3.3x10^{-10}$	-0.007	0.010	0.50
rs12607903	C	T	0.30	0.040	0.005	$5.4x10^{-11}$	-0.005	0.009	0.60
rs12915845	C	T	0.58	0.030	0.005	$2.7x10^{-12}$	0.008	0.008	0.36
rs13053505	G	T	0.80	0.040	0.007	$3.0x10^{-8}$	-0.010	0.011	0.36
rs13067731	T	C	0.16	0.040	0.007	$1.0x10^{-9}$	0.031	0.011	0.004
rs13135934	C	G	0.40	0.030	0.005	$1.1x10^{-10}$	-0.004	0.009	0.64
rs13179411	T	G	0.17	0.060	0.007	$3.4x10^{-20}$	-0.022	0.011	0.05

rs1324913	G	T	0.65	0.030	0.005	$3.1x10^{-10}$	0.010	0.009	0.24
rs1364063	C	T	0.43	0.050	0.005	$6.2x10^{-21}$	-0.009	0.008	0.30
rs1400974	A	G	0.64	0.050	0.005	$8.3x10^{-20}$	0.009	0.009	0.29
rs1461503	C	A	0.57	0.050	0.005	$2.7x10^{-26}$	-0.002	0.008	0.82
rs1469039	A	G	0.19	0.050	0.007	$3.5x10^{-12}$	0.004	0.011	0.71
rs1532331	G	T	0.32	0.030	0.005	$3.5x10^{-9}$	0.009	0.009	0.31
rs16860328	G	A	0.42	0.040	0.005	$1.4x10^{-16}$	0.009	0.008	0.28
rs16896742	G	A	0.38	0.040	0.006	$3.2x10^{-10}$	0.003	0.009	0.72
rs16918254	A	G	0.92	0.050	0.009	$1.4x10^{-8}$	0.011	0.016	0.49
rs16918636	T	C	0.79	0.030	0.006	$3.2x10^{-8}$	-0.013	0.010	0.19
rs17086188	A	G	0.94	0.070	0.013	$3.6x10^{-8}$	-0.009	0.024	0.72
rs17171818	C	T	0.77	0.040	0.006	$8.9x10^{-14}$	-0.005	0.010	0.63
rs1874984	C	G	0.47	0.040	0.005	$1.9x10^{-12}$	-0.003	0.008	0.72
rs1915146	G	A	0.40	0.030	0.005	$3.7x10^{-8}$	0.006	0.008	0.48
rs1958560	A	G	0.59	0.030	0.005	$3.7x10^{-8}$	0.013	0.008	0.13
rs2063730	C	A	0.18	0.050	0.007	$2.3x10^{-12}$	0.019	0.011	0.07
rs2137289	A	G	0.59	0.050	0.005	$8.2x10-^{20}$	0.001	0.009	0.90
rs2274465	C	G	0.66	0.030	0.005	$1.7x10^{-9}$	0.004	0.009	0.67
rs244293	G	A	0.60	0.030	0.005	$4.2x10^{-11}$	0.010	0.008	0.24
rs246185	C	T	0.33	0.040	0.006	$6.8x10^{-16}$	-0.007	0.009	0.42
rs2479724	T	C	0.45	0.030	0.005	$1.2x10^{-12}$	-0.005	0.008	0.54

rs251130	G	A	0.73	0.040	0.006	$2.8x10^{-10}$	0.020	0.009	0.03
rs2600959	A	G	0.34	0.040	0.005	$4.1x10-^{11}$	-0.002	0.009	0.85
rs268067	A	G	0.80	0.040	0.006	$3.3x10^{-8}$	0.003	0.011	0.75
rs2687729	G	A	0.27	0.040	0.006	$1.0x10^{-10}$	0.011	0.009	0.22
rs2836950	C	G	0.64	0.030	0.005	$6.2x10^{-11}$	0.023	0.009	0.01
rs2947411	A	G	0.17	0.060	0.007	$1.8x10^{-19}$	0.004	0.011	0.73
rs3101336	T	C	0.40	0.040	0.005	$5.2x10^{-13}$	-0.010	0.008	0.21
rs3733631	C	G	0.15	0.050	0.007	$4.8x10^{-13}$	0.013	0.011	0.25
rs3743266	T	C	0.68	0.040	0.005	$2.4x10^{-13}$	-0.003	0.009	0.75
rs4369815	T	G	0.93	0.060	0.010	$1.5x10^{-10}$	-0.007	0.016	0.68
rs466639	C	T	0.87	0.080	0.007	$2.4x10^{-24}$	0.015	0.012	0.23
rs4756059	T	C	0.92	0.070	0.010	$4.5x10^{-13}$	0.012	0.016	0.43
rs4875053	G	C	0.44	0.030	0.006	$1.3x10^{-8}$	-0.004	0.009	0.64
rs4895808	C	T	0.54	0.030	0.005	$4.8x10^{-13}$	-0.016	0.008	0.05
rs4929947	G	C	0.36	0.040	0.005	$2.6x10^{-12}$	0.018	0.009	0.03
rs543874	A	G	0.80	0.050	0.006	$1.4x10-^{15}$	0.010	0.010	0.32
rs6009583	C	T	0.74	0.030	0.006	$4.6x10^{-8}$	0.021	0.009	0.03
rs6427782	A	G	0.51	0.030	0.005	$4.6x10^{-8}$	-0.008	0.008	0.33
rs652260	T	C	0.54	0.030	0.005	$9.9x10^{-09}$	0.005	0.008	0.53
rs6555855	G	A	0.23	0.040	0.006	$2.4x10^{-09}$	-0.013	0.010	0.21

rs6563739	G	T	0.34	0.030	0.005	$2.3x10^{-11}$	0.009	0.009	0.32
rs6747380	A	G	0.17	0.070	0.007	$5.6x10^{-28}$	-0.013	0.011	0.24
rs6758290	T	C	0.50	0.040	0.005	$6.6x10^{-13}$	0.002	0.008	0.81
rs6770162	A	G	0.51	0.040	0.005	$1.5x10^{-12}$	0.009	0.008	0.25
rs6933660	C	A	0.69	0.030	0.005	$1.3x10^{-9}$	-0.004	0.009	0.66
rs6938574	T	C	0.16	0.040	0.007	$2.4x10^{-9}$	-0.002	0.012	0.86
rs6964833	T	C	0.75	0.040	0.006	$5.3x10^{-12}$	0.001	0.009	0.95
rs7103411	C	T	0.21	0.040	0.006	$2.6x10^{-11}$	0.003	0.010	0.79
rs7104764	G	A	0.25	0.030	0.006	$3.7x10^{-8}$	0.012	0.009	0.19
rs7138803	G	A	0.62	0.040	0.005	$1.7x10^{-12}$	0.018	0.008	0.03
rs7215990	G	A	0.76	0.040	0.006	$1.9x10^{-8}$	-0.016	0.010	0.10
rs7514705	C	T	0.56	0.040	0.005	$1.8x10^{-16}$	-0.001	0.008	0.90
rs7642134	G	A	0.61	0.040	0.005	$3.0x10^{-16}$	0.003	0.008	0.68
rs7647973	A	G	0.26	0.050	0.006	$1.3x10^{-16}$	0.004	0.009	0.68
rs7701886	A	G	0.58	0.030	0.005	$4.5x10^{-8}$	0.011	0.008	0.17
rs7759938	C	T	0.32	0.120	0.005	$7.8x10^{-110}$	-0.011	0.009	0.23
rs7821178	C	A	0.65	0.040	0.005	$7.3x10^{-17}$	0.006	0.009	0.53
rs7828501	G	A	0.45	0.040	0.005	$1.2x10^{-13}$	0.001	0.008	0.91
rs7853970	T	C	0.47	0.030	0.005	$2.3x10^{-9}$	0.003	0.008	0.75
rs7865468	A	G	0.70	0.030	0.005	$1.3x10^{-7}$	0.011	0.009	0.21
rs7955374	T	C	0.13	0.040	0.008	$9.5x10^{-9}$	-0.013	0.013	0.30

	rs8032675	T	C	0.40	0.040	0.005	$2.1x10^{-13}$	-0.013	0.008	0.11
	rs8050136	C	A	0.60	0.040	0.005	$1.7x10^{-17}$	0.027	0.008	0.00
	rs852069	G	A	0.64	0.040	0.005	$1.2x10^{-13}$	-0.006	0.009	0.48
	rs889122	G	T	0.72	0.040	0.006	$1.6x10^{-13}$	-0.009	0.009	0.35
	rs900400	T	C	0.61	0.030	0.005	$2.3x10^{-11}$	-0.016	0.009	0.08
	rs913588	G	A	0.49	0.030	0.005	$5.8x10^{-11}$	-0.002	0.008	0.82
	rs9321659	A	G	0.13	0.060	0.008	$2.5x10^{-16}$	0.038	0.012	0.002
	rs939317	G	A	0.74	0.040	0.006	$3.0x10^{-12}$	-0.005	0.009	0.59
	rs9447700	C	T	0.69	0.030	0.005	$5.6x10^{-9}$	0.000	0.009	1.00
	rs9475752	C	T	0.81	0.040	0.006	$8.3x10^{-12}$	0.001	0.011	0.92
	rs951366	T	C	0.60	0.030	0.005	$1.7x10^{-8}$	-0.002	0.008	0.80
	rs9560113	G	A	0.28	0.050	0.006	$2.1x10^{-17}$	0.016	0.009	0.07
	rs9635759	A	G	0.32	0.050	0.005	$1.7x10^{-24}$	0.003	0.009	0.71
	rs9647570	G	T	0.14	0.050	0.007	$1.4x10^{-11}$	0.011	0.012	0.36
	rs9849248	C	T	0.15	0.040	0.007	$1.9x10^{-8}$	-0.015	0.011	0.19
	rs988913	C	T	0.66	0.040	0.005	$1.4x10^{-12}$	0.007	0.009	0.43
Age at Menopause										
	rs1046089	A	G	0.65	-0.223	0.024	$3.7x10^{-21}$	0.010	0.016	0.51
	rs1054875	T	A	0.40	-0.188	0.021	$1.7x10^{-19}$	-0.020	0.015	0.20
	rs10734411	A	G	0.53	-0.122	0.021	$2.6x10^{-9}$	0.017	0.015	0.29

rs10852344	T	C	0.41	-0.165	0.021	$1.3x10^{-15}$	0.019	0.015	0.22
rs10905065	A	G	0.39	-0.113	0.021	$3.9x10^{-8}$	-0.007	0.015	0.67
rs10957156	A	G	0.24	-0.139	0.024	$4.5x10^{-9}$	0.005	0.018	0.79
rs11031006	G	A	0.85	-0.217	0.029	$8.5x10^{-14}$	0.034	0.022	0.12
rs11668344	G	A	0.36	-0.412	0.021	$5.5x10^{-85}$	0.021	0.016	0.19
rs11738223	A	G	0.32	-0.123	0.022	$2.0x10^{-8}$	0.001	0.017	0.94
rs12142240	T	C	0.32	-0.127	0.022	$6.6x10^{-9}$	0.009	0.016	0.58
rs12196873	A	C	0.15	-0.162	0.029	$2.8x10^{-08}$	-0.010	0.022	0.65
rs12214825	T	C	0.71	-0.167	0.023	$3.2x10^{-13}$	0.012	0.017	0.50
rs12371165	C	T	0.86	-0.182	0.030	$7.0x10^{-10}$	0.011	0.022	0.62
rs12461110	A	G	0.65	-0.174	0.022	$7.6x10^{-16}$	0.003	0.016	0.83
rs12465115	T	G	0.62	-0.194	0.028	$4.7x10^{-12}$	0.010	0.015	0.53
rs12599106	A	T	0.49	-0.116	0.021	$3.1x10^{-8}$	0.005	0.016	0.78
rs12824058	G	A	0.43	-0.136	0.021	$6.1x10^{-11}$	0.011	0.015	0.47
rs13040088	G	A	0.21	-0.158	0.025	$2.5x10^{-10}$	0.001	0.018	0.98
rs1411478	A	G	0.59	-0.132	0.021	$1.4x10^{-10}$	0.042	0.015	0.01
rs16858210	G	A	0.75	-0.141	0.024	$3.1x10^{-9}$	0.008	0.018	0.66
rs16991615	G	A	0.93	-0.875	0.044	$1.6x10^{-89}$	0.000	0.032	0.99
rs1713460	G	A	0.30	-0.144	0.023	$2.4x10^{-10}$	0.003	0.017	0.88
rs1727326	C	G	0.85	-0.195	0.032	$1.7x10^{-9}$	0.007	0.024	0.76
rs1799949	G	A	0.68	-0.139	0.021	$8.4x10^{-11}$	0.001	0.016	0.97

rs1800932	A	G	0.19	-0.173	0.026	$3.2x10^{-11}$	0.018	0.019	0.35
rs2236553	C	T	0.24	-0.157	0.025	$6.1x10^{-10}$	0.005	0.019	0.78
rs2236918	C	G	0.55	-0.153	0.021	$8.3x10^{-14}$	0.013	0.015	0.42
rs2241584	A	G	0.62	-0.139	0.021	$1.5x10^{-11}$	-0.002	0.015	0.90
rs2277339	G	T	0.10	-0.312	0.035	$1.8x10^{-19}$	0.001	0.025	0.97
rs236189	G	A	0.16	-0.177	0.028	$4.4x10^{-10}$	-0.022	0.021	0.29
rs2547274	G	C	0.91	-0.277	0.038	$3.4x10^{-13}$	0.007	0.029	0.82
rs2720044	A	C	0.16	-0.290	0.030	$7.3x10^{-22}$	0.016	0.024	0.51
rs2941505	A	G	0.68	-0.130	0.022	$1.9x10^{-09}$	0.020	0.016	0.22
rs349306	G	A	0.13	-0.227	0.036	$1.7x10^{-10}$	0.020	0.030	0.52
rs365132	G	T	0.51	-0.242	0.020	$1.4x10^{-33}$	0.022	0.015	0.16
rs4246511	C	T	0.71	-0.218	0.023	$5.1x10^{-21}$	-0.017	0.018	0.33
rs427394	G	A	0.41	-0.126	0.022	$3.8x10^{-9}$	0.018	0.015	0.24
rs4693089	A	G	0.49	-0.202	0.021	$9.2x10^{-23}$	0.004	0.016	0.80
rs4879656	A	C	0.63	-0.119	0.021	$2.0x10^{-8}$	0.005	0.016	0.78
rs4886238	G	A	0.66	-0.177	0.022	$2.5x10^{-16}$	-0.010	0.016	0.55
rs551087	G	A	0.29	-0.125	0.023	$3.9x10^{-8}$	0.032	0.017	0.07
rs5762534	T	C	0.16	-0.164	0.028	$6.1x10^{-9}$	0.012	0.021	0.57
rs6856693	A	G	0.42	-0.163	0.021	$9.8x10^{-15}$	-0.006	0.015	0.71
rs6899676	A	G	0.20	-0.229	0.025	$2.2x10^{-19}$	-0.011	0.019	0.58
rs704795	A	G	0.60	-0.163	0.021	$2.1x10^{-15}$	0.000	0.015	0.97

	rs7259376	A	G	0.54	-0.111	0.020	$4.2x10^{-8}$	0.027	0.015	0.08
	rs7573003	A	T	0.70	-0.122	0.022	$3.9x10^{-8}$	0.017	0.017	0.30
	rs763121	G	A	0.36	-0.165	0.022	$2.3x10^{-13}$	0.023	0.016	0.16
	rs7963072	G	A	0.82	-0.162	0.027	$2.1x10^{-9}$	-0.021	0.019	0.29
	rs8070740	A	G	0.24	-0.147	0.024	$1.5x10^{-9}$	0.015	0.018	0.42
	rs9039	C	T	0.28	-0.125	0.023	$3.3x10^{-8}$	0.007	0.017	0.66
	rs930036	A	G	0.63	-0.185	0.021	$3.1x10^{-19}$	0.008	0.015	0.59
	rs9379881	T	C	0.50	-0.136	0.020	$2.3x10^{-11}$	0.004	0.015	0.81
	rs9796	T	A	0.46	-0.131	0.020	$1.3x10^{-10}$	0.009	0.015	0.54
Vitamin D										
	rs10741657	G	A	0.63	-0.052	0.012	$3.3x10^{-20}$	-0.013	0.008	0.12
	rs12785878	G	T	0.73	-0.056	0.013	$2.1x10^{-27}$	-0.011	0.010	0.27
	rs2282679	G	T	0.71	-0.047	0.013	$1.9x10^{-109}$	-0.004	0.009	0.65
	rs6013897	A	T	0.80	-0.027	0.015	$6.0x10^{-10}$	0.001	0.010	0.90
Type 2 Diabetes										
	rs10203174	C	T	0.91	0.131	0.019	$9.5x10^{-12}$	0.016	0.013	0.23
	rs10401969	C	T	0.09	0.122	0.021	$7.0x10^{-09}$	0.034	0.016	0.04
	rs10811661	T	C	0.74	0.166	0.015	$3.7x10^{-27}$	0.007	0.011	0.55
	rs10830963	G	C	0.22	0.095	0.013	$5.3x10^{-13}$	0.014	0.010	0.15
	rs10842994	C	T	0.83	0.095	0.015	$6.1x10^{-10}$	0.001	0.010	0.90
	rs1111875	C	T	0.59	0.104	0.012	$2.0x10^{-19}$	0.001	0.008	0.89

rs11651052	A	G	0.48	0.095	0.014	$2.0x10^{-11}$	-0.007	0.011	0.50
rs11717195	T	C	0.80	0.104	0.014	$6.5x10^{-14}$	-0.027	0.010	0.01
rs12571751	A	G	0.56	0.077	0.012	$1.0x10^{-10}$	-0.001	0.008	0.90
rs12899811	G	A	0.25	0.077	0.013	$6.3x10^{-9}$	0.016	0.009	0.06
rs12970134	A	G	0.28	0.077	0.014	$1.2x10^{-8}$	0.010	0.009	0.26
rs13389219	C	T	0.60	0.068	0.012	$1.0x10^{-8}$	0.000	0.008	0.97
rs1359790	G	A	0.73	0.077	0.014	$1.4x10^{-8}$	0.014	0.009	0.13
rs1496653	A	G	0.91	0.086	0.015	$3.6x10^{-9}$	-0.019	0.010	0.05
rs1552224	A	C	0.87	0.104	0.016	$1.8x10^{-10}$	-0.002	0.011	0.85
rs163184	G	T	0.48	0.086	0.013	$1.2x10^{-11}$	-0.018	0.008	0.03
rs17168486	T	C	0.14	0.104	0.016	$5.9x10^{-11}$	0.001	0.011	0.93
rs1801282	C	G	0.91	0.122	0.017	$1.1x10^{-12}$	-0.012	0.012	0.34
rs2075423	G	T	0.71	0.068	0.012	$8.1x10^{-9}$	-0.002	0.009	0.81
rs2261181	T	C	0.09	0.122	0.020	$1.2x10^{-9}$	0.013	0.014	0.35
rs243088	T	A	0.45	0.068	0.012	$1.8x10^{-8}$	0.004	0.008	0.62
rs2796441	G	A	0.62	0.068	0.012	$5.4x10^{-9}$	-0.001	0.008	0.86
rs2943640	C	A	0.66	0.095	0.013	$2.7x10^{-14}$	0.004	0.009	0.67
rs3802177	G	A	0.72	0.131	0.014	$1.3x10^{-21}$	-0.020	0.009	0.03
rs4402960	T	G	0.28	0.122	0.012	$2.4x10^{-23}$	-0.020	0.009	0.02
rs4458523	G	T	0.67	0.095	0.012	$2.0x10^{-15}$	-0.001	0.008	0.88
rs459193	G	A	0.75	0.077	0.013	$6.0x10^{-9}$	-0.015	0.009	0.11

rs516946	C	T	0.80	0.086	0.014	2.5x10^{-10}	-0.013	0.010	0.19
rs5215	C	T	0.43	0.068	0.011	8.5x10^{-10}	0.004	0.008	0.61
rs6795735	C	T	0.52	0.077	0.012	7.4x10^{-11}	-0.009	0.008	0.27
rs6878122	G	A	0.32	0.095	0.015	5.0x10^{-11}	-0.003	0.009	0.76
rs7177055	A	G	0.71	0.077	0.013	4.6x10^{-9}	-0.023	0.009	0.01
rs7202877	T	G	0.91	0.113	0.021	3.5x10^{-8}	0.003	0.014	0.85
rs7756992	G	A	0.25	0.157	0.013	7.0x10^{-35}	0.009	0.009	0.32
rs7903146	T	C	0.31	0.329	0.013	1.2x10^{-139}	0.018	0.009	0.04
rs7955901	C	T	0.46	0.068	0.012	6.5x10^{-9}	-0.006	0.008	0.46
rs849135	G	A	0.51	0.104	0.012	3.1x10^{-17}	-0.002	0.008	0.78
rs9936385	C	T	0.43	0.122	0.012	2.6x10^{-23}	-0.027	0.008	0.001
Thyroid-Stimulating Hormone							0.0x10+00		
rs10032216	C	T	0.22	-0.087	0.011	9.3x10^{-16}	0.005	0.010	0.63
rs10519227	A	T	0.25	-0.072	0.011	1.0x10^{-11}	0.002	0.010	0.81
rs10799824	A	G	0.16	-0.113	0.012	0.0x10+00	0.019	0.011	0.09
rs11624776	A	C	0.66	-0.064	0.011	1.8x10^{-09}	-0.001	0.009	0.96
rs11755845	T	C	0.27	-0.065	0.010	1.7x10-^{10}	-0.009	0.010	0.35
rs13015993	G	A	0.26	-0.078	0.010	3.2x10^{-15}	0.011	0.009	0.22
rs1537424	T	C	0.61	-0.052	0.009	1.2x10^{-08}	0.016	0.008	0.06
rs1571583	G	A	0.75	-0.057	0.010	2.6x10^{-08}	-0.007	0.010	0.48
rs17723470	T	C	0.28	-0.065	0.010	8.8x10^{-11}	0.000	0.009	0.98

	rs17776563	A	G	0.32	-0.060	0.010	$2.9x10^{-10}$	0.005	0.009	0.61
	rs334699	A	G	0.05	-0.141	0.021	$5.4x10^{-12}$	-0.019	0.020	0.32
	rs3813582	C	T	0.33	-0.082	0.010	$8.5x10^{-18}$	-0.030	0.009	$7.1x10^{-04}$
	rs4804416	T	G	0.57	-0.057	0.009	$3.2x10^{-10}$	0.007	0.008	0.37
	rs657152	C	A	0.64	-0.058	0.009	$4.1x10^{-10}$	-0.003	0.009	0.76
	rs6885099	A	G	0.59	-0.141	0.009	0.0x10+00	-0.004	0.008	0.64
	rs753760	G	C	0.31	-0.100	0.010	0.0x10+00	0.002	0.009	0.84
	rs7825175	A	G	0.21	-0.066	0.011	$2.9x10^{-09}$	-0.005	0.010	0.60
	rs9472138	T	C	0.29	-0.079	0.010	$6.7x10^{-16}$	-0.009	0.009	0.32
	rs9497965	C	T	0.59	-0.051	0.009	$2.3x10-^{08}$	0.004	0.008	0.68
	rs9915657	T	C	0.54	-0.064	0.009	$7.5x10^{-13}$	0.001	0.008	0.87
Inflammatory Bowel Disease								0.0x10+00		
	rs10051722	A	C	0.70	0.070	0.016	$6.2x10^{-07}$	0.003	0.009	0.72
	rs10061469	T	C	0.67	0.084	0.018	$5.6x10^{-10}$	0.003	0.009	0.72
	rs10065637	C	T	0.79	0.116	0.021	$3.7x10^{-12}$	-0.001	0.010	0.96
	rs1042058	C	T	0.60	0.072	0.015	$2.1x10^{-10}$	0.011	0.008	0.20
	rs10486483	A	G	0.24	0.085	0.019	$2.6x10^{-08}$	0.013	0.009	0.16
	rs10495903	T	C	0.13	0.083	0.021	$3.2x10^{-10}$	-0.013	0.012	0.30
	rs10758669	C	A	0.34	0.160	0.015	$7.9x10^{-45}$	-0.006	0.009	0.52
	rs10761659	G	A	0.54	0.154	0.015	$8.5x10^{-45}$	0.001	0.008	0.88
	rs10781499	A	G	0.42	0.172	0.014	$4.4x10^{-56}$	0.008	0.008	0.33

rs10865331	A	G	0.38	0.093	0.016	$9.8x10^{-10}$	0.000	0.008	0.97
rs11010067	G	C	0.35	0.109	0.015	$6.7x10^{-23}$	-0.013	0.009	0.13
rs11054935	G	A	0.28	0.056	0.016	$2.6x10^{-06}$	-0.012	0.009	0.21
rs11083840	G	T	0.41	0.072	0.018	$1.2x10^{-08}$	-0.006	0.009	0.45
rs11150589	T	C	0.47	0.086	0.017	$6.0x10^{-10}$	0.002	0.008	0.77
rs11168249	C	T	0.47	0.053	0.014	$4.6x10^{-08}$	0.000	0.008	1.00
rs11209026	G	A	0.94	0.700	0.034	$8.1x10^{-161}$	-0.006	0.017	0.74
rs11229555	G	T	0.76	0.077	0.017	$6.8x10^{-10}$	0.026	0.010	0.01
rs11230563	C	T	0.66	0.082	0.015	$9.0x10^{-13}$	-0.003	0.009	0.73
rs11564258	A	G	0.02	0.288	0.046	$5.0x10^{-23}$	-0.078	0.031	0.01
rs11739663	T	C	0.76	0.069	0.021	$1.8x10^{-08}$	-0.012	0.010	0.20
rs11742570	C	T	0.61	0.181	0.015	$3.5x10^{-59}$	0.014	0.008	0.10
rs1182188	T	C	0.70	0.120	0.020	$6.1x10^{-17}$	0.003	0.009	0.73
rs11879191	G	A	0.80	0.128	0.018	$2.0x10^{-18}$	-0.007	0.010	0.49
rs12199775	A	G	0.93	0.121	0.029	$2.0x10^{-08}$	-0.020	0.016	0.21
rs1250546	A	G	0.57	0.092	0.015	$8.3x10^{-15}$	-0.024	0.008	0.004
rs12568930	T	C	0.83	0.091	0.020	$7.4x10^{-14}$	0.054	0.011	$5.5x10^{-07}$
rs1260326	T	C	0.40	0.116	0.017	$4.9x10^{-16}$	-0.002	0.008	0.82
rs12663356	C	T	0.53	0.091	0.017	$4.0x10^{-12}$	0.010	0.008	0.21
rs12722515	C	A	0.84	0.097	0.020	$5.8x10^{-09}$	0.010	0.011	0.36
rs12778642	T	G	0.44	0.064	0.015	$3.0x10^{-08}$	0.001	0.008	0.93

rs1292053	G	A	0.44	0.073	0.014	$1.8x10^{-10}$	-0.014	0.008	0.09
rs12994997	A	G	0.52	0.209	0.017	$4.1x10^{-70}$	0.000	0.008	0.99
rs13204742	T	G	0.13	0.160	0.024	$8.4x10^{-15}$	0.007	0.012	0.59
rs13277237	G	A	0.43	0.053	0.014	$1.7x10^{-09}$	-0.002	0.008	0.78
rs13407913	G	A	0.44	0.103	0.015	$9.4x10^{-15}$	0.015	0.008	0.06
rs1363907	A	G	0.43	0.066	0.015	$8.7x10^{-12}$	0.005	0.008	0.57
rs1440088	T	G	0.80	0.095	0.023	$2.9x10^{-08}$	-0.004	0.010	0.70
rs1456896	T	C	0.67	0.084	0.016	$9.8x10^{-14}$	-0.006	0.009	0.46
rs1517352	C	A	0.62	0.074	0.015	$3.3x10^{-11}$	-0.004	0.008	0.64
rs1569328	C	T	0.83	0.080	0.019	$3.4x10^{-06}$	0.011	0.011	0.32
rs1654644	G	T	0.35	0.083	0.015	$2.5x10^{-09}$	0.006	0.009	0.50
rs17085007	C	T	0.18	0.101	0.019	$3.8x10^{-13}$	-0.006	0.011	0.60
rs17119	A	G	0.80	0.069	0.018	$3.1x10^{-11}$	-0.007	0.011	0.53
rs17229285	C	T	0.49	0.111	0.018	$1.7x10^{-13}$	-0.017	0.008	0.04
rs1728785	C	A	0.77	0.072	0.021	$3.7x10^{-08}$	-0.001	0.010	0.92
rs17293632	T	C	0.24	0.065	0.016	$5.9x10^{-15}$	-0.010	0.010	0.28
rs1734907	A	G	0.15	0.108	0.020	$1.9x10^{-10}$	-0.023	0.012	0.05
rs17391694	C	T	0.87	0.126	0.026	$3.0x10^{-09}$	-0.033	0.013	0.01
rs174537	T	G	0.35	0.076	0.015	$1.5x10^{-10}$	-0.014	0.009	0.09
rs17694108	A	G	0.29	0.095	0.016	$5.9x10^{-15}$	-0.001	0.010	0.90
rs17695092	T	G	0.69	0.091	0.019	$4.7x10^{-09}$	-0.006	0.009	0.51

rs17780256	A	C	0.81	0.105	0.023	$1.9x10^{-09}$	0.004	0.011	0.72
rs1801274	A	G	0.48	0.117	0.015	$3.5x10^{-28}$	-0.006	0.008	0.45
rs1819333	T	G	0.53	0.078	0.015	$3.6x10^{-15}$	-0.001	0.008	0.90
rs1847472	C	A	0.65	0.058	0.015	$1.1x10^{-09}$	0.011	0.009	0.20
rs1893217	G	A	0.17	0.158	0.019	$3.1x10^{-26}$	0.013	0.011	0.22
rs194749	C	T	0.24	0.072	0.017	$3.9x10^{-08}$	-0.017	0.010	0.07
rs2024092	A	G	0.21	0.145	0.019	$8.3x10^{-22}$	0.017	0.010	0.09
rs2111485	A	G	0.39	0.064	0.015	$2.5x10^{-07}$	-0.005	0.008	0.52
rs212388	C	T	0.41	0.100	0.016	$3.0x10^{-14}$	0.008	0.008	0.35
rs2155219	T	G	0.49	0.141	0.015	$4.2x10^{-36}$	0.004	0.008	0.60
rs2188962	T	C	0.42	0.147	0.014	$3.7x10^{-37}$	0.006	0.008	0.43
rs2226628	A	G	0.29	0.080	0.016	$2.3x10^{-08}$	0.002	0.009	0.86
rs2227551	T	G	0.72	0.079	0.017	$6.7x10^{-06}$	0.004	0.009	0.70
rs2266959	T	G	0.19	0.100	0.018	$1.4x10^{-16}$	0.027	0.010	0.01
rs2284553	G	A	0.59	0.116	0.017	$2.1x10^{-16}$	-0.007	0.008	0.42
rs2361755	G	C	0.92	0.144	0.028	$1.4x10^{-9}$	0.000	0.015	0.98
rs2382817	A	C	0.41	0.070	0.015	$3.7x10^{-12}$	-0.019	0.008	0.02
rs2413583	C	T	0.84	0.190	0.020	$4.4x10^{-33}$	-0.001	0.011	0.92
rs2457996	T	C	0.88	0.091	0.023	$4.2x10^{-7}$	-0.003	0.013	0.82

rs2488389	A	G	0.22	0.109	0.017	$5.2x10^{-12}$	-0.007	0.010	0.47
rs2503322	G	A	0.55	0.074	0.016	$3.8x10^{-10}$	-0.056	0.008	$7.7x10^{-12}$
rs254560	A	G	0.41	0.054	0.018	$2.6x10^{-9}$	0.006	0.008	0.44
rs259964	A	G	0.45	0.082	0.014	$1.0x10^{-12}$	-0.002	0.008	0.83
rs2641348	A	G	0.89	0.149	0.027	$2.0x10^{-11}$	0.009	0.013	0.51
rs2651244	G	A	0.61	0.015	0.014	0.48	0.010	0.008	0.26
rs26528	C	T	0.46	0.094	0.014	$4.7x10^{-18}$	0.017	0.010	0.10
rs2790216	G	A	0.79	0.064	0.018	$1.6x10^{-7}$	0.001	0.010	0.95
rs2816958	G	A	0.88	0.207	0.029	$2.0x10^{-17}$	0.005	0.013	0.73
rs2823286	G	A	0.71	0.146	0.016	$9.3x10^{-30}$	0.001	0.009	0.94
rs2836878	G	A	0.73	0.166	0.017	$7.3x10^{-43}$	0.021	0.009	0.02
rs2930047	C	T	0.37	0.063	0.015	$1.0x10^{-8}$	0.009	0.009	0.31
rs2945412	A	G	0.59	0.128	0.017	$8.7x10^{-17}$	-0.002	0.008	0.86
rs3024505	A	G	0.15	0.189	0.019	$6.7x10^{-42}$	-0.007	0.011	0.55
rs3091315	A	G	0.72	0.115	0.016	$5.3x10^{-20}$	-0.014	0.009	0.12
rs3197999	A	G	0.29	0.166	0.015	$1.0x10^{-47}$	0.012	0.009	0.19
rs3742130	G	A	0.79	0.106	0.018	$2.4x10^{-14}$	0.001	0.010	0.94
rs3764147	G	A	0.22	0.144	0.019	$2.2x10^{-21}$	-0.011	0.010	0.28
rs3766606	G	T	0.82	0.106	0.020	$1.1x10^{-15}$	-0.020	0.011	0.07
rs3774937	A	G	0.34	0.112	0.018	$3.7x10-^{12}$	0.010	0.009	0.26
rs3851228	T	A	0.06	0.142	0.028	$1.1x10^{-13}$	0.033	0.017	0.04

rs38911	G	A	0.53	0.053	0.015	$2.6x10^{-7}$	-0.006	0.008	0.50
rs4243971	G	T	0.56	0.070	0.015	$6.1x10^{-10}$	0.011	0.008	0.18
rs4246905	C	T	0.72	0.133	0.016	$2.8x10^{-32}$	0.007	0.009	0.45
rs4256159	T	C	0.15	0.102	0.020	$1.7x10^{-11}$	-0.006	0.011	0.58
rs4380874	T	C	0.41	0.128	0.018	$2.1x10^{-26}$	0.007	0.008	0.42
rs4409764	T	G	0.48	0.167	0.015	$1.0x10^{-54}$	0.006	0.008	0.46
rs4722672	C	T	0.18	0.087	0.022	$2.1x10^{-8}$	-0.011	0.011	0.30
rs4728142	A	G	0.45	0.099	0.018	$4.4x10^{-14}$	0.015	0.008	0.06
rs4743820	T	C	0.71	0.054	0.016	$6.5x10^{-9}$	0.009	0.009	0.32
rs477515	G	A	0.65	0.367	0.020	$4.7x10^{-133}$	-0.015	0.011	0.16
rs4802307	G	T	0.70	0.094	0.018	$2.0x10^{-10}$	-0.010	0.009	0.27
rs483905	A	G	0.29	0.054	0.019	$1.2x10^{-8}$	-0.002	0.009	0.87
rs4845604	G	A	0.85	0.135	0.021	$5.3x10^{-16}$	0.013	0.012	0.26
rs4976646	A	G	0.34	0.066	0.015	$1.7x10^{-8}$	-0.010	0.009	0.25
rs516246	T	C	0.50	0.102	0.016	$1.0x10^{-15}$	0.018	0.008	0.03
rs529866	C	T	0.80	0.117	0.019	$1.5x10^{-12}$	0.011	0.010	0.26
rs559928	C	T	0.81	0.096	0.019	$4.2x10^{-11}$	0.022	0.010	0.04
rs561722	C	T	0.67	0.113	0.019	$5.2x10^{-17}$	0.015	0.009	0.08
rs566416	T	G	0.77	0.071	0.017	$2.6x10^{-6}$	0.005	0.010	0.61
rs568617	T	C	0.18	0.080	0.018	$3.0x10^{-6}$	-0.009	0.011	0.42
rs5743289	T	C	0.17	0.443	0.020	$5.6x10^{-166}$	-0.016	0.011	0.16

rs5763767	A	G	0.45	0.077	0.014	$2.7x10^{-14}$	-0.004	0.008	0.65
rs6017342	C	A	0.53	0.205	0.018	$1.4x10^{-43}$	-0.004	0.009	0.66
rs6074022	C	T	0.25	0.087	0.016	$6.6x10^{-11}$	-0.006	0.009	0.52
rs6087990	C	T	0.41	0.072	0.015	$1.2x10^{-9}$	0.018	0.008	0.03
rs6088765	G	T	0.43	0.076	0.018	$2.2x10^{-8}$	-0.007	0.008	0.38
rs6426833	A	G	0.54	0.235	0.018	$2.4x10^{-68}$	-0.002	0.008	0.85
rs6651252	T	C	0.87	0.170	0.026	$1.5x10^{-16}$	-0.015	0.012	0.22
rs6667605	C	T	0.50	0.075	0.018	$2.6x10^{-12}$	0.015	0.008	0.07
rs6679677	C	A	0.90	0.179	0.030	$2.0x10^{-15}$	0.018	0.014	0.18
rs670523	A	G	0.33	0.058	0.015	$3.7x10^{-7}$	0.001	0.009	0.88
rs6708413	G	A	0.23	0.098	0.017	$6.2x10^{-17}$	0.006	0.010	0.52
rs6716753	C	T	0.19	0.126	0.020	$1.2x10^{-16}$	-0.006	0.010	0.59
rs6740462	A	C	0.73	0.078	0.016	$2.4x10^{-8}$	-0.014	0.012	0.24
rs6863411	T	A	0.62	0.085	0.015	$1.3x10^{-12}$	-0.006	0.008	0.45
rs6871626	A	C	0.34	0.166	0.015	$1.4x10^{-42}$	0.006	0.009	0.50
rs6920220	A	G	0.21	0.097	0.018	$1.0x10^{-14}$	-0.001	0.010	0.94
rs7015630	T	C	0.74	0.072	0.019	$1.4x10^{-8}$	-0.006	0.009	0.49
rs7097656	C	T	0.80	0.109	0.018	$1.3x10^{-14}$	0.001	0.010	0.95
rs7134599	A	G	0.39	0.092	0.015	$1.2x10^{-22}$	-0.005	0.008	0.56
rs7240004	A	G	0.63	0.055	0.015	$9.8x10^{-9}$	-0.006	0.008	0.51
rs727088	G	A	0.48	0.074	0.014	$4.7x10^{-9}$	0.012	0.008	0.16

	rs7282490	G	A	0.40	0.100	0.015	$2.4x10^{-26}$	0.000	0.008	0.96
	rs7438704	G	A	0.65	0.083	0.017	$1.8x10^{-8}$	0.003	0.009	0.75
	rs7495132	C	T	0.88	0.126	0.024	$9.5x10^{-11}$	-0.003	0.013	0.82
	rs7517810	T	C	0.24	0.118	0.019	$5.5x10^{-22}$	0.014	0.010	0.14
	rs7554511	C	A	0.70	0.152	0.016	$1.2x10^{-32}$	0.007	0.009	0.45
	rs7657746	A	G	0.74	0.110	0.017	$2.8x10^{-13}$	0.002	0.009	0.82
	rs7746082	C	G	0.29	0.103	0.015	$2.5x10^{-17}$	0.002	0.009	0.87
	rs8005161	T	C	0.09	0.142	0.025	$2.4x10^{-14}$	-0.008	0.014	0.59
	rs864745	T	C	0.50	0.083	0.017	$3.7x10^{-9}$	0.000	0.008	0.96
	rs907611	A	G	0.32	0.066	0.016	$2.7x10^{-10}$	0.013	0.009	0.16
	rs913678	T	C	0.67	0.054	0.015	$4.6x10^{-8}$	-0.001	0.009	0.92
	rs921720	G	A	0.63	0.078	0.015	$6.7x10^{-15}$	-0.002	0.008	0.80
	rs925255	C	T	0.52	0.088	0.015	$2.5x10^{-14}$	-0.007	0.008	0.38
	rs9264942	C	T	0.35	0.135	0.017	$5.0x10^{-28}$	-0.007	0.009	0.44
	rs9297145	C	A	0.26	0.079	0.016	$8.2x10^{-12}$	-0.004	0.009	0.69
	rs9358372	G	A	0.37	0.085	0.015	$1.6x10^{-12}$	0.000	0.008	0.96
	rs941823	C	T	0.75	0.069	0.017	$3.8x10^{-10}$	-0.005	0.009	0.63
	rs9847710	C	T	0.42	0.062	0.018	$1.1x10^{-8}$	-0.015	0.008	0.08
Type 1 Diabetes										
	rs10492166	G	A	0.50	0.139	NA	$6.0x10^{-9}$	-0.014	0.012	0.23
	rs10758593	A	G	0.40	0.140	NA	$1.2x10^{-8}$	-0.005	0.008	0.52

	rs11571316	G	A	0.58	0.198	NA	$2.4x10^{-15}$	0.004	0.008	0.62
	rs12908309	G	A	0.76	0.163	NA	$4.3x10^{-8}$	0.002	0.010	0.86
	rs12927355	C	T	0.68	0.223	NA	$1.9x10^{-16}$	-0.006	0.009	0.53
	rs1893217	G	A	0.17	0.182	NA	$1.6x10^{-8}$	0.013	0.011	0.22
	rs1990760	T	C	0.61	0.139	NA	$2.2x10^{-8}$	0.008	0.008	0.34
	rs2476601	A	G	0.10	0.673	NA	$5.9x10^{-80}$	-0.018	0.013	0.18
	rs3024493	C	A	0.85	0.198	NA	$2.0x10^{-8}$	0.006	0.011	0.62
	rs3184504	T	C	0.48	0.236	NA	$1.8x10^{-21}$	0.020	0.008	0.01
	rs478222	A	T	0.59	0.139	NA	$3.5x10^{-9}$	0.008	0.008	0.32
	rs539514	T	A	0.51	0.128	NA	$5.7x10^{-11}$	0.005	0.008	0.56
	rs597325	G	A	0.61	0.163	NA	$3.4x10^{-10}$	-0.008	0.008	0.34
	rs6916742	C	T	0.54	1.427	NA	$4.0x10^{-307}$	0.006	0.014	0.67
	rs705704	A	G	0.34	0.300	NA	$4.3x10^{-31}$	-0.008	0.009	0.37
	rs7090530	A	C	0.60	0.198	NA	$2.9x10^{-15}$	-0.003	0.008	0.75
	rs7928968	T	A	0.23	0.223	NA	$2.8x10^{-14}$	0.015	0.011	0.15
	rs924043	C	T	0.86	0.174	NA	$8.1x10^{-9}$	-0.004	0.012	0.77
	rs9924471	A	G	0.16	0.215	NA	$1.2x10^{-11}$	0.010	0.011	0.35
Fasting Glucose										
	rs10305492	G	A	0.98	0.090	0.013	$3.4x10^{-12}$	-0.012	0.037	0.75
	rs10747083	A	G	0.68	0.013	0.002	$7.6x10^{-9}$	-0.012	0.009	0.18

rs10811661	T	C	0.82	0.024	0.003	$5.7x10^{-18}$	0.007	0.011	0.55
rs10814916	C	A	0.51	0.016	0.002	$2.3x10^{-13}$	-0.006	0.008	0.49
rs10830963	G	C	0.28	0.078	0.003	$1.1x10^{-215}$	0.014	0.010	0.15
rs11039182	T	C	0.72	0.023	0.002	$4.8x10^{-22}$	-0.011	0.009	0.24
rs11195502	C	T	0.91	0.032	0.004	$2.0x10^{-18}$	0.002	0.014	0.89
rs11558471	A	G	0.68	0.029	0.002	$7.8x10^{-37}$	-0.019	0.009	0.03
rs11603334	G	A	0.84	0.019	0.003	$1.1x10^{-11}$	-0.003	0.011	0.82
rs11607883	G	A	0.48	0.021	0.002	$6.3x10^{-24}$	-0.014	0.008	0.10
rs11619319	G	A	0.22	0.020	0.002	$1.3x10^{-15}$	0.001	0.010	0.89
rs11708067	A	G	0.76	0.023	0.003	$1.3x10^{-18}$	-0.024	0.010	0.01
rs11715915	C	T	0.69	0.012	0.002	$4.9x10^{-8}$	-0.012	0.009	0.16
rs1280	T	C	0.87	0.026	0.003	$8.6x10^{-18}$	0.020	0.012	0.10
rs16913693	T	G	0.97	0.043	0.007	$3.5x10^{-11}$	0.083	0.027	0.002
rs174576	C	A	0.65	0.020	0.002	$1.2x10^{-18}$	0.016	0.009	0.07
rs2191349	T	G	0.54	0.029	0.002	$1.3x10^{-42}$	0.012	0.008	0.16
rs2302593	C	G	0.51	0.014	0.002	$9.3x10^{-10}$	0.007	0.009	0.44
rs2908289	A	G	0.17	0.057	0.003	$3.3x10^{-88}$	0.009	0.011	0.42
rs340874	C	T	0.56	0.013	0.002	$4.1x10^{-10}$	-0.004	0.008	0.62
rs3783347	G	T	0.77	0.017	0.003	$1.3x10^{-10}$	-0.020	0.010	0.05
rs3829109	G	A	0.73	0.017	0.003	$1.1x10^{-10}$	-0.001	0.010	0.95
rs4502156	T	C	0.56	0.022	0.002	$1.4x10^{-25}$	0.002	0.008	0.78

	rs4869272	T	C	0.69	0.018	0.002	$1.0x10^{-15}$	0.004	0.009	0.63
	rs560887	C	T	0.70	0.071	0.003	$1.4x10^{-178}$	0.014	0.009	0.13
	rs576674	G	A	0.17	0.017	0.003	$2.3x10^{-8}$	-0.003	0.011	0.81
	rs6072275	A	G	0.15	0.016	0.003	$1.7x10^{-8}$	0.005	0.011	0.64
	rs6113722	G	A	0.96	0.035	0.005	$2.5x10^{-11}$	0.001	0.022	0.96
	rs651007	G	A	0.79	0.020	0.000	$1.3x10^{-8}$	-0.011	0.010	0.27
	rs6943153	T	C	0.32	0.015	0.002	$1.6x10^{-12}$	-0.006	0.009	0.47
	rs7651090	G	A	0.31	0.013	0.002	$1.8x10^{-8}$	-0.020	0.009	0.03
	rs780094	C	T	0.61	0.027	0.002	$2.6x10^{-37}$	0.007	0.008	0.39
	rs7903146	T	C	0.29	0.022	0.002	$2.7x10^{-20}$	0.018	0.009	0.04
	rs9368222	A	C	0.27	0.014	0.002	$1.0x10^{-9}$	0.011	0.009	0.23
	rs983309	T	G	0.12	0.026	0.003	$6.3x10^{-15}$	-0.020	0.013	0.12
Grip Strength *										
	rs11236185	A	G	0.51	-0.164	0.026	$3.8x10^{-10}$	0.02	0.01	0.20
	rs11614333	T	C	0.37	-0.181	0.027	$5.0x10^{-11}$	-0.03	0.01	0.04
	rs1556659	C	T	0.63	-0.156	0.027	$6.8x10^{-09}$	-0.02	0.01	0.24
	rs17563986	G	A	0.21	-0.181	0.031	$5.2x10^{-09}$	-0.03	0.02	0.07
	rs2110927	T	C	0.74	-0.161	0.029	$4.4x10^{-8}$	0.01	0.01	0.44
	rs2273555	G	A	0.41	-0.153	0.027	$9.1x10^{-9}$	-0.02	0.01	0.10
	rs2288278	G	A	0.35	-0.162	0.027	$3.0x10^{-9}$	0.01	0.01	0.40

rs329676	C	G	0.66	-0.141	0.027	$1.6x10^{-7}$	0.00	0.01	0.92
rs4926611	T	C	0.37	-0.173	0.027	$1.3x10^{-10}$	0.03	0.01	0.05
rs6539344	T	G	0.58	-0.144	0.026	$4.9x10^{-8}$	-0.01	0.01	0.53
rs660895	G	A	0.20	-0.176	0.032	$3.1x10^{-8}$	-0.03	0.02	0.10
rs6687430	A	G	0.56	-0.150	0.026	$7.6x10^{-9}$	0.00	0.01	0.72
rs7558957	C	T	0.51	-0.158	0.027	$4.3x10^{-9}$	0.00	0.01	0.88
rs958685	C	A	0.50	-0.154	0.026	$2.8x10^{-9}$	0.02	0.01	0.22
rs9914011	C	G	0.71	0.125	0.029	$1.6x10^{-5}$	0.00	0.02	0.88

*the fracture effect estimates are derived after excluding EPIC and UKBB from the fracture meta-analysis

Table S6A | Independent Signals from the discovery phase with p-values < 5 x 10^{-6}

Locus	SNP	Effect Allele	Effect Allele Frequency	Odds Ratio	P-value	Closest Gene
7q31.31	rs2908007	a	0.60	1.08	1.23E-20	WNT16, FAM3C
6q22.33	rs10457487	c	0.51	1.07	2.32E-15	RSPO3
10q21.1	rs11003047	g	0.11	1.10	6.17E-13	MBL2,DKK1
18p11.21	rs4635400	a	0.36	1.06	1.52E-12	FAM210A
17q21.31	rs2741856	g	0.92	1.11	2.36E-12	MEOX1,SOST,DUSP3
17q21.31	rs4793022	g	0.62	1.06	7.30E-12	MEOX1,SOST,DUSP3
6q25.1	rs2982570	c	0.58	1.06	8.11E-12	ESR1
21q22.2	rs9980072	g	0.73	1.06	8.34E-12	ETS2
6q22.33	rs9491689	c	0.71	1.06	2.39E-10	RSPO3
7q21.3	rs6465508	g	0.34	1.05	3.98E-09	C7orf76, SHFM1
7q31.31	rs2536189	c	0.56	1.05	1.84E-08	WNT16, FAM3C
6q22.33	rs1930955	g	0.45	1.05	1.87E-08	RSPO3
4p16.3	rs138081769	c	0.05	1.15	2.66E-08	PCGF3
7p12.1	rs1548607	g	0.32	1.05	3.24E-08	GRB10,COBL
2p22.1	rs11900510	c	0.40	1.06	4.67E-08	SLC8A1
7p14.1	rs10280461	c	0.46	1.04	9.35E-08	STARD3NL
10q21.1	rs10762857	c	0.37	1.05	2.16E-07	MBL2
4p16.3	rs11937228	a	0.12	1.07	2.44E-07	SPON2
18q12.2	rs79434015	t	0.91	1.10	2.55E-07	-
5q31.1	rs10052731	c	0.16	1.06	2.70E-07	TGFBI
7q31.31	rs17284876	g	0.82	1.06	3.06E-07	CPED1
21q22.2	rs2836778	c	0.61	1.05	3.40E-07	ETS2
8p21.3	rs4921656	c	0.79	1.05	3.86E-07	SCGALNACT1
5q34	rs12517141	t	0.63	1.06	4.26E-07	GABRA6
14q24.1	rs11626090	a	0.27	1.05	4.42E-07	RAD52B

16q12.2	rs8059888	c	0.51	1.04	5.45E-07	IRX5
1p36.12	rs12568930	t	0.82	1.06	6.03E-07	ZBTB40
14q32.12	rs1075472	g	0.18	1.05	6.19E-07	RIN3
6q25.1	rs6925996	c	0.30	1.05	6.57E-07	CCDC170
5q14.1	rs378912	a	0.18	1.05	6.80E-07	ARSB
6q25.1	rs6929137	a	0.33	1.04	8.43E-07	CCDC170
13q14.3	rs4883723	a	0.14	1.06	8.90E-07	OLFM4
10q21.1	rs17662737	t	0.77	1.05	9.11E-07	MBL2
7p12.3	rs71547894	c	0.60	1.05	9.83E-07	ABCA13
10q26.13	rs7082865	a	0.46	1.04	1.16E-06	BTB16
1p36.12	rs10753536	g	0.61	1.04	1.28E-06	ZBTB40
6q22.33	rs9491675	g	0.86	1.06	1.35E-06	RSPO3
2p21	rs13430285	a	0.22	1.05	1.42E-06	SIX2
21q22.11	rs2833404	t	0.05	1.12	1.55E-06	TIAM1
2q32.2	rs17271239	g	0.02	1.22	1.73E-06	ANKAR
2p22.1	rs10490046	c	0.23	1.05	1.84E-06	SLC8A1
10q22.1	rs3902977	a	0.26	1.05	2.08E-06	PCBD1
2p24.3	rs10180470	g	0.88	1.06	2.45E-06	TRIB2
17q21.31	rs3859274	t	0.72	1.06	2.76E-06	C17orf53
17q25.1	rs17462688	a	0.86	1.06	2.88E-06	SD300C
4q13.3	rs12642402	a	0.05	1.10	2.98E-06	AREG,AREGB
18q12.2	rs4145297	c	0.59	1.05	3.42E-06	-
14q21.2	rs2415962	t	0.55	1.04	3.44E-06	RPL10L
6q12	rs9351261	t	0.63	1.04	3.77E-06	EYS
13q31.1	rs117906587	t	0.02	1.22	3.82E-06	RNAF219,RBM26
5q35.2	rs114233559	a	0.01	1.23	4.04E-06	BOD1,CPEB4
9q21.13	rs73477413	a	0.04	1.14	4.22E-06	TMEM2
9q34.11	rs7030440	a	0.34	1.04	4.54E-06	ASS1,FUB3
6q22.33	rs11154370	a	0.96	1.10	4.58E-06	RSPO3
8q24.22	rs28697871	g	0.99	1.23	4.61E-06	KHDRBS3
10q21.1	rs12763968	a	0.03	1.16	4.65E-06	PRKG1

7q31.31	rs77224217	g	0.95	1.11	4.67E-06	TSPAN12
4p16.3	rs35654957	c	0.37	1.05	4.73E-06	FGFRL1
4q26	rs7676164	a	0.70	1.04	4.82E-06	CAMK2D,ARSJ

Table S6B | Signals with $p<5x10^{-8}$ in the discovery followed for replication in 23andMe

						Discovery 37,857 cases 227,116 controls		Replication 147,200 cases 150,085 controls		Combined 185,057 cases 377,201 controls			
**Locus**	**Candidate Gene**	**Top SNP**	**Distance to Gene (kb)**	**EA**	**EAF**	**OR (95% CI)**	**P-value**	**OR (95% CI)**	**P-value**	**OR (95% CI)**	**P-value**	**N cases**	**I^2**
2q21.1	SLC8A1	rs11900510*	0	C	0.40	1.06 (1.03-1.08)	$4.7x10^{-08}$	1.02 (1.01-1.03)	$3.1x10^{-3}$	1.03 (1.02-1.04)	3.7x10-^{07}	164618	85
4p16.3	*FGFRL1*	rs111632154**	0	C	0.05	1.15 (1.09-1.20)	$4.2x10^{-08}$	1.05 (1.02-1.08)	$7.4x10^{-04}$	1.09 (1.04-1.09)	$2.4x10^{-08}$	164,618	83
		rs138081769*†		C	0.01	1.15 (1.10-1.22)	$2.7x10^{08}$	1.03 (1.00-1.06)	0.02	1.05 (1.03-1.04)	$5.5*10^{-06}$	164,618	
6q22.33	*RSPO3*	rs10457487	0.324	C	0.51	1.06 (1.05-1.08)	$2.3x10^{-15}$	1.04 (1.03-1.05)	$1.7x10^{-15}$	1.05 (1.04-1.06)	$4.8x10^{-28}$	185,057	5
		rs1930955*†		G	0.45	1.05 (1.03-1.06)	$1.9x10^{-08}$	1.01 (0.99-1.02)	0.19	1.02 (1.01-1.03)	$3.3*10^{-05}$	185,056	
6q25.1	*ESR1*	rs2982570	0	C	0.58	1.05 (1.04-1.07)	$8.1x10^{-12}$	1.03 (1.02-1.04)	$5.2x10^{-10}$	1.04 (1.03-1.05)	$4.5x10^{-19}$	185,057	23
7q31.31	*WNT16*	rs2908007	-18.99	A	0.60	1.08 (1.06-1.10)	$1.2x10^{-20}$	1.05 (1.04-1.06)	$5.6x10^{-22}$	1.06 (1.05-1.07)	$2.3x10^{-39}$	185,055	0
	CPED1		24.67										
7q31.31		rs2536189†		C	0.56	1.05 (0.03-1.06)	$1.9x10^{-08}$	1.03 (1.02-1.04)	$7.4x10^{-08}$	1.03 (1.02-1.04)	$3.9x10^{-14}$	185.056	
7q21.3	*C7orf76*	rs6465508	0	G	0.34	1.05 (1.03-1.07)	$4.0x10^{-09}$	1.04 (1.03-1.05)	$4.1x10^{-12}$	1.04 (1.03-1.05)	$2.0x10^{-19}$	185,056	35
	SHFM1		0										
7p21.1	*GRB10*	rs1548607	40.33	G	0.32	1.05 (1.03-1.07)	$3.2x10^{-08}$	1.02 (1.01-1.04)	$2.1x10^{-04}$	1.03 (1.02-1.05)	$4.7x10^{-10}$	185,052	40
	COBL		-182.4										
10q21.1	*MBL2*	rs11003047	-90.63	G	0.11	1.09 (1.07-1.12)	$6.2x10^{-12}$	1.08 (1.07-1.10)	$1.4x10^{-21}$	1.09 (1.07-1.10)	$9.5x10^{-33}$	185,057	0
17q21.31	*SOST*	rs2741856	-4.26	G	0.92	1.11 (1.08-1.14)	$2.4x10^{-12}$	1.08 (1.06-1.11)	$6.3x10^{-15}$	1.10 (1.07-1.11)	$3.1x10^{-25}$	184,977	0
	DUSP3		-16.65										
	MEOX1		87.52										
		rs4793022†		G	0.62	1.06 (1.04-1.08)	$7.3x10^{-12}$	1.04 (1.03-1.05)	$2.8x10^{-12}$	1.04 (1.03-1.05)	1.0x10-21	185056	
18p11.21	*FAM210A*	rs4635400	0	A	0.36	1.06 (1.04-1.07)	$1.5x10^{-12}$	1.03 (1.02-1.04)	$2.7x10^{-09}$	1.04 (1.03-1.05)	$1.1x10^{-18}$	185,057	22
	RNMT		-7.149										
21q22.2	*ETS2*	rs9980072	141.9	G	0.73	1.06 (1.04-1.08)	$8.4x10^{-12}$	1.03 (1.01-1.04)	$1.8x10^{-05}$	1.04 (1.03-1.05)	$3.4x10^{-13}$	185,057	36

*rs11900510 did not replicate
**rs11632154 had high heterogeneity and was tested using random effect and was no longer significant
† did not survive conditional analyses

Table S6C | Meta-analysis results across stages for SNPs that did not replicate in GENOMOS

					DISCOVERY									***REPLICATION***						
					GEFOS 20,439 cases 78,843 controls		***EPIC 2,926 cases 17,710 controls***		***UKBB 14,492 cases 139,9563 controls***		***POOLED 37,856 cases 227,116 controls***			***In silico 4,119 cases 6,219 controls***		***GENOMOS 12,694 cases 23,793 controls***		***COMBINED 61,785 cases 273,543 controls***		
**Locus**	**SNP**	**EA**	**NEA**	**EAF**	**OR**	**P-value**	**OR**	**P-value**	**OR**	**P-value**	**OR**	**P-value**	**I^2**	**OR**	**P-value**	**OR**	**P-value**	**OR**	**P-value**	**I^2**
2p16.2	rs2941584	t	c	0.3131	1.07	1.13E-07	0.98	0.56	1.00	0.72	1.03	0.003	45	1.03	0.41	1.05	6.74E-03	1.03	8.6x10-05	42
1p36.12	rs6426749	g	c	0.8288	1.09	5.30E-07	1.07	0.07	1.02	0.13	1.05	$2.1x10^{-6}$	16	1.05	0.31	1.04	0.12	1.05	2.1x10-07	2
10q11.21	rs11239303	a	g	0.9424	1.16	5.55E-07	0.94	0.33	1.01	0.78	1.06	0.002	55	0.95	0.69	1.03	0.53	1.05	3.1x10-03	38
1p36.12	rs10493013	t	c	0.8286	1.09	5.86E-07	1.07	0.08	1.03	0.08	1.05	$8.8x10^{-07}$	8	1.04	0.34	-	-	1.05	6.1x10-07	10
16p13.13	rs12102589	g	a	0.0332	1.19	6.08E-07	1.01	0.87	1.00	0.95	1.08	$8.3x10^{-04}$	30	1.01	0.85	0.97	0.45	1.05	9.0x10-03	18
9q34.11	rs7030440	a	g	0.3446	1.07	8.36E-07	1.04	0.26	1.02	0.12	1.04	$4.5x10^{-06}$	32	0.98	0.62	1.01	0.56	1.03	3.4x10-05	13
11q22.3	rs12365612	g	a	0.9697	1.21	8.79E-07	0.96	0.63	1.03	0.40	1.09	$3.0x10^{-04}$	22	1.04	0.71	0.97	0.55	1.06	4.6x10-03	33
6q22.33	rs6908008	g	a	0.399	1.06	1.97E-06	1.07	0.02	1.02	0.18	1.04	$3.7x10^{-06}$	39	1.05	0.13	-	-	1.04	1.1x10-06	34
6q22.33	rs12663110	c	t	0.3991	1.06	2.37E-06	1.07	0.03	1.01	0.20	1.04	$5.4x10^{-06}$	39	1.05	0.15	-	-	1.04	1.7x10-06	34
11q13.2	rs3781586	a	c	0.1551	1.08	2.99E-06	1.01	0.78	1.02	0.14	1.05	$4.2x10^{-05}$	27	0.98	0.63	-	-	1.04	1.1x10-04	23
10q21.3	rs10822590	t	a	0.9398	1.17	3.79E-06	0.98	0.80	1.01	0.67	1.06	0.004	40	1.14	0.13	-	-	1.06	1.6x10-03	34
2p16.2	rs4233949	g	c	0.6177	1.06	4.59E-06	0.98	0.51	1.02	0.09	1.03	$7.0x10^{-05}$	25	0.99	0.83	1.03	0.30	1.04	5.5x10-07	15
1p34.2	rs3157	g	a	0.4542	1.06	6.24E-06	1.02	0.60	1.02	0.10	1.03	$2.6x10^{-05}$	0	0.96	0.20	1.03	0.07	1.03	3.3x10-05	23
6q14.1	rs910679	t	c	0.4685	1.06	6.56E-06	1.00	0.98	1.00	0.67	1.02	0.009	28	0.97	0.26	1.01	0.70	1.02	0.03	5
14q32.12	rs2498827	g	a	0.4384	1.06	6.99E-06	1.04	0.18	1.02	0.09	1.04	$8.0x10^{-06}$	9	1.01	0.70	1.00	0.95	1.03	5.4x10-05	20
3q24	rs11720889	t	c	0.349	1.06	8.06E-06	1.00	0.93	0.97	0.03	1.01	0.169	42	0.98	0.53	0.99	0.50	1.01	0.42	44
4q22.1	rs13136331	t	c	0.3009	1.06	1.41E-05	1.01	0.67	1.01	0.26	1.03	$2.2x10^{-04}$	36	1.03	0.39	-	-	1.03	1.6x10-04	29
16p12.3	rs7499984	a	g	0.5952	1.06	1.81E-05	0.98	0.41	0.98	0.13	1.01	0.141	0	0.98	0.49	1.00	0.90	1.01	0.27	33
18p11.21	rs12955124	a	c	0.6831	1.06	2.03E-05	1.01	0.86	1.01	0.29	1.03	$5.1x10^{-04}$	0	1.03	0.52	-	-	1.03	4.1x10-04	0
3p26.3	rs6442271	g	a	0.0499	1.14	3.29E-05	1.01	0.84	1.00	0.99	1.05	0.009	50	0.92	0.25	1.11	0.06	1.05	5.8x10-03	36
7q11.23	rs17147106	g	a	0.9496	1.15	3.72E-05	1.05	0.51	1.01	0.83	1.06	0.004	0	0.99	0.90	1.01	0.76	1.05	0.01	9

Table S6D | Previously identified BMD-associated loci with risk of any type of fracture followed for replication in 23andMe and GENOMOS

					Discovery						Replication								
					GEFOS 20,439 cases 78,843 controls		*EPIC 2,926 cases 17,710 controls*		*UKBB 14,492 cases 139,9563 controls*		GENOMOS 16,573 cases 37,543 controls		*23andMe 147,200 cases 150,085 controls*		COMBINED			*GEFOS2 (Estrada et al. 2012)*
Locus	SNP	EA	NEA	EAF	OR	P-value	OR	P-value	OR	P-value	OR	P-value	OR	P-value	OR	P-value	I^2	*P-value*
2p16.2	rs4233949	G	C	0.62	1.06	4.6x10-06	0.98	0.51	1.02	0.09	1.05	6.5x10-04	1.04	8.9x10-11	1.04	2.6x10-16	15	*2.6x10-08*
4q22.1	rs6532023	G	T	0.66	1.03	0.01	1.02	0.52	0.99	0.54	1.08	5.2x10-07	1.03	7.2x10-06	1.03	8.9x10-09	0	*1.7x10-08*
7q21.3	rs4727338	G	C	0.34	1.08	3.2x10-09	1.02	0.49	1.03	7.3x10-03	1.06	1.1x10-04	1.04	4.6x10-12	1.04	2.8x10-22	32	*5.9x10-11*
10q21.1	rs1373004	T	G	0.11	1.06	2.5x10-03	1.09	0.08	1.11	8.7x10-09	1.12	1.1x10-06	1.09	1.2x10-22	1.09	1.6x10-36	0	*9.0x10-09*
11q13.2	rs3736228	T	C	0.15	1.08	3.4x10-05	0.99	0.89	1.03	0.03	1.05	0.05	1.07	2.8x10-18	1.06	1.8x10-22	0	*1.4x10-08*
18p11.21	rs4796995	G	A	0.37	1.09	5.5x10-10	1.05	0.08	1.04	0.01	1.06	3.9x10-04	1.03	2.9x10-09	1.04	3.3x10-21	9	*8.8x10-13*
1p36.12	rs6426749	G	C	0.83	1.09	4.5x10-07	1.07	0.07	1.02	0.13	1.03	0.14	1.02	3.3x10-3	1.03	3.2x10-08	2	*3.6x10-06*
1p36.12	rs7521902	A	C	0.24	1.05	0.002	1.06	0.10	1.03	0.03	1.07	0.04	1.02	7.2x10-04	1.03	1.4x10-07	0	*1.4x10-07*
3p22.1	rs430727	T	C	0.45	0.95	1.6x10-05	0.95	0.07	0.99	0.43	0.94	2.9x10-04	1.03	1.1x10-08	1.03	2.2x10-14	0	*2.9x10-07*
7p14.1	rs6959212	T	C	0.34	1.04	0.01	1.03	0.31	1.04	2.6x10-04	1.05	9.5x10-04	1.02	1.1x10-05	1.03	1.4x10-07	0	*7.2x10-05*
7p31.31	rs3801387	A	G	0.72	1.04	3.7X10-03	1.08	0.02	1.08	1.9X10-09	1.09	2.4X10-07	1.05	2.0x10-17	1.06	4.0x10-32	0	*2.7X10-07*
9q34.11	rs7851693	G	C	0.36	1.06	7.0x10-05	1.04	0.24	1.02	0.14	1.03	0.09	1.05	4.8x10-16	1.04	2.1x10-19	0	*3.5x10-05*
11p14.1	rs163879	T	C	0.67	1.04	0.01	1.01	0.63	1.03	0.03	1.06	1.7x10-04	1.00	0.50	1.02	6.2x10-04	22	*3.3x10-05*
14q32.12	rs1286083	T	C	0.82	0.94	7.6x10-05	0.93	0.04	0.98	0.24	0.96	0.05	1.05	3.1x10-14	1.05	2.7x10-18	22	*7.0x10-05*
17p21.31	rs4792909	G	T	0.61	1.07	9.7x10-07	1.08	0.01	1.05	2.0x10-05	1.07	9.5x10-03	1.04	2.9x10-12	1.05	6.6x10-23	13	*8.8x10-13*
17q21.31	rs227584	A	C	0.70	1.02	0.08	1.03	0.31	1.06	6.7x10-06	1.04	0.01	1.03	8.2x10-06	1.04	2.2x10-11	16	*4.1x10-05*

Table S6E | Fracture SNPs in association with FN-BMD and LS-BMD

Locus	SNP	EA	NEA	EAF	FN-BMD			LS-BMD		
					Beta	se	p-value	Beta	se	p-value
2p16.2	rs4233949	c	g	0.38	0.03	0.01	1.5x10-04	0.06	0.01	5.0x10-12
3p22.1	rs430727	t	c	0.48	-0.07	0.01	9.7x10-17	-0.06	0.01	2.9x10-09
6q22.33	rs10457487	a	c	0.49	0.02	0.01	4.1x10-03	0.02	0.01	6.0x10-02
6q25.1	rs2982570	t	c	0.42	0.06	0.01	7.5x10-14	0.07	0.01	2.21x10-14
7p12.1	rs1548607	a	g	0.70	0.02	0.01	5.9x10-02	0.05	0.01	7.5x10-05
7p14.1	rs6959212	t	c	0.32	-0.03	0.01	6.4x10-04	-0.08	0.01	6.9x10-17
7q21.3	rs6465508	a	g	0.67	0.08	0.01	5.1x10-20	0.07	0.01	1.6x10-15
7q31.31	rs2908007	a	g	0.61	-0.05	0.01	2.9x10-07	-0.04	0.01	2.2x10-05
9q34.11	rs7851693	c	g	0.64	0.05	0.01	3.1x10-08	0.02	0.01	5.8x10-02
10q21.1	rs11003047	t	g	0.89	0.06	0.01	7.4x10-06	0.07	0.01	1.2x10-07
11q13.2	rs3736228	t	c	0.16	-0.05	0.01	1.8x10-06	-0.08	0.01	1.6x10-11
14q32.12	rs1286083	t	c	0.81	-0.06	0.01	2.9x10-08	-0.07	0.01	1.7x10-11
17q21.31	rs2741856	c	g	0.08	0.09	0.02	4.7x10-08	0.10	0.02	3.0x10-08
18p11.21	rs4635400	a	g	0.37	-0.04	0.01	4.7x10-06	-0.03	0.01	6.4x10-04
21q22.2	rs9980072	a	g	0.26	0.02	0.01	1.0x10-02	0.04	0.01	1.4x10-04

Table S7 | Estimates of the Genetic Correlation Between Other Clinical Risk Factors and BMD

Trait or Disease*	FN BMD			LS BMD		
	**rg**	**se**	**p**	**rg**	**se**	**p**
Age at Menopause	0.05	0.05	0.33	0.04	0.05	0.44
Rheumatoid Arthritis	-0.03	0.04	0.56	-0.12	0.05	0.01
Inflammatory Bowel Disease	-0.08	0.05	0.07	-0.07	0.05	0.18
Homocysteine levels	-0.10	0.07	0.08	-0.07	0.06	0.29
Grip Strength	0.11	0.00	**0.006**	0.15	0.04	**0.0001**
Age of puberty	-0.08	0.03	0.03	-0.06	0.03	0.06
Fasting Glucose	0.15	0.07	0.03	0.14	0.07	0.03
Coronary Heart Disease	0.04	0.06	0.47	0.05	0.06	0.44
Type 2 Diabetes	0.20	0.06	**0.0005**	0.15	0.06	0.01
Vitamin D levels	0.07	0.27	0.79	-0.04	0.26	0.87

Genome-wide data was not available for Thyroid levels, Type 1 Diabetes and Milk Calcium Intake

Table S8 | Estimating the effect of 15 Fracture Related Risk Factors on Fracture Risk

Trait or Disease	Unit OR per	IVW fixed meta-analysis		Weighted Median		Penalised Weighted Median	
		**OR (95%CI)**	**P-value**	**OR (95%CI)**	**P-value**	**OR (95%CI)**	**P-value**
Decreased Femoral Neck BMD	SD	1.55 (1.48, 1.63)	1.5x10-68	1.53 (1.40, 1.68)	1.8x10-20	1.54 (1.40-1.69)	1.1x10-19
Decreased Lumbar Spine BMD	SD	1.43 (1.37, 1.50)	2.3x10-55	1.30 (1.19, 1.43)	2.6x10-08	1.36 (1.23-1.49)	8.7x10-10
Earlier Menopause	SD	1.10 (1.00, 1.21)	0.05	1.14 (0.99, 1.32)	0.07	1.14 (0.99-1.31)	0.08
Rheumatoid Arthritis *	OR	1.02 (0.99, 1.04)	0.14	1.01 (0.97, 1.05)	0.72	1.01 (0.97, 1.04)	0.67
Type 1 Diabetes *	OR	1.00 (0.99, 1.02)	0.57	1.00 (0.98, 1.02)	0.98	1.00 (0.98-1.02)	0.98
Inflammatory Bowel Disease *	OR	1.00 (0.99, 1.01)	0.92	1.00 (0.98, 1.03)	0.66	1.00 (0.98, 1.03)	0.67
Decreased Thyroid-stimulating hormone	SD	0.99 (0.94, 1.04)	0.78	1.00 (0.93, 1.08)	0.96	1.00 (0.93-1.08)	0.90
Increased Homocysteine	SD	0.98 (0.92,1.05)	0.6	0.94 (0.85,1.03)	0.17	0.94 (0.85,1.03)	0.17
Decreased Grip Strength	SD	2.14 (1.13, 4.04)	0.01	2.41 (1.13, 5.14)	0.02	3.00 (1.37, 6.57)	0.006
Late Puberty	SD	1.06 (1.00, 1.13)	0.04	1.03 (0.94-1.14)	0.43	1.03 (0.94-1.13)	0.46
Increased Fasting Glucose	SD	1.04 (0.97, 1.12)	0.24	1.11 (1.01-1.23)	0.03	1.12 (1.01-1.23)	0.03
Coronary Heart Disease *	OR	1.01 (0.97, 1.05)	0.76	1.00 (0.94, 1.06)	0.99	1.00 (0.94, 1.06)	0.93
Type 2 Diabetes *	OR	0.99 (0.96, 1.01)	0.37	1.00 (0.94, 1.06)	0.99	1.00 (0.94, 1.06)	0.93
Decreased Vitamin D	SD	0.84 (0.70, 1.02)	0.07	0.82 (0.65, 1.04)	0.11	0.82 (0.65, 1.04)	0.11
Decreased Milk Calcium Intake	Servings per day	1.01 (0.80-1.23)	0.94	NA	NA	N/A	N/A

*SD=standard deviation; OR=odd ratio; IVW = inverse-variance weighted

Table S9 | Associations of different risk factors with bone mineral density

Trait or Disease	Scale of beta SD of BMD per	Femoral Neck BMD				Lumbar Spine BMD			
		Inverse Variance-Weighted MR		*MR-Egger Regression*		*Inverse Variance-Weighted MR*		*MR-Egger Regression*	
		**Effect Estimate (95%CI)**	**P**	**Intercept (95%CI)**	**P**	**Effect Estimate (95%CI)**	**P**	**Intercept (95%CI)**	**P**
Earlier Menopause	year	-0.063 (-0.080, -0.047)	0.038	0.003 (-0.003, 0.009)	0.28	-0.018 (-0.033, -0.004)	0.01	-0.002 (-0.009, 0.004)	0.48
Rheumatoid Arthritis	log (OR)	0.012 (-0.009, 0.034)	0.263	-0.004 (-0.010, 0.002)	0.22	0.025 (0.000, 0.051)	0.05	-0.009 (-0.016, -0.001)	0.02
Type 1 Diabetes	log (OR)	0.003 (-0.008, 0.014)	0.589	0.002 (-0.004, 0.007)	0.57	0.008 (-0.006, 0.021)	0.26	0.002 (-0.005, 0.009)	0.59
Inflammatory Bowel Disease	log (OR)	-0.009 (-0.021, 0.002)	0.104	-0.001 (-0.004, 0.001)	0.31	0.002 (-0.011, 0.016)	0.72	0.001 (-0.003, 0.004)	0.66
Decreased Thyroid-Stimulating Hormone	SD	0.003 (-0.050, 0.055)	0.924	-0.009 (-0.026, 0.008)	0.29	-0.034 (-0.096, 0.027)	0.28	-0.017 (-0.037, 0.002)	0.08
Increased Homocysteine	SD	-0.044(-0.136, -0.048)	0.035	0.003(-0.017,0.025)	0.66	-0.021(-0.127,0.085)	0.69	-0.013(-0.038,0.012)	0.24
Decreased Grip Strength	kilogram(kg)	0.019 (-0.012, 0.050)	0.229	-0.006 (-0.037, 0.025)	0.73	0.040 (-0.005, 0.085)	0.09	-0.019 (-0.064, 0.026)	0.40
**Age of puberty**	**year**	**-0.071 (-0.109, 0.034)**	**0.0002**	**0.003 (-0.003, 0.008)**	**0.33**	-0.013 (-0.025, -0.001)	**0.04**	0.003 (-0.003, 0.009)	**0.28**
Increased Fasting Glucose	mmol/l	0.150 (0.047, 0.253)	0.004	0.004 (-0.002, 0.009)	0.21	0.089 (-0.031, 0.209)	0.15	0.003 (-0.004, 0.009)	0.39
Coronary Heart Disease	Log (OR)	0.002 (-0.04, 0.045)	0.913	0.002 (-0.009, 0.013)	0.71	0.002 (-0.040, 0.045)	0.91	0.002 (-0.009, 0.013)	0.71
Type 2 Diabetes	Log (OR)	0.030 (0.005, 0.055)	0.020	0.001 (-0.006, 0.008)	0.85	0.014 (-0.015, 0.043)	0.36	0.005 (-0.003, 0.013)	0.22
Decreased Vitamin D	SD	-0.098 (-0.271, 0.075)	0.268	0.040 (-0.040, 0.120)	0.17	-0.004 (-0.206, 0.197)	0.97	-0.01 (-0.104, 0.083)	0.68
Calcium Milk Intake	Serving/day	-0.012 (-0.149, 0.126)	0.86	N/A	N/A	0.023 (-0.164, 0.209)	0.81	N/A	N/A

One BMI SNP (rs1201687) was not present in GEFOS seq. As a proxy rs9581855 was used (r2=1, 1000G phase 1); Five homocysteine SNPs were not present in GEFOS seq and no proxies were found; *SD=standard deviation; N/A : not applicable

III. Supplementary figures

Supplementary Figure 1: Description of study design

Supplementary Figure 2: Regional association plots of top signals

Supplementary Figure 1 | Description of study design

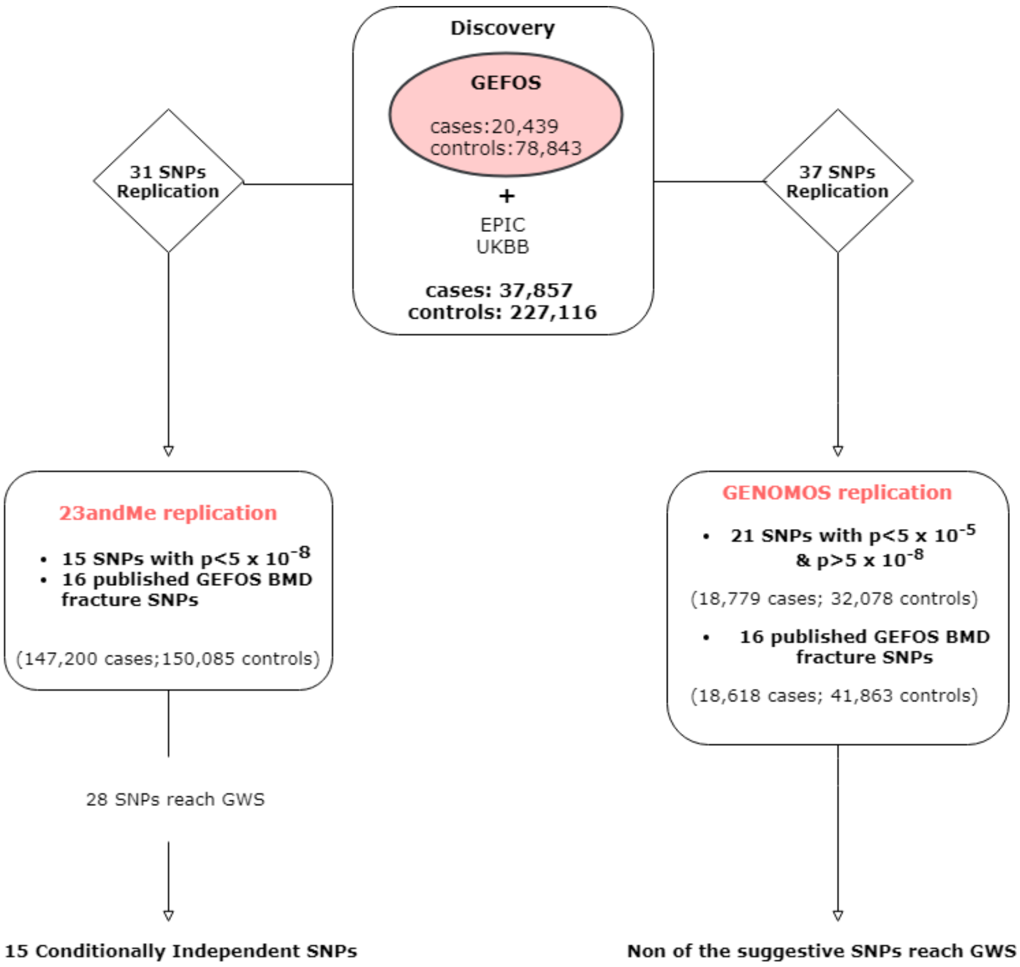

Supplementary Figure 2 | Regional association plots of top signals (A. 2p16.2 B. 3p22.1 C. 6q22.33, D. 6q31.31, E. 7q31.31, F. 7q21.3, G. 7q14.1, , H. 7q12.1, I. 9q34.11, J. 10q21.31, K. 11q13.2, L. 14q32.2, M. 17q21.31, N. 18q11.21, O. 21q22.2). SNPs are plotted by position in 500kb window against association with fracture risk (-log10 P). Plot highlighting the most significant SNPs in discovery fracture meta-analysis.

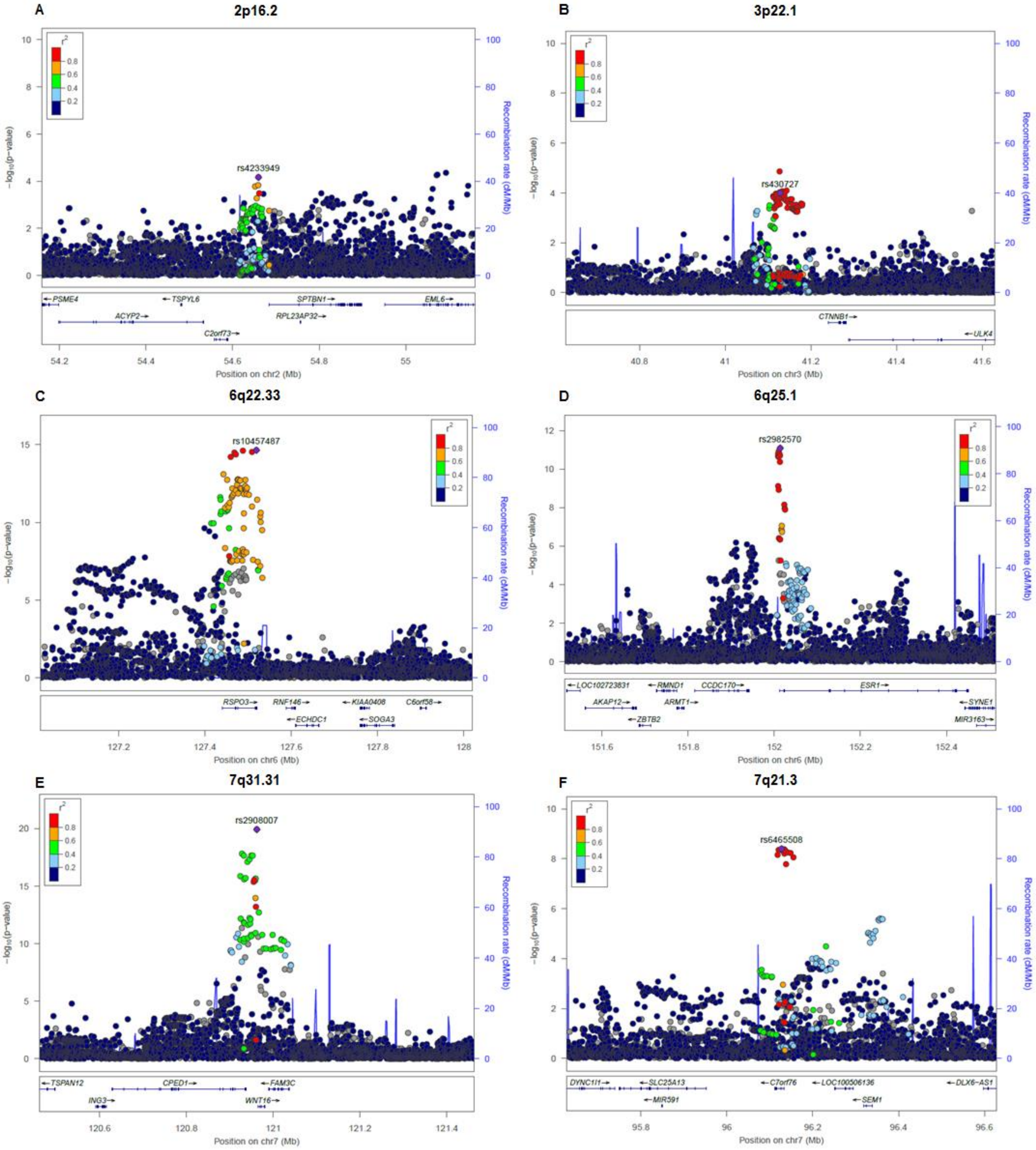

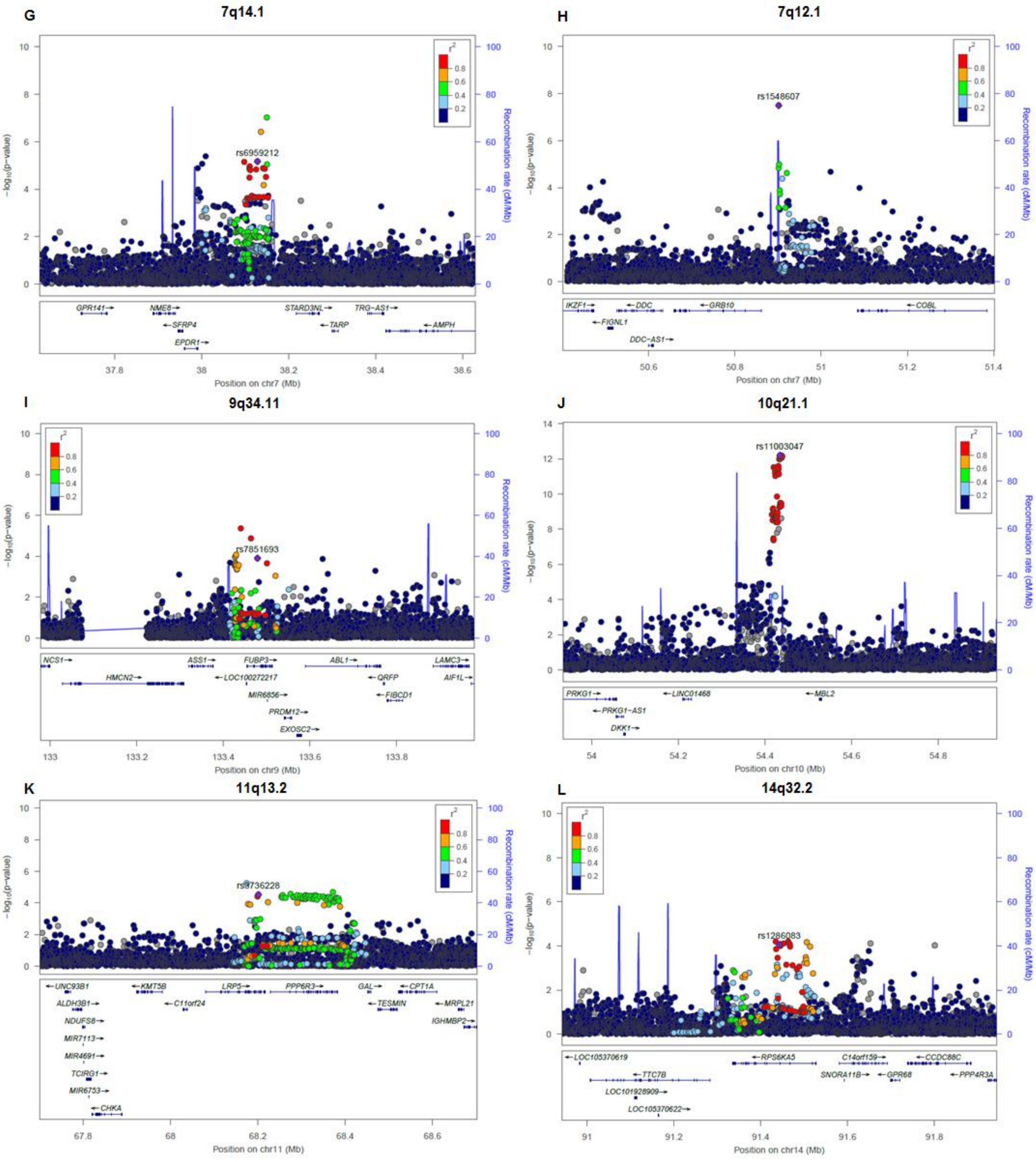
G
7q14.1
rs6959212
GPR141→
NME8→
←SFRP4
EPDR1→
STARD3NL→
←TARP
TRG−AS1→
←AMPH
Position on chr7 (Mb)
H
7q12.1
rs1548607
IKZF1→
←FIGNL1
←DDC
DDC−AS1→
←GRB10
←COBL
Position on chr7 (Mb)
I
9q34.11
rs7851693
NCS1→
HMCN2→
ASS1→
FUBP3→
←LOC100272217
MIR6856→
PRDM12→
EXOSC2→
ABL1→
←QRFP
←FIBCD1
LAMC3→
AIF1L→
Position on chr9 (Mb)
J
10q21.1
rs11003047
PRKG1→
←PRKG1−AS1
DKK1→
←LINC01468
←MBL2
Position on chr10 (Mb)
K
11q13.2
rs3736228
←UNC93B1
ALDH3B1→
NDUFS8→
MIR7113→
MIR4691→
TCIRG1→
MIR6753→
←CHKA
←KMT5B
←C11orf24
LRP5→
PPP6R3→
GAL→
←TESMIN
←CPT1A
←MRPL21
IGHMBP2→
Position on chr11 (Mb)
L
14q32.2
rs1286083
←LOC105370619
←TTC7B
LOC101928909→
LOC105370622→
←RPS6KA5
C14orf159→
SNORA11B→
←GPR68
←CCDC88C
←PPP4R3A
Position on chr14 (Mb)
−log10(p−value)
Recombination rate (cM/Mb)
r²

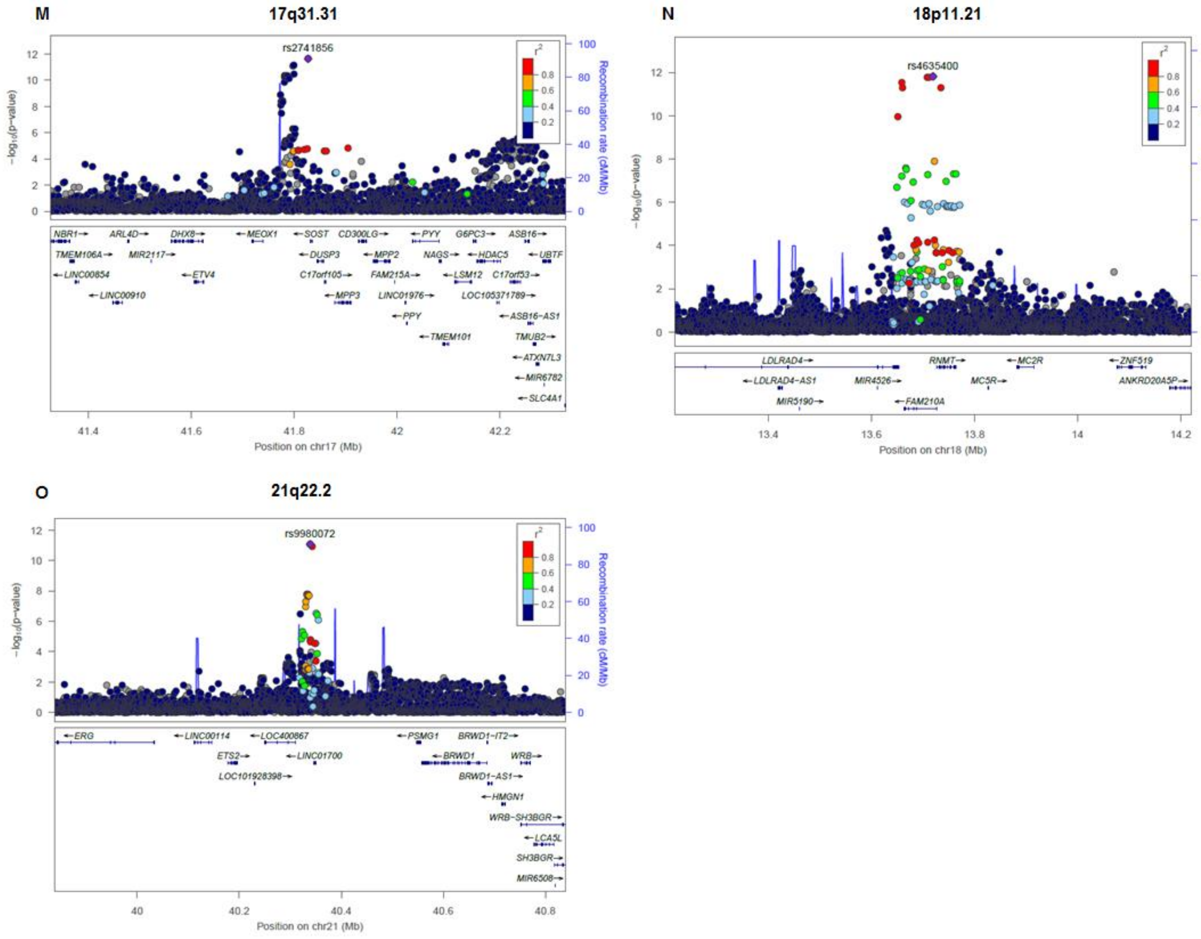

M
17q31.31
rs2741856
−log10(p-value)
Recombination rate (cM/Mb)
Position on chr17 (Mb)
NBR1 ARL4D DHX8 MEOX1 SOST CD300LG PYY G6PC3 ASB16
TMEM106A MIR2117 DUSP3 MPP2 NAGS HDAC5 UBTF
LINC00854 ETV4 C17orf105 FAM215A LSM12 C17orf53
LINC00910 MPP3 LINC01976 LOC105371789
PPY ASB16-AS1 TMEM101 TMUB2 ATXN7L3 MIR6782 SLC4A1
N
18p11.21
rs4635400
Position on chr18 (Mb)
LDLRAD4 RNMT MC2R ZNF519
LDLRAD4-AS1 MIR4526 MC5R ANKRD20A5P
MIR5190 FAM210A
O
21q22.2
rs9980072
Position on chr21 (Mb)
ERG LINC00114 LOC400867 PSMG1 BRWD1-IT2
ETS2 LINC01700 BRWD1 WRB
LOC101928398 BRWD1-AS1 HMGN1 WRB-SH3BGR LCA5L SH3BGR MIR6508

IV. Full acknowledgements

Cohort	Funding agencies	Full Acknowledgments
**AGES**	NIH contract N01-AG-12100, the NIA Intramural Research Program, Hjartavernd (the Icelandic Heart Association), and the Althingi (the Icelandic Parliament). Icelandic Heart Association .	The Age, Gene/Environment Susceptibility Reykjavik Study has been funded by NIH contract N01-AG-12100, the NIA Intramural Research Program, Hjartavernd (the Icelandic Heart Association), and the Althingi (the Icelandic Parliament). The Fracture Registry is funded by the Icelandic Heart Association .
**AOGC**	National Health and Medical Research Council (Australia) (grant reference 511132). Australian Cancer Research Foundation and Rebecca Cooper Foundation (Australia). National Health and Medical Research Council (Australia). National Health and Medical Research Council (Australia) Career Development Award (569807). Medical Research Council New Investigator Award (MRC G0800582). Health Research Council of New Zealand. Sanofi-Aventis, Eli Lilly, Novartis, Pfizer, Proctor&Gamble Pharmaceuticals and Roche. National Health and Medical Research Council, Australia. Australian National Health and Medical Research Council, MBF Living Well foundation, the Ernst Heine Family Foundation and from untied educational grants from Amgen, Eli Lilly International, GE-Lunar, Merck Australia, Novartis, Sanofi-Aventis Australia and Servier. Medical research Council UK & Arthritis Research UK. The Victorian Health Promotion Foundation and the Geelong Region Medical Research Foundation, and the National Health and Medical Research Council, Australia (project grant 628582). Action Research UK. David M Evans is supported by an Australian Research Council Future Fellowship (FT130101709). This work was supported by a Medical Research Council Programme Grant (MC_UU_12013/4).	We would like to thank all participants who provided DNA and clinical information for the AOGC. The AOGC was funded by the Australian National Health and Medical Research Council (Australia) (grant reference 511132). Funding was also received from the Australian Cancer Research Foundation and Rebecca Cooper Foundation (Australia). MAB is funded by a National Health and Medical Research Council (Australia) Principal Research Fellowship and ELD is funded by a National Health and Medical Research Council (Australia) Career Development Award (569807). DME is supported by a Medical Research Council New Investigator Award (MRC G0800582) and JPK is funded by a Wellcome Trust PhD studentship (WT083431MA). IR is supported by the Health Research Council of New Zealand. The Sydney Twin Study and Tasmanian Older Adult Cohort were supported by the National Health and Medical Research Council, Australia. The help of Dr Joanna Makovey from the Sydney Twin Study is gratefully acknowledged. The Dubbo Osteoporosis Epidemiology Study was supported by the Australian National Health and Medical Research Council, MBF Living Well foundation, the Ernst Heine Family Foundation and from untied educational grants from Amgen, Eli Lilly International, GE-Lunar, Merck Australia, Novartis, Sanofi-Aventis Australia and Servier. The contribution from A/Prof Jacqueline R Center, A/Prof Tuan V Nguyen, Mr Sing C Nguyen, Ms Denia Mang, and Ms Ruth Toppler is gratefully acknowledged. The OPUS study was supported by Sanofi-Aventis, Eli Lilly, Novartis, Pfizer, Proctor&Gamble Pharmaceuticals and Roche. We thank the co-principal investigators from OPUS (Dr Christian Roux, Dr David Reid, Dr Claus Glüer, and Dr Dieter Felsenber); Ms Judith Finigan and Ms Selina Simpson. The assistance of Ms Fatma Gossiel (Sheffield) is gratefully acknowledged. The Hertfordshire Cohort Study was supported by grants from the Medical research Council UK & Arthritis Research UK. We thank the men and women who participated; Prof Cyrus Cooper; and the Herfordshire GPs who supported the study. The Oxford Osteoporosis Study was funded by Action Research, UK. The AOGC thanks the research nurses involved in this study (Ms Barbara Mason and Ms Amanda Horne (Auckland), Ms Linda Bradbury and Ms Kate Lowings (Brisbane), Ms Katherine Kolk and Ms Rumbidzai Tichawangana (Geelong); Ms Helen Steane (Hobart); Ms Jemma Christie (Melbourne); and Ms Janelle Rampellini (Perth)). The AOGC also acknowledges gratefully technical support from Ms Kathryn Addison, Ms Marieke Brugmans, Ms Catherine Cremen, Ms Johanna Hadler, and Ms Karena Pryce.). Finally, we further want to acknowledge AOGC principal investigators Eugene McCloskey, Geoffrey C Nicholson, Richard Eastell, Richard L Prince, John A Eisman, Graeme Jones, Ian R Reid, Elaine M Dennison, and John Wark.
**BPROOF**	This study is supported and funded so far by The Netherlands Organization for Health Research and Development (ZonMw, Grant 6130.0031), the Hague; unrestricted grant from NZO (Dutch Dairy Association), Zoetermeer; Orthica, Almere; NCHA (Netherlands Consortium Healthy Ageing) Leiden/Rotterdam; Ministry of Economic Affairs, Agriculture and Innovation (project KB-15-004-003), the Hague; Wageningen University, Wageningen; VUmc, Amsterdam; Erasmus Medical Center, Rotterdam.	We thank the participants of the B-PROOF study for their enthusiasm and cooperation. Furthermore, we thank the dedicated research team that is conducting the study.

Cohort	Funding agencies	Full Acknowledgments
**CHS**	National Heart Lung and Blood Institute (NHLBI) contracts HHSN268201200036C, HHSN268200800007C, N01HC55222, N01HC85079, N01HC85080, N01HC85081, N01HC85082, N01HC85083, N01HC85086; and NHLBI grants U01HL080295, R01HL087652, R01HL105756, R01HL103612, R01HL120393, and R01HL130114 with additional contribution from the National Institute of Neurological Disorders and Stroke (NINDS). Additional support was provided through R01AG023629 from the National Institute on Aging (NIA). Genotyping supported in part by the National Center for Advancing Translational Sciences, CTSI grant UL1TR000124, and the National Institute of Diabetes and Digestive and Kidney Disease Diabetes Research Center (DRC) grant DK063491 to the Southern California Diabetes Endocrinology Research Center.	Cardiovascular Health Study: This CHS research was supported by NHLBI contracts HHSN268201200036C, HHSN268200800007C, N01HC55222, N01HC85079, N01HC85080, N01HC85081, N01HC85082, N01HC85083, N01HC85086; and NHLBI grants U01HL080295, R01HL087652, R01HL105756, R01HL103612, R01HL120393, and R01HL130114 with additional contribution from the National Institute of Neurological Disorders and Stroke (NINDS). Additional support was provided through R01AG023629 from the National Institute on Aging (NIA). A full list of principal CHS investigators and institutions can be found at CHS-NHLBI.org. The provision of genotyping data was supported in part by the National Center for Advancing Translational Sciences, CTSI grant UL1TR000124, and the National Institute of Diabetes and Digestive and Kidney Disease Diabetes Research Center (DRC) grant DK063491 to the Southern California Diabetes Endocrinology Research Center.The content is solely the responsibility of the authors and does not necessarily represent the official views of the National Institutes of Health.
**deCODE**	deCODE Genetics	Funded by deCODE Genetics. We wish to thank the study volunteers for their contributions to this project, and the staff at the deCODE core facilities.
**EGCUT**	This study was supported by EU H2020 grants 692145, 676550, 654248, Estonian Research Council Grant IUT20-60, NIASC and EIT – Health and EU through the European Regional Development Fund (Project No. 2014-2020.4.01.15-0012 GENTRANSMED).	EGC authors want to acknowledge EGCUT personnel, especially Mr. Steven Smit. The genotyping of the EGCUT samples were performed in Estonian Biocentre Genotyping Core Facility, EGC authors want toacknowledge Mr. Viljo Soo for their contribution in that. EGCUT data analyzes were carried out in part in the High Performance Computing Center of University of Tartu.
**EPIC-Norfolk**	The UK's NIMR Biomedical Research Centre Grant. Cancer Research Campaign; Medical Research Council; Stroke Association; British Heart Foundation; Department of Health; Europe Against Cancer Programme Commission of the European Union and the Ministry of Agriculture, Fisheries and Food.	The UK's NIMR Biomedical Research Centre Grant to Cambridge contributed to the costs of genotyping. EPIC-Norfolk is funded by Cancer Research Campaign; Medical Research Council; Stroke Association; British Heart Foundation; Department of Health; Europe Against Cancer Programme Commission of the European Union and the Ministry of Agriculture, Fisheries and Food. The funders had no role in study design, data collection and analysis, decision to publish, or preparation of the manuscript.EPIC-Norfolk is funded by Cancer Research Campaign; Medical Research Council; Stroke Association; British Heart Foundation; Department of Health; Europe Against Cancer Programme Commission of the European Union and the Ministry of Agriculture, Fisheries and Food. The UK's NIMR Biomedical Research Centre Grant to Cambridge contributed to the costs of genotyping. We thank in particular Mrs N Dalzell for coordination and DXA data collection, Robert Luben for data management, Serena Scollen and Alison Dunning for genotyping in GENOMOS, and Matt Sims and Steve Knighton for managing the EPIC DNA bank.
**ERF**	Netherlands Organisation for Scientific Research (NWO), Erasmus university medical center, the Centre for Medical Systems Biology (CMSB1 and CMSB2) of the Netherlands Genomics Initiative (NGI).	The ERF study was supported by grants from the Netherlands Organisation for Scientific Research (NWO), Erasmus university medical center, the Centre for Medical Systems Biology (CMSB1 and CMSB2) of the Netherlands Genomics Initiative (NGI). We are grateful to all individuals and their relatives and general practitioners for their contributions and Jeannette Vergeer for the supervision of the laboratory work.
**Framingham Study Original Cohort, Framingham Offspring Study**	National Institute for Arthritis, Musculoskeletal and Skin Diseases and National Institute on Aging (R01 AR/AG 41398; DPK and R01 AR 050066; DK National Heart, Lung, and Blood Institute's Framingham Heart Study (N01-HC-25195) and its contract with Affymetrix, Inc. for genotyping services (N02-HL-6-4278).	The study was funded by grants from the US National Institute for Arthritis, Musculoskeletal and Skin Diseases and National Institute on Aging (R01 AR 41398; DPK and R01 AR 050066; DK). The Framingham Heart Study of the National Heart, Lung, and Blood Institute of the National Institutes of Health and Boston University School of Medicine were supported by the National Heart, Lung, and Blood Institute's Framingham Heart Study (N01-HC-25195) and its contract with Affymetrix, Inc. for genotyping services (N02-HL-6-4278). Analyses reflect intellectual input and resource development from the Framingham Heart Study investigators participating in the SNP Health Association Resource (SHARe) project. A portion of this research was conducted using the Linux Cluster for Genetic Analysis (LinGA-II) funded by the Robert Dawson Evans Endowment of the Department of Medicine at Boston University School of Medicine and Boston Medical Center.

Cohort	Funding agencies	Full Acknowledgments
**GOOD**	Swedish Research Council (K2010-54X-09894-19-3, 2006-3832 and K2010-52X-20229-05-3), the Swedish Foundation for Strategic Research, the ALF/LUA research grant in Gothenburg, the Lundberg Foundation, the Torsten and Ragnar Söderberg's Foundation, the Västra Götaland Foundation, the Göteborg Medical Society, the Novo Nordisk foundation, and the European Commission grant HEALTH-F2-2008-201865-GEFOS.	Financial support was received from the Swedish Research Council (K2010-54X-09894-19-3, 2006-3832 and K2010-52X-20229-05-3), the Swedish Foundation for Strategic Research, the ALF/LUA research grant in Gothenburg, the Lundberg Foundation, the Torsten and Ragnar Söderberg's Foundation, the Västra Götaland Foundation, the Göteborg Medical Society, the Novo Nordisk foundation, and the European Commission grant HEALTH-F2-2008-201865-GEFOS. We would like to acknowledge Maria Nethander at the genomics core facility at University of Gothenburg for statistical analyses. We would also like to thank Dr. Tobias A. Knoch, Luc V. de Zeeuw, Anis Abuseiris, and Rob de Graaf as well as their institutions the Erasmus Computing Grid, Rotterdam, The Netherlands, and especially the national German MediGRID and Services@MediGRID part of the German D-Grid, both funded by the German Bundesministerium fuer Forschung und Technology under grants #01 AK 803 A-H and # 01 IG 07015 G for access to their grid resources. We would also like to thank Karol Estrada, Department of Internal Medicine, Erasmus MC, Rotterdam, Netherlands for advice regarding the grid resources.
**HealthABC**	The Intramural Research Program of the NIH, National Institute on Aging. U.S. National Institute of Aging (NIA) contracts N01AG62101, N01AG62103, and N01AG62106. NIA grant 1R01AG032098. The Center for Inherited Disease Research (CIDR). National Institutes of Health contract number HHSN268200782096C.	This research was supported in part by the Intramural Research Program of the NIH, National Institute on Aging. This research was supported by the U.S. National Institute of Aging (NIA) contracts N01AG62101, N01AG62103, and N01AG62106. The genome-wide association study was funded by NIA grant 1R01AG032098 to Wake Forest University Health Sciences and genotyping services were provided by the Center for Inherited Disease Research (CIDR). CIDR is fully funded through a federal contract from the National Institutes of Health to The Johns Hopkins University, contract number HHSN268200782096C.We would like to thank the participants of the Health, Aging, and Body Composition Study.
**HKOS**	Hong Kong Research Grant Council (HKU 768610M); The Bone Health Fund of HKU Foundation; The KC Wong Education Foundation; Small Project Funding (201007176237); Matching Grant, CRCG Grant and Osteoporosis and Endocrine Research Fund, and the Genomics Strategic Research Theme of The University of Hong Kong.	This project is supported by Hong Kong Research Grant Council (HKU 768610M); The Bone Health Fund of HKU Foundation; The KC Wong Education Foundation; Small Project Funding (201007176237); Matching Grant, CRCG Grant and Osteoporosis and Endocrine Research Fund, and the Genomics Startegic Research Theme of The University of Hong Kong. The authors are very grateful to the participants and clinical staff at the Queen Mary Hospital of The University of Hong Kong.
**MrOS**	The Osteoporotic Fractures in Men (MrOS) Study is supported by National Institutes of Health funding. The following institutes provide support: the National Institute of Arthritis and Musculoskeletal and Skin Diseases (NIAMS), the National Institute on Aging (NIA), the National Center for Research Resources (NCRR), and NIH Roadmap for Medical Research under the following grant numbers: U01 AR45580, U01 AR45614, U01 AR45632, U01 AR45647, U01 AR45654, U01 AR45583, U01 AG18197, U01-AG027810, and UL1 RR024140.	We thank all the participants in the Osteoporotic Fractures in Men Study for their continued participation.
**PROSPER/PHASE study**	European Union's Seventh Framework Programme (FP7/2007-2013) under grant agreement n° HEALTH-F2-2009-223004 PHASE.	The research leading to these results has received funding from the European Union's Seventh Framework Programme (FP7/2007-2013) under grant agreement n° HEALTH-F2-2009-223004 PHASE.
**SOF**	The Study of Osteoporotic Fractures (SOF) is supported by National Institutes of Health funding. The National Institute on Aging (NIA) and the National Institute of Arthritis and Musculoskeletal and Skin Diseases(NIAMS) provides support under the following grant numbers: R01 AG005407, R01 AR35582, R01 AR35583, R01 AR35584, R01 AG005394, R01 AG027574, R01 AG027576, and R01 AG026720.	We thank the participants and staff who made SOF possible
**RSI, RSII, RSIII**	Netherlands Organisation of Scientific Research NWO Investments (nr. 175.010.2005.011, 911-03-012); Research Institute for Diseases in the Elderly (014-93-015; RIDE2); Netherlands Genomics Initiative/Netherlands Consortium for Healthy Aging (050-060-810); German Bundesministerium fuer Forschung und Technology under grants #01 AK 803 A-H and # 01 IG 07015 G.	The GWA study was funded by the Netherlands Organisation of Scientific Research NWO Investments (nr. 175.010.2005.011, 911-03-012), the Research Institute for Diseases in the Elderly (014-93-015; RIDE2), the Netherlands Genomics Initiative (NGI)/Netherlands Consortium for Healthy Aging (NCHA) project nr. 050-060-810. We thank Pascal Arp, Mila Jhamai, Dr Michael Moorhouse, Marijn Verkerk, and Lisbeth Herrera for their help in creating the GWAS database. We also thank Lisette Stolk for her support creating the fracture dataset and members of the GIANT consortium for sharing scripts used in this study. The Rotterdam Study is funded by Erasmus Medical Center and Erasmus University, Rotterdam, Netherlands Organization for the Health Research and Development (ZonMw), the Research Institute for Diseases in the Elderly (RIDE), the Ministry of

Cohort	Funding agencies	Full Acknowledgments
		Education, Culture and Science, the Ministry for Health, Welfare and Sports, the European Commission (DG XII), and the Municipality of Rotterdam. The authors are very grateful to the participants and staff from the Rotterdam Study, particularly L. Buist and J.H. van den Boogert and also the participating general practitioners and pharmacists. We would like to thank Dr. Tobias A. Knoch, Luc V. de Zeeuw, Anis Abuseiris, and Rob de Graaf as well as their institutions the Erasmus Computing Grid, Rotterdam, The Netherlands, and especially the national German MediGRID and Services@MediGRID part of the German D-Grid, both funded by the German Bundesministerium fuer Forschung und Technology under grants #01 AK 803 A-H and # 01 IG 07015 G for access to their grid resources.
**TUK1, TUK23**	NIHR Biomedical Research Centre (grant to Guys' and St. Thomas' Hospitals and King's College London); the Chronic Disease Research Foundation; Wellcome Trust ; Canadian Institutes of Health Research, the Canadian Foundation for Innovation, the Fonds de la Recherche en Santé Québec, The Lady Davis Institute, the Jewish General Hospital and Ministère du Développement économique, de l'Innovation et de l'Exportation du Quebec.	NIHR Biomedical Research Centre (grant to Guys' and St. Thomas' Hospitals and King's College London); the Chronic Disease Research Foundation; Canadian Institutes of Health Research, the Canadian Foundation for Innovation, the Fonds de la Recherche en Santé Québec, The Lady Davis Institute, the Jewish General Hospital and Ministère du Développement économique, de l'Innovation et de l'Exportation du Quebec. Dr. Frances Williams for her contributions to phenotyping in this cohort.
**WGHS**	The WGHS is supported by the National Heart, Lung, and Blood Institute (HL043851 and HL080467) and the National Cancer Institute (CA047988 and UM1CA182913), the Donald W. Reynolds Foundation, and the Fondation Leducq, with collaborative scientific support and funding for genotyping provided by Amgen	The WGHS is supported by the National Heart, Lung, and Blood Institute (HL043851 and HL080467) and the National Cancer Institute (CA047988 and UM1CA182913), the Donald W. Reynolds Foundation, and the Fondation Leducq, with collaborative scientific support and funding for genotyping provided by Amgen
**WHI**	The WHI program is funded by the National Heart, Lung, and Blood Institute, National Institutes of Health, U.S. Department of Health and Human Services through contracts N01WH22110, 24152, 32100-2, 32105-6, 32108-9, 32111-13, 32115, 32118-32119, 32122, 42107-26, 42129-32, and 44221.	We thank the institutes, centers and investigators of the Women's Health Initiative (WHI) who developed and implemented the recruitment of participants, and the clinical and laboratory studies for WHI.
**Young Finns study (YFS)**	The Young Finns Study has been financially supported by the Academy of Finland: grants 286284, 134309 (Eye), 126925, 121584, 124282, 129378 (Salve), 117787 (Gendi), and 41071 (Skidi); the Social Insurance Institution of Finland; Competitive State Research Financing of the Expert Responsibility area of Kuopio, Tampere and Turku University Hospitals (grant X51001); Juho Vainio Foundation; Paavo Nurmi Foundation; Finnish Foundation for Cardiovascular Research ; Finnish Cultural Foundation; Tampere Tuberculosis Foundation; Emil Aaltonen Foundation; Yrjö Jahnsson Foundation; Signe and Ane Gyllenberg Foundation; Diabetes Research Foundation of Finnish Diabetes Association; and EU Horizon 2020 (grant 755320 for TAXINOMISIS).	The Young Finns Study has been financially supported by the Academy of Finland: grants 286284 (T.L), 134309 (Eye), 126925, 121584, 124282, 129378 (Salve), 117787 (Gendi), and 41071 (Skidi); the Social Insurance Institution of Finland; Competitive State Research Financing of the Expert Responsibility area of Tampere, Turku and Kuopio University Hospitals (grant X51001); Juho Vainio Foundation; Paavo Nurmi Foundation; Finnish Foundation for Cardiovascular Research; Finnish Cultural Foundation; Tampere Tuberculosis Foundation; Emil Aaltonen Foundation; Yrjö Jahnsson Foundation; Signe and Ane Gyllenberg Foundation; and Diabetes Research Foundation of Finnish Diabetes Association. The expert technical assistance in the statistical analyses by Irina Lisinen is gratefully acknowledged.
**23andMe**		We thank the customers of 23andMe who consented to participate in research and answered surveys. We also thank all the employees of 23andMe, who together have made this research possible**.**
**APOS**S		We are extremely grateful for the hard work of the clinical research staff in the Musculoskeletal Research Programme and to all the women who kindly participated in the study. Sample collection and analysis was supported in part by grants from the European Commission (QLRT-2001-02629) and the UK Food Standards Agency.
**AROS**		The authors are very grateful to the participants and staff from the AROS Study.
**BARCOS**	Red de Envejecimiento y fragilidad RETICEF, CIBERER, Instituto Carlos III. Fondos FEDER. Fondo de Investigación Sanitaria (FIS PI13/00116). Spanish Ministry of Education and Science (SAF2014-56562-R), Catalan Government (2014SGR932).	Red de Envejecimiento y fragilidad RETICEF, CIBERER, Instituto Carlos III. Fondos FEDER. Fondo de Investigación Sanitaria (FIS PI13/00116). Spanish Ministry of Education and Science (SAF2014-56562-R). Catalan Government (2014SGR932).

Cohort	Funding agencies	Full Acknowledgments
**Austrios-A Austrios-B**	This work was supported by BioPersMed (COMET K project 825329), and the Competence Center CBmed (COMET K1 centre 844609), funded by the Austrian Federal Ministry of Transport, Innovation and Technology (BMVIT) and the Austrian Federal Ministry of Economics and Labour/the Federal Ministry of Economy, Family and Youth (BMWA/BMWFJ) and the Styrian Business Promotion Agency (SFG).	
**Cabrio-C, Cabrio-CC**	Instituto de Salud Carlos III-Fondo de Investigaciones Sanitarias Grants PI 06/34,PI09/539, PI12/615 and PI15/521 (that could be confunded by European Union-FEDER funds).	Instituto de Salud Carlos III-Fondo de Investigaciones Sanitarias (PI 06/34,PI09/539, PI12/615,PI15/521).We acknowledge the inputs and help of D. Nan, J. Castillo, M.I. Pérez-Núñez and the staff of the Centro de Salud Jose Barros. We also thank the technical assistance of C. Sañudo and J. Arozamena. C. Sañudo was funded by Instituto de Inbestigación Valdecilla (IDIVAL)
**CAIFOS**	Healthway Health Promotion Foundation of Western Australia, Australasian Menopause Society and the Australian National Health and Medical Research Council Project Grant (254627, 303169 and 572604).	Healthway Health Promotion Foundation of Western Australia, Australasian Menopause Society and the Australian National Health and Medical Research Council Project Grant (254627, 303169 and 572604).This CAIFOS study was supported by the Healthway Health Promotion Foundation of Western Australia, Australasian Menopause Society and the Australian National Health and Medical Research Council Project Grant (254627, 303169 and 572604). The salary of Dr Lewis is suported by a National Health and Medical Research Council of Australia Career Development Fellowship.
**Calex-family**		Mr. Leiting Xu, Ms. Shumei Cheng and Professor Marku Alen.
**CaMos**	CaMos was supported by a grant from the Canadian Institutes for Health Research(CIHR)(grant number MOP111103)	We thank all participants for having given their consent to the study.
**DOES (Dubbio)**	The Dubbo Osteoporosis Epidemiology Study was supported by the Australian National Health and Medical Research Council, MBF Living Well foundation, the Ernst Heine Family Foundation and from untied educational grants from Amgen, Eli Lilly International, GE-Lunar, Merck Australia, Novartis, Sanofi-Aventis Australia and Servier. The OPUS study was supported by Sanofi-Aventis, Eli Lilly, Novartis, Pfizer, Proctor&Gamble Pharmaceuticals and Roche.	
**DOPS**		The authors are very grateful to the participants and staff from the DOPS Study. We would like to thank Prof. Leif Mosekilde, Prof. Kim Brixen, Dr. Jens-Erik Beck Jensen and Dr. Pia Eiken as well as their institutions.
**EDOS**	The EDOS study was supported by a grant from Arthritis Research UK (grant number 15389).	The EDOS study was supported by a grant from Arthritis Research UK (grant number 15389).
**EPOS**		EU Biomed 1 (BMHICT920182, CIPDCT925012, ERBC1PDCT 940229, ERBC1PDCT930105), Medical Research Council G9321536 and G9800062, Wellcome Trust collaborative Research Initiative 1995, MAFF AN0523,EU FP5 (QLK6-CT-2002-02629), Food Standards Agency N05046, GEFOS EU FP7 Integrated Project Grant Reference: 201865 The UK's NIMR Biomedical Research Centre Grant to Cambridge contributed to the costs of genotyping.The European Prospective Osteoporosis Study (1992-) was the prospective phase of the European Vertebral Osteoporosis Study, initiated by Alan J Silman who continued as the joint coordinator of EPOS. Particular thank are due to Terry O'Neill, Mark Lunt and Joe Finn for managing the collection of phenotypic data in Manchester and Jon Power for lab support. The PIs of the EPOS Centres contributing to the GENOMOS initiative and their excellent and enthusiastic staff are thanked for their energetic contributions to local fund-raising and excellent data collection efforts as well as collecting blood for DNA from their subjects: J Bruges Armas, A Bhalla, G Poor, R Nuti, A Lopes Vaz, L Benevolenskaya, S Grazio, K Weber, T Miazgowski, J J Stepan, J da Silva, P Masaryk, F Galan Galan, S Boonen, S Havelka, G Lyritis, M Naves Diaz, R Perez Cano .
**GEOS**	Canadian Institutes for health research operating grant funding reference #86748.	Financial support was provided by the Canadian Institutes for Health Research(Funding reference #86748). Infrastructure support to the various research centers was provided by the Fonds de Recherche en Santé du Quebec. François Rousseau holds a Fonds de la Recherche en Santé du Québec / MSSS Research Chair in Health Technology Assessment and Evidence Based Laboratory Medicine. We thank Cynthia Roy and Johanne Bussières for technical assistance.

Cohort	Funding agencies	Full Acknowledgments
**GROS SUDY**	University of Athens, Greece (Kapodistrias 2009) .	The GROS study was supported partially by a research grant (Kapodistrias 2009, University of Athens, Greece). We would like to thank the participants and staff from the Department of Orthopaedics, University Hospital of Thessalia, Larissa, Greece. The authors are very grateful to Mrs. Samara S. and Mr. Chassanidis C.
**HCS**	The study was supported by the MRC of the United Kingdom; Arthritis Research UK; NIHR Musculoskeletal BRU Oxford; NIHR Nutrition BRC Southampton'	Medical Research Council; Arthritis Research UK; NIHR Musculoskeletal BRU Oxford; NIHR Nutrition BRU Southampton.
**Hong Kong**	The projects have been supported by The Hong Kong Jockey Club Charities Trust, VC discretionary fund of The Chinese University of Hong Kong and Research Grants Council Earmarked Grant CUHK4101/02M.	The projects have been supported by The Hong Kong Jockey Club Charities Trust, VC discretionary fund of The Chinese University of Hong Kong and Research Grants Council Earmarked Grant CUHK4101/02M.
**KorAMC**	A grant of the Korea Health Technology R&D Project, the Ministry of Health & Welfare, Republic of Korea (Project No.: HI14C2258); A grant of the Korea Health Technology R&D Project, the Ministry of Health & Welfare, Republic of Korea (Project No.: HI15C0377)	A grant of the Korea Health Technology R&D Project, the Ministry of Health & Welfare, Republic of Korea (Project No.: HI14C2258), and by a grant of the Korea Health Technology R&D Project, the Ministry of Health & Welfare, Republic of Korea (Project No.: HI15C0377)
**LASA**	The Longitudinal Aging Study Amsterdam is largely supported by a grant from the Netherlands Ministry of Health Welfare and Sports, Directorate of Long-Term Care.	The Longitudinal Aging Study Amsterdam is largely supported by a grant from the Netherlands Ministry of Health Welfare and Sports, Directorate of Long-Term Care.
**MOFS**	Financial support was received from the European Union Strategic Educational Pathways Scholarhip scheme (STEPS).	We thank Professor Mark Brincat, Mr Raymond Gatt, Dr Anthony Fionini, Dr Raymond Galea, Dr Heidi Gauci Grech and the staff at the Bone Density Unit, Gynaecology Outpatients, Orthopaedic wards and Orthopaedic outpatients at Mater Dei Hospital and the staff at Karin Grech Rehabilitation Hospital for their assistance with recruitment of research subjects; Dr Pierre Demicoli and Dr Thomas Fuerst regarding BMD data; Dr Stephanie Bezzina-Wettinger, Dr Rosienne Farrugia and Dr Joseph Borg for technical assistance. We also thank all the women who participated in our study. Financial support was received from the European Union Strategic Educational Pathways Scholarhip scheme (STEPS).
**MrOS Sweden**	Financial support was received from the Swedish Research Council (K2010-54X-09894-19-3, 2006-3832), the Swedish Foundation for Strategic Research, the ALF/LUA research grant in Gothenburg, the Lundberg Foundation, the Torsten and Ragnar Söderberg's Foundation, the Västra Götaland Foundation, the Göteborg Medical Society, the Novo Nordisk foundation and the European Commission grant HEALTH-F2-2008-201865-GEFOS.	Financial support was received from the Swedish Research Council (K2010-54X-09894-19-3, 2006-3832), the Swedish Foundation for Strategic Research, the ALF/LUA research grant in Gothenburg, the Lundberg Foundation, the Torsten and Ragnar Söderberg's Foundation, the Västra Götaland Foundation, the Göteborg Medical Society, the Novo Nordisk foundation and the European Commission grant HEALTH-F2-2008-201865-GEFOS.
**NOSOS**		We are grateful to everyone who helped with NOSOS study recruitment, data and sample collection (radiographers, research nurses, administration staff, clinicians, technicians and research assistants) at both the Aberdeen and Dingwall centres, as well as Dr Malcolm Steven, who assisted with original participant access at the Dingwall centre. Finally, we are extremely grateful to all the women who took part in this study.
**OAS**	World Anti-Doping Agency, the Danish Ministry of Culture, Institute of Clinical Research of the University of Southern Denmark.	Eveline Boudin is thanked for managing DNA samples and the World Anti-Doping Agency, the Danish Ministry of Culture, and Institute of Clinical Research of the University of Southern Denmark are thanked for financial support.
**OSTEOS**		We thank all participants for given their consent to the study
**OPRA**	This work was supported by grants from the Swedish Research Council (K2012-52X-14691-10-3), FAS (2007-2125), Greta and Johan Kock Foundation, A. Påhlsson Foundation, A. Osterlund Foundation, the H Järnhardt foundation, King Gustav V and Queen Victoria Foundation, Åke Wiberg Foundation, Thelma Zoegas Foundation, The Swedish Rheumatism Association, Skåne University Hospital Research Fund, Research and Development Council of Region Skåne, Sweden.	Thanks are extended to the research nurses at the Clinical and Molecular Osteoporosis Research Unit, Malmö and to all the women who kindly participated in the study.
**SLO-PREV**	Slo-preval cohort was created as part of projects financialy supported by Slovenian research agency: P3-298 Geni, Hormoni in osebnostne spremembe pri hormonskih motnjah; Z1-3238: Genski in okoljski dejavniki tveganja za razvoj motnje pri remodelaciji kosti; , J2-3314 Genetski faktorji in hormoni pri presnovnih boleznih and J3-2330 Genetski dejavniki pri osteoporozi.	We are grateful to clinicians, researchers, technicians and nurses at Faculty of pharmacy and Clinical centre worked at subject's recruitments and sample processing. Especially we are grateful to prof. Andreja Kocijančič who was founder of osteoporosis research in Slovenia.
**TWINGENE**	We thank Martha Widger and Cody Xuereb for research assistance. This work was supported in part by the Ragnar Söderberg Foundation (E9/11); the National Science Foundation (EArly Concept Grants for Exploratory Research:	We express our profound thanks to all the study participants who contributed to this research

Cohort	Funding agencies	Full Acknowledgments
	"Workshop for the Formation of a Social Science Genetic Association Consortium", SES-1064089) as supplemented by the National Institutes of Health's (NIH) Office of Behavioral and Social Sciences Research, and the National Institute on Aging/NIH through Grants P01-AG005842, P01-AG005842-20S2, P30-AG012810, and T32-AG000186-23 to the National Bureau of Economic Research. The Swedish Twin Registry is supported by the Swedish Department of Higher Education, the European Commission European Network for Genetic and Genomic Epidemiology [ENGAGE:7th Framework Program (FP7/2007-2013)/Grant agreement HEALTH-F4-2007-201413; and GenomEUtwin: 5th Framework program "Quality of Life and Management of the Living Resources" Grant QLG2-CT-2002-01254]; the NIH (DK U01-066134); the Swedish Research Council (M-2005-1112 and 2009-2298); the Swedish Foundation for Strategic Research (ICA08-0047); the Jan Wallander and Tom Hedelius Foundation; and the Swedish Council for Working Life and Social Research. .	
**UFO**	The Umeå Fracture and Osteoporosis Study (UFO) is supported by the Swedish Research Council (K20006-72X-20155013), the Swedish Sports Research Council (87/06), the Swedish Society of Medicine, the Kempe-Foundation (JCK-1021), and by grants from the Medical Faculty of Umeå Unviersity (ALFVLL:968:22-2005, ALFVL:-937-2006,ALFVLL:223:11-2007, ALFVLL:78151-2009) and from the county council of Västerbotten (Spjutspetsanslag VLL:159:33-2007)	The Umeå Fracture and Osteoporosis Study (UFO) is supported by the Swedish Research Council (K20006-72X-20155013), the Swedish Sports Research Council (87/06), the Swedish Society of Medicine, the Kempe-Foundation (JCK-1021), and by grants from the Medical Faculty of Umeå Unviersity (ALFVLL:968:22-2005, ALFVLL:937-2006, ALFVLL :223:11-2007, ALFVLL:78151-2009) and from the county council of Västerbotten (Spjutspetsanslag VLL:159:33-2007). We thank Åsa Ågren, Hubert Sjödin and Magnus Hellström for skull full data processing and for help creating the UFO database and Kerstin Enquist and Ann-Marie Åhrén for their help in preparing samples for genotyping. We would also like to thank the participants and staff from the NSHDS cohort study and all collaborators in the UFO study group.

.
